# Architecture, Function, Regulation, and Evolution of α-Glucans Metabolic Enzymes in Prokaryotes

**DOI:** 10.1021/acs.chemrev.3c00811

**Published:** 2024-04-12

**Authors:** Javier O. Cifuente, Christophe Colleoni, Rainer Kalscheuer, Marcelo E. Guerin

**Affiliations:** 1 Instituto Biofisika (UPV/EHU, CSIC), University of the Basque Country, E-48940 Leioa, Spain; 2 University of Lille, CNRS, UMR8576-UGSF -Unité de Glycobiologie Structurale et Fonctionnelle, F-59000 Lille, France; 3 Institute of Pharmaceutical Biology and Biotechnology, Heinrich Heine University, 40225 Dusseldorf, Germany; 4 Structural Glycobiology Laboratory, Department of Structural and Molecular Biology, Molecular Biology Institute of Barcelona (IBMB), Spanish National Research Council (CSIC), Barcelona Science Park, c/Baldiri Reixac 4-8, Tower R, 08028 Barcelona, Catalonia, Spain

## Abstract

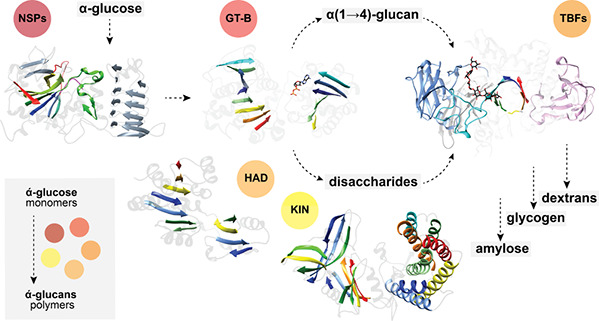

Bacteria have acquired sophisticated mechanisms for assembling and disassembling polysaccharides of different chemistry. α-d-Glucose homopolysaccharides, so-called α-glucans, are the most widespread polymers in nature being key components of microorganisms. Glycogen functions as an intracellular energy storage while some bacteria also produce extracellular assorted α-glucans. The classical bacterial glycogen metabolic pathway comprises the action of ADP-glucose pyrophosphorylase and glycogen synthase, whereas extracellular α-glucans are mostly related to peripheral enzymes dependent on sucrose. An alternative pathway of glycogen biosynthesis, operating via a maltose 1-phosphate polymerizing enzyme, displays an essential wiring with the trehalose metabolism to interconvert disaccharides into polysaccharides. Furthermore, some bacteria show a connection of intracellular glycogen metabolism with the genesis of extracellular capsular α-glucans, revealing a relationship between the storage and structural function of these compounds. Altogether, the current picture shows that bacteria have evolved an intricate α-glucan metabolism that ultimately relies on the evolution of a specific enzymatic machinery. The structural landscape of these enzymes exposes a limited number of core catalytic folds handling many different chemical reactions. In this Review, we present a rationale to explain how the chemical diversity of α-glucans emerged from these systems, highlighting the underlying structural evolution of the enzymes driving α-glucan bacterial metabolism.

## 1 Introduction to Glucans in Chemistry and Biology

### 1.1 Historical Perspective

Glucans are glucose homopolymers that cumulatively represent one of the largest deposits of biological carbon in nature. These ubiquitous polymers, whose primary examples are cellulose, starch, and glycogen, play essential roles in the carbon cycle and biosphere transformations. The omnipresent nature of these polysaccharides makes us overlook they are the main constituent of natural raw materials gathered by man since prehistoric times; glucans are the main energy component in food crops and the main constituent of wood for fuel and shelter. Thus, the transformation of glucan materials spans the whole of human existence. Early records on the extraction of cellulose fibers for flax linen, fermentation of starchy grains for beer brewing, and purification of starch used as glue or cosmetic powder can be traced to ancient civilizations. Arguably, uncountable observations of natural and man-made glucan transformations predate modern investigations.

The beginning of glucan chemistry can be stated with the discovery of glucose as a substance purified from raisins by Andreas Marggraf in 1747 and the subsequent discovery of the conversion of starch to glucose by acids by Gottlieb Kirchhof in 1811.^1^ In 1833, Anselme Payen and Jean-François Persoz isolated a substance that accelerated the transformation of starch into maltose, the diastase, which was the first enzyme produced in concentrated form. In 1860, Pierre Berthelot isolated the enzyme invertase that hydrolyzes sugar cane into glucose and fructose. In 1837, Payen also discovered cellulose composed of glucose residues and isomeric with starch.^2^ Jacob Berzelius, who coined the concepts of catalyst and polymers, aware of Kirchhof and Payen’s findings, stated, “...chemical processes in living nature, we regard them in a new light. For example, since nature has placed diastase around the eyes of potatoes...we find that the insoluble starch in the tuber is changed to gum and sugar by catalytic power...” and “...One can hardly assume that this catalytic process is the only one...”^3^ These early findings paved the way to advance the view of life based on enzyme-driven chemical transformations.

Most of the early observations on the biochemistry of glucans were extracted from fermentation studies. Louis Pasteur discovered the production of dextran during the fermentation of wine, which Philippe van Tieghem later assigned to a bacterial activity.^4^ Adrian Brown found that bacteria can also synthesize cellulose.^5^ In 1865, Claude Bernard discovered glycogen as an energy reserve substance in liver tissue.^6^ The occurrence of glycogen in bacterial cells was later confirmed by Arthur Meyer.^7^ Wilhelm Kühne advance the concept of the separation between a ferment (zyme) and the active component for these conversions, the enzymes. In 1878, Wilhelm Kühne used for the first time the word “enzyme” to describe the ability of yeast to produce alcohol from sugars. In 1897, Eduard Buchner discovered that yeast extract with no living cells can form alcohol from a sugar solution. The conclusion was that biochemical processes do not necessarily require living cells, but are driven by special substances, enzymes, formed in cells, ending the “vitalist” view of living processes.

The modern vision of glucan and sugar biochemistry was established early in the 20th century. Emil Fisher in his Nobel lecture already stated that polysaccharides were nothing other than the glucosides of the sugars.^8^ This idea was confirmed by Walter Haworth, Edmund Hirst, and co-workers with the first description of the chemical architecture of several glucans, among other polysaccharides.^9,10^ Later, Haworth received the Nobel Prize in Chemistry for his work on carbohydrates, the structure of complex sugars, and the structure of Vitamin C. Previously in 1894, Fisher proposed the “lock-and-key” model for enzyme function,^11−13^ and soon after, Leonor Michaelis and Maud Menten presented their seminal work on enzyme kinetics working on invertase.^14^ Jakub Parnas and Tadeusz Baranowski discovered phosphorolysis in glycogen metabolism,^15^ while Arthur Harden and Hans von Euler-Chelpin used fermentation of sugar and fermentative enzymes, identifying the role of phosphate in accelerating sugar fermentation, receiving the Nobel Prize in Chemistry 1929.^16,17^ Harden also reported that acellular fermentation was maintained due to the presence of glycogen, needing a factor “co-zymase”, the NADH.^18^ The studies of Gustav Embden, Otto Meyerhof, and Parnas on glucose and glycogen fermentation were critical in articulating the first reported pathway: glycolysis.^19^ Later in 1952, an alternative glycolytic pathway was described by Nathan Entner and Michael Doudoroff in *Pseudomonas saccharophila*.^20^ Charles Hanes observed the differential endo- and exohydrolytic action of α- and β-amylases.^21^ He also proposed the first tridimensional structure of a macromolecule, the helical amylose, and synthesized the first macromolecule *in vitro*: starch.^22^

Glycogen study was greatly propelled by Gerty Cori and Carl Cori, who reported the very first purification to homogeneity of a glycogen phosphorylase (GP).^23^ They discovered that GP can catalyze both the synthesis and degradation of glycogen, being activated by AMP.^24^ This activation was the first report of an allosteric effector, a concept developed by Jacques Monod, Jeffries Wyman, and Jean-Pierre Changeux.^25^ In 1956, Edwin Krebs and Edmond Fischer reported the discovery of the GP-kinase, revealing the importance of posttranslational modification in metabolic regulation.^26^ Meanwhile, Luis Leloir and co-workers revealed the origin of glycogen and starch metabolism with the discovery of the sugar-nucleotides, their synthetic enzymes, including the glycogen synthase.^27,28^ Earl Sutherland reported the mechanism of hormone-induced glycogen breakdown in the liver, linking the action of the endocrine system with glycogen metabolism.^29^

Altogether, glucans markedly impact and transverse our understanding of key chemical transformations in living matter. With this perspective, we review from the fundamentals of glucan chemistry to the biology of these polymers, providing a framework to conceptualize how the chemical diversity of α-glucans emerges from the structural evolution of the enzymatic machinery in bacteria.

### 1.2 The Origin of Glucan Unit: d-Glucose

**Figure 1:**
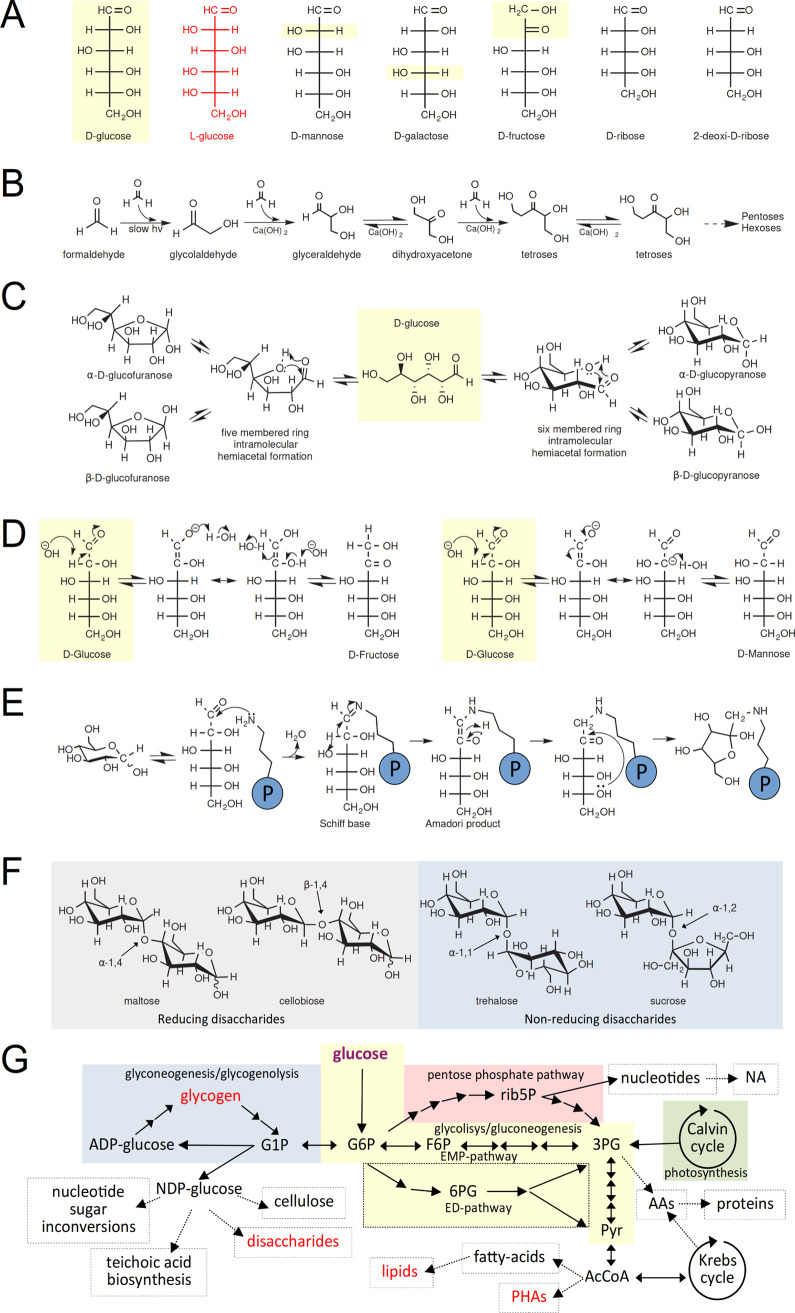
Glucose, the central sugar in life. (A) Fischer projections of d-glucose (yellow shade), its non-biological occurring stereoisomer l-glucose (red), and the most common monosaccharides, hexoses and pentoses, found in nature. Differences between d-glucose with close related hexoses, d-mannose, d-galactose, and d-fructose, are highlighted in yellow. Note that the orientation of the last stereocenter is responsible for the d- and l-denominations of these sugars. (B) A schematic sequence of reactions of formaldehyde to form glycolaldehyde and subsequent aldoses and ketoses, the so-called formose reactions, that have been proposed as the abiotic origin of sugars. (C) Equilibrium of cyclization of linear d-glucose in solution. Glucose cyclizes, and the hydroxyl group on either position C4 or C5 undergoes an intramolecular reaction with the C1 carbonyl group of the aldehyde. As a result, the product formed is a hemiacetal resulting in either a 5- or 6-membered ring, in which the resulting hydroxyl group could present two orientations α- or β- concerning the ring plane. (D) Reactivity of d-glucose in solution leads to common sugars (i) d-fructose by an enediol rearrangement, and (ii) epimerization to d-mannose via an enolate intermediate. (E) Damaging of d-glucose with proteins, showing the reaction with lysine side chains via Schiff base formation and Amadori rearrangement, which ends in cyclic fructosamine. (F) Structure of common glucose disaccharides, showing maltose and cellobiose reducing disaccharides (grey shade) free anomeric hydroxyl group that can undergo a reducing reaction, while non-reducing disaccharides trehalose and sucrose (blue shade) compromise both anomeric carbons, therefore unavailable for reaction. (G) Overall metabolism of glucose. The glycolic and gluconeogenesis pathways (yellow), showing the classic Embden–Meyerhof–Parnas pathway (EMP) and its variant Entner–Doudoroff pathway (ED; dashed box). This pathway integrates main pathways such as the Pentose phosphate pathway (PP; red shade), the tricarboxylic Krebs cycle, the photosynthetic Calvin cycle (green shade), and the glyconeogenesis/glycogenolysis pathways (blue shade). Other metabolic pathways are shown linked to this central energy metabolism (black dashed). Other molecules can serve as energy deposits (red letters). It is worth noting that glycogen is the energy closest storage molecule, directly linked to the initial point of glycolysis.

The emergence of sugars under abiotic conditions from formaldehyde onto alumina and aluminosilicates into monosaccharides represents a model that could explain the primordial origin of these molecules.^30^ Sugars could also have been interconverted before living organisms arose, possibly giving rise to various types of monosaccharides.^31,32^ In present living organisms, 3 of the 16 possible aldohexoses are physiologically relevant, d-glucose, d-galactose, and d-mannose, all of which displaying d-isomery (Figure 1). d-Glucose was selected by nature as the central molecule in energy metabolism (Figure 1A). d-Glucose is the most abundant in all life kingdoms, pointing to a central role of this sugar at the origin of life (Figure 1B). There is no final understanding on how hexoses d-isomery prevailed in nature, and how d-glucose became the most common monosaccharide in life. d-Isomery asymmetry may have been selected and perpetuated with the emergence of proteo- or ribo-enzymes,^33^ as with other synthetic asymmetric catalysts.^34^ Nevertheless, symmetry-breaking events appear to be a distinct possibility within self-organizing chemical systems. This suggests that homochirality might have been a prevalent trait among the initial biopolymers, possibly evolving alongside their self-replication capabilities at the origin of protometabolism.^33^ The high intrinsic stability of d-glucose likely played a role in its selection. It is worth noting that the keto-hexose fructose can be converted to stable aldohexoses, i.e., glucose and mannose (2-epi-glucose), by Lobry de Bruyn-Van Ekenstein rearrangement^35^ (Figure 1D). Interestingly, d-glucose synthesis (gluconeogenesis), glycolysis, and the pentose pathways presumably existed in prebiotic metabolism as they have been assessed to be generated in abiotic conditions.^36^ In addition, genomic analysis of enzymes of the Embden-Meyerhof-Parnas pathway from archaea and hyperthermophilic bacteria support a gluconeogenic origin of metabolism.^37^ Arguably, the selection and centrality of d-glucose occurred in the transition between prebiotic and biotic world.

### 1.3 d-Glucose Biochemistry

d-Glucose participates in several acid–base catalyzed reactions, including mutarotation, enolization, and β-elimination, and also has reducing power. d-Glucose generally presents a cyclic pyranose conformation in equilibrium with minor amounts of tautomeric linear and cyclic furanose forms. The cyclization results from an intramolecular hemiacetal formation whose hydroxyl group can take two anomeric positions leading to the α-d-glucose and β-d-glucose forms that exist in a mutarotation equilibrium (Figure 1C). The β-d-glucose predominates because the hydroxyl group of the anomeric carbon is in the more stable equatorial position. Notably, the hemiacetal confers a highly reactive character to the C1 that often participates in d-glucose enzymatic transformations. d-Glucose is the preferred energy source through glycolysis and the Krebs cycle, mediating the biosynthesis of several key compounds in the cell (Figure 1G). d-Glucose is converted into other major hexoses as d-fructose by isomerization, and d-galactose and d-mannose by epimerization. In addition, d-glucose is converted via the pentose pathway to d-ribose, a central constituent of nucleic acids, while fatty acids and most amino acids can be synthesized from d-glucose. Therefore, d-glucose is a molecule that plays a central role in producing all major components of the cell: proteins, lipids, nucleic acids, and polysaccharides. Supporting this notion, most organisms can synthesize d-glucose *de novo* via gluconeogenesis to maintain homeostasis.^38^ In addition, several microorganisms can grow using d-glucose as the sole energy and carbon source.^39^ Despite this importance, it is worth noting that free d-glucose accumulation in the cell can be harmful to the cell machinery due to its reactivity favors the formation of non-enzymatic covalent adducts with proteins and DNA via Schiff base and Amadori rearrangement^40,41^ (Figure 1E). In addition, high levels of intracellular glucose induce high osmotic pressure that is not compatible with cellular life. From an evolutionary perspective, the acquisition of mechanisms to safely store d-glucose may provide an advantage to a living organism when competing for environmental glucose.

### 1.4 Glucose Disaccharides

The condensation of monosaccharides to form disaccharides is the minimal event for the polymerization of sugars. The most common disaccharides comprise at least one d-glucose moiety, possibly due to the abundance of this monosaccharide in nature (Table 1). The biosynthesis of disaccharides involves the linkage between the anomeric carbon of a first sugar with any of the hydroxyl groups of a second monosaccharide forming a glycosidic bond. In principle, since the bonding can involve hydroxyl groups in different positions and in two anomeric configurations, the condensation between two monosaccharides can produce an extensive disaccharide repertoire. According to their redox capacity, disaccharides are classified in (i) reducing, where only one anomeric carbon is compromised in the linkage, and (ii) non-reducing, where both anomeric carbons are linked to each other (Figure 1F).

**Table 1:** Most Common Glucose Disaccharides

	Name	Monosaccharide 1 (glycosyl)	Monosaccharide 2	Glycosidic bond
Non-reducing	Trehalose	Glucose	Glucose	α(1→1)α
	Sucrose	Glucose	Fructose	α(1→2)β
Reducing	Maltose	Glucose	Glucose	α(1→4)
	Isomaltose	Glucose	Glucose	α(1→6)
	Cellobiose	Glucose	Glucose	β(1→4)
	Lactose	Galactose	Glucose	β(1→4)

The metabolism and function of disaccharides containing d-glucose are diverse across organisms. The non-reducing disaccharides trehalose and sucrose play a central role in energy storage in several living organisms due their stability,^42^ also having properties that aid the cells in enduring environmental stress, such as protecting membranes and proteins from freezing and dehydration.^43^ Furthermore, due to their small size, disaccharides are used to transport carbohydrates; trehalose is present in high concentration in insects’ hemolymph, while sucrose is transported in plants’ phloem. Trehalose synthesis is widely distributed in nature and found in Bacteria, Archaea, and Eukaryota.^44,45^ Sucrose synthesis is restricted to plants and some photosynthetic bacteria,^46,47^ while most organisms can use it as a carbon and energy source. In contrast, disaccharides with a reducing end are not the best suited as storage compounds due to their reactive nature. This is the case for maltose and cellobiose, disaccharides that originate from the breakdown of glucans,^48,49^ and lactose, a special reducing disaccharide synthesized exclusively by mammals as the main energy component of milk, also fermented by microorganisms.^50^

### 1.5 Glucose Polysaccharides

**Figure 2:**
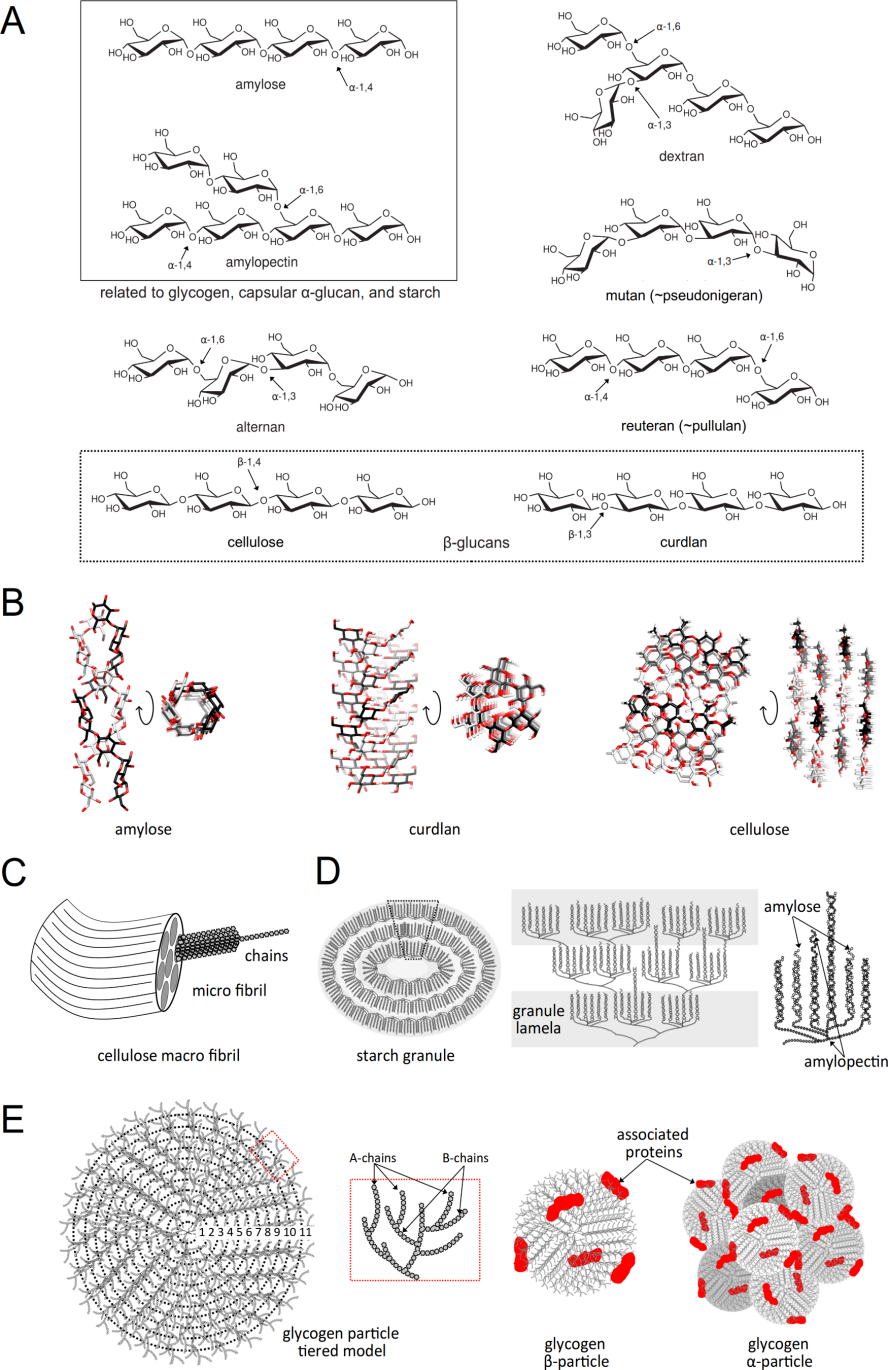
Structure of glucans. (A) Chemical structure of common glucans produced by bacteria. Arrows indicate the main bonds found in amylose, amylopectin, dextran, altenan, and mutan, all representative α-glucans. Amylose and amylopectin are framed by a continuous line, indicating their structures can be found in glycogen, capsular α-glucans, and starch. Structures of cellulose and curdlan (framed by a dotted box) are also presented as examples of β-glucans. (B) Two perpendicular views of the three-dimensional structures obtained by X-ray crystal diffraction of (i) the double helical structure of the α-glucan amylose, (ii) the triple helical arrangement of curdlan, and (iii) the linear chains of cellulose, showing individual glucan chains in black, grey, and white carbon bonds. Structures have been obtained from PolySac3DB (https://polysac3db.cermav.cnrs.fr/home.html). (C–E) The biological organization of glucans. (C) Diagram showing the structural arrangement of macro and microfibrils formed by cellulose chains. (D) Diagrams showing the structural arrangement of starch, from left to right, (i) the starch granule, (ii) the lamellas, and (iii) the amylose and amylopectin complex. (E) Diagrams showing the structural arrangement of glycogen, from left to right, (i) the tiered model of glycogen, comprising the non-branched A-chains and branched B-chains (dashed red box), and (ii) the β-particle and its assembly product, the α particle. Proteins associated with particles are shown as red spots.

Glucans are broadly classified according to the anomeric configuration of the d-glucose moieties in α-glucans, β-glucans, and mixed α/β-glucans (Figure 2).^51^ Glucans can also be classified as branched or unbranched polysaccharides according to the presence/absence of ramifications.^51^ Glucans can adopt a high degree of structural complexity, such as the ramified glycogen comprising α(1→4) backbones with α(1→6) branches while its topoisomer starch is composed of two different α-glucans, amylose, a linear unbranched α(1→4)-glucan, and amylopectin, a branched chain containing α(1→4) and α(1→6)-glucose linkages (Figure 2A). It is worth noting that, under a common denomination, a particular glucan can display structural variability, size, or branching level due to organism-specific biosynthetic machinery or the metabolic state of the organism.^52,53^ α-Glucans are generally viewed as polymers with an energy storage function in the cell, like glycogen and starch; however, they also play other important roles, including structural roles as exopolysaccharides, or as cell wall components. Similarly, β-glucans are usually associated with extracellular structural functions. This is well represented by cellulose, the most abundant biomolecule in the biosphere, and the main component of the plant cell wall (Figure 2A,B). β-Glucans can play other functions, including (i) cellulose as a fibrous structural component of bacterial biofilms, it forms a mechanically strong hydrogel with high water adsorption capabilities,^54^ (ii) cyclic β-glucans act as messengers in plant-microbe interactions,^55^ (iii) internal and external energy storages.^56^ The fungal cell wall is composed of a polysaccharide-based three-dimensional network that is continuously adapting to growth and environmental conditions and is essential for cell survival (1). The central core consists of a branched β(1→3)-glucan with 3% to 4% interchain linked (via β(1→4)-linkage) to chitin. Laminarin, a branched β(1→3)-glucan from brown algae, and paramylon, a β(1→3)-glucan synthesized by the flagellate *Euglena*, both are internal energy reserves with parallel function to glycogen. The curdlan-like β(1→3)-glucan exopolysaccharides are used as external energy storage molecules in *Cellulomonas flavigena*(57) (Figure 2A,B).

The chemistry and resulting architecture of glucans can certainly be correlated to the biological role they play. For instance, in (1→4) linkages, the α(1→4) anomeric configuration results in a helical structure of amylose, a component of glycogen and starch, while the β(1→4) anomeric configuration results in the classical straight chain polymer of cellulose (Figure 2B). This difference makes cellulose perfectly insoluble,^58^ forming crystalline matrices and fibers with high tensile strength and resistance to enzymatic digestion (Figure 2B,C), desired properties of a structural component. It is worth noting that the extra degrees of freedom provided by the rotation about the C5 and C6 bonds gives (1→6) linked homoglucans higher solution entropy values (Figure 2A).^59^ Altogether, glucan functions concern not solely the anomeric configuration (α or β) of the polymer but also the architecture and cellular localization. In the following sections, we will focus on α-glucans and related metabolism to discuss the biological machinery that generates their diversity.

## 2 Architecture of α-Glucans

### 2.1 Overview of Eukaryotic and Prokaryotic α-Glucans and Their Localization

Glycogen represents a form of soluble α(1→4)-glucan comprising α(1→6) branches of bacterial or heterotrophic eukaryotic origin^60^ (Figure 2A), whose main characteristic is to form non-crystalline particles with a wide range of size. Overall, glycogen synthesis is mainly based on the use of nucleotide-diphospho-sugar (NDP-sugar) donors, UDP-glucose in heterotrophic eukaryotes, and ADP-glucose in bacteria.^61^ In eukaryotic cells, glycogen particles present a buried protein, glycogenin (GN), that acts as the primer for glycogen synthesis, remaining covalently bound at the particle core.^62^ Different types of glycogen particles are observed in eukaryotic cells, classified as α-, β-, and γ-particles, representing different levels of α-glucan polymers organization^63^ (Figure 2E). The β-granules are individual glycogen particles comprising several protein-rich γ-particles that act as subunits, while α-granules are a structure of clustered β-granules glued together by the GN.^64^ The occurrence of “glycosomes”, dedicated dynamic organelles for glycogen metabolism, has been suggested,^65^ comprising a glycogen-protein complex, where the protein component provides the enzymatic machinery of the organelle, and glycogen is the product of its synthetic activity.^65^ Glycogen is also present as α- and β-granules in several bacterial species, including mycobacteria, streptomyces, and enterobacteria.^66,67^ However, since bacterial glycogen does not present any protein content to cluster the β-particles, the similar shape of eukaryotic and prokaryotic granules seems to appear as a result of convergent evolution.^66^ Starch can be considered a topoisomer of glycogen, primarily known for its ability to create insoluble crystalline structures. The starch granule is insoluble in water and densely packed, but still accessible to the plants’ metabolic enzymes. Plants and green algae form starch in plastid compartments such as the chloroplast of the leaf or amyloplasts.^68^ Plant starch consists of two types of molecules tightly clustered together, the linear α(1→4)-amylose and amylopectin containing α(1→6)-linked branches of α(1→4)-glucan^69^ (Figure 2D). One of the low energy conformations of the flexible amylose chain leads to single strands that readily form rigid double helices. These double helices associate in pairs, stabilized by hydrogen bonds and van der Waals forces. These pairs associate to give the A or B structures, depending on their chain length and water content.^70^ On the other hand, Floridian starch is present in the cytoplasm of glaucophytes and red algae, forming crystalline granules with radially oriented fibrils and concentric layers of amylopectin.^71,72^ The metabolism of these two types of starch relies on different nucleotide sugar donors, ADP-glucose for the plastidial plant starch and UDP-glucose for the cytosolic Floridian starch.^73^ Interestingly, in cyanobacteria, α-glucan was observed in two forms, glycogen or crystalline semi-amylopectin, also called cyanobacterial starch,^74,75^ indicating an evolutionary transition from soluble α-glucan states toward crystalline α-glucan storages in photosynthetic organisms. These pieces of evidence in the evolution of organisms show that starch metabolism is important to trace the cyanobacterial origin of plastid endosymbionts into photosynthetic eukaryotes.^76−78^

α-Glucans are major components of the cell wall, capsules, or slimes on the exterior of fungi and bacterial cells, displaying diverse α-linkages.^79^ Fungi present several types of α-glucan polysaccharides as extracellular or cell wall components, mostly forming linear backbones, some made only with homogeneous linkages, such as α(1→3), α(1→4), or α(1→6), and also mixed linkages of alternating α(1→3) and α(1→6),^80^ like in alternan (Figure 2A). Solid-state NMR studies showed that α-glucans associations with other polysaccharides allow the formation of a rigid and impermeable scaffold protecting fungal cells from external stresses.^81^ Bacteria present two main types of α-glucans outside the cell envelope, capsular α-glucans and dextrans.^82,83^ The capsular α-glucans are present in Actinobacteria and resemble glycogen but with shorter α(1→4) linear chains,^84^ while dextrans refer to a large and diverse group of α-glucans with different linkages, including dextrans, alternans, mutans, and reuteran, that form polymer matrix biofilms providing protection to colonizing bacteria^85−87^ (Figure 2A).

### 2.2 The Architecture of α-Glucans in Bacteria

#### 2.2.1 The Cytosolic Bacterial Glycogen

Glycogen represents the most important intracellular carbon and energy storage polymer in bacteria. Glycogen follows the three principles for a compound to be considered an energy reserve: (i) the compound accumulates when there is a surplus in energy, (ii) the compound is used when the energy supply from exogenous sources is insufficient for optimal maintenance of the cell, and (iii) the compound provides an advantage compared with an organism lacking this storage mechanism.^85,88^ Glycogen particles accumulate in bacteria preponderantly during the stationary phase, in the presence of an excess carbon source, and under environmental conditions of slow growth or no growth,^89−91^ playing a major role in awakening from dormancy.^92^*Escherichia coli* mutants lacking functional enzymes associated with glycogen biosynthesis can grow as well as their wild type parent strains, indicating that glycogen is not required for bacterial growth.^88^ However, glycogen accumulation prolongs the survival rate, resistance to starvation, low temperatures, and desiccation of bacteria compared to mutants without glycogen.^93−95^ These observations suggest that under conditions of no available carbon source, glycogen is probably utilized to preserve cell integrity, providing the energy required by the bacteria for maintenance.^96^

The glycogen architecture and chemical structure were originally investigated and discussed based on animal glycogen and plant starch. Early investigations based on stepwise enzymatic degradation^97,98^ revealed that these α-glucan polysaccharides are composed of irregular tree-like structures as originally proposed by Meyer^99^ instead of comb-like (each side branch arises from a single main branch) or laminated structures (branch arises from a preceding branch). This tree-like structure is composed of linear chains of α(1→4)-linkages with α(1→6)-linkages at branching points with an apparent random arrangement,^66^ in contrast to the high-ordered structures required to form crystalline arrays as observed in starch. The α(1→4)-linked chains’ average span comprised 8–12 glucosyl residues.^91,100^ This short chain length enhances bacterial viability by altering glycogen degradation rate.^101^ Bacterial and eukaryotic glycogen branching account for 7–10% of the total linkages.^78^ As a consequence, the branched polymer results in a highly water-soluble 3D fractal-like structure,^102^ allowing the storage of large amounts of glucose, without causing osmotic stress. Specifically, the high number of terminal non-reducing glucose units are readily accessible to hydrolytic enzymes in case the bacterium needs energy because of starvation. Biophysical characterization and visualization of glycogen extracted from bacteria by electron microscopy revealed a size of ca. 20 nm for β-particles and ca. 40 nm for α-particles, the latter showing a rosette-like appearance (Figure 2).^66^ α-Particles can break into β-particles. α-Particles can show two structural states, (i) fragile (high-density) and (ii) stable (low-density).^66^ In animals, it has been observed that the small β-particles degrade more easily to glucose than α-particles.^103^ Recent studies in bacteria indicate that α-particles states are modulated by the association of enzymes, accounting for fragile and stable states due to changes in average chain length, allowing for the control of glycogen storage and degradation.^104^

The internal structure and architecture of the glycogen particle were first conceptualized by the “tiered model” based on the molecular arrangement of the α-glucan presenting two chain types: unbranched A-chains and branched B-chains^105−107^ (Figure 2E). Specifically, branches in B-chains are uniformly distributed, comprising two branches that generate further A- or B-chains. Mathematical calculations indicate that glycogen can arrange in up to 12 concentric tiers.^108^ This branching spherical growth of the particle leads to a progressively more packed structure toward the periphery allowing only A-chains in the most external tier while preventing the addition of further tiers due to the lack of space for the enzymes to process the polysaccharide, therefore self-limiting the size. Thus, the glycogen particle displays a molecular size between 10^7^ and 10^8^ Da comprising ca. 55,000 glucose units distributed in 12 tiers, and 20 to 50 nm in size.^107–109^ While the “tiered model” offers a fascinating perspective on understanding the organization of glucan chains within glycogen particles, it is worth noting that a combination of experimental and computational approaches has challenged the fractal-like organization of glycogen particle.^102^ Surprising findings from small angle X-ray scattering analysis (SAXS) of β-particles have revealed a high density at the center of particles, contradicting the notion of a fractal-like organization and instead suggesting a randomly branched polymer.^110^ Recent Monte Carlo simulations of the glycogen particle biosynthetic process support these experimental data, showing that enzymatic activities and hindrance may control the chain length, radial density distributions, and particle size.^111^

#### 2.2.2 The Chemical Structure of α-Glucans as Extracellular Components

**Figure 3:**
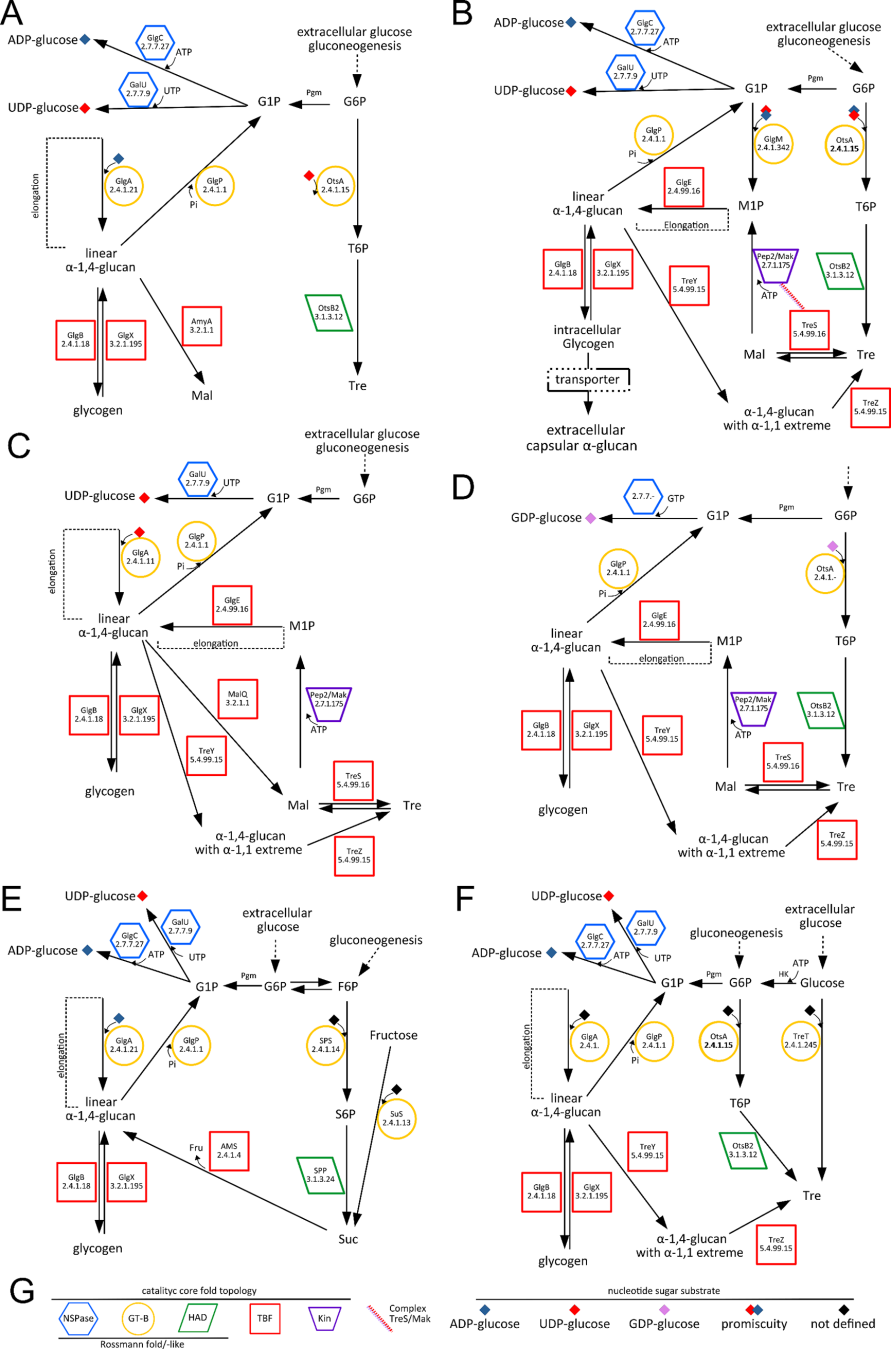
Diversity of metabolic arrangements for α-glucan and disaccharide pathways in the context of prokaryotes. (A) The classical glycogen metabolism (GlgC-GlgA-GlgB) and independent trehalose metabolism (OtsA-OtsB) pathways in *E. coli*. (B) The glycogen metabolism via maltose 1-phosphate (M1P; GlgM-GlgE) is connected with the production of extracellular capsular α-glucan in *M. tuberculosis*. Essential wiring of the trehalose metabolism (TreS-Pep2/Mak; TreZ-TreY) permits the redundancy for the production of trehalose or α-glucan. (C) The glycogen metabolism in *P. aeruginosa* highlights the use of an UGPase (lack of GlgC/AGPase) for the GlgA-dependent biosynthesis of α(1→4)-glucan. It is worth noting the presence of GlgE, concurrent in the synthesis of α(1→4)-glucan via maltose 1-phosphate and TreS-Pep2/Mak. *P. aeruginosa* lacks a direct/dedicated pathway for the biosynthesis of trehalose, relying on maltose interconversions via the MalQ and TreZ-TreY pathways. (D) The glycogen metabolism of *S. venezuelae* relies on the trehalose pathway via an OtsA GDP-glucose-dependent synthesis. (E) An overall view of the cyanobacteria classical glycogen metabolism (GlgC-GlgA-GlgB). The sucrose synthesis is via SPS/SPP or SuS, and the alternative production of α(1→4)-glucan via amylosucrase (AMS). (F) An overall view of the archaea classical glycogen metabolism (GlgC-GlgA-GlgB). Note the alternative production of trehalose via TreT. (G) Charts showing the different folds of enzymes geometric symbols used for NDP-sugar pyrophosphorylases (blue hexagons), GT-B GTs (yellow circles), TIM barrel folds (red boxes), HAD domain phosphorylases (green parallelograms), and the maltokinase (violet trapezium). Nucleotide sugars are indicated with small colored rhombuses according to the legend.

Glycogen was first recognized as an intracellular polymeric material thought to function as an inert storage deposit for carbon and energy. In recent years, however, it became evident that some bacteria can deposit polymers with a glycogen-like architecture outside the cells. Furthermore, the synthesis and deposition of extracellular α-glucans exhibiting a variety of structures differing from glycogen have been long known from bacteria producing an extracellular matrix and forming biofilms. These extracellular α-glucans are either synthesized outside the cells employing secreted polymerases or are first synthesized intracellularly and subsequently secreted (Figure 3). Their structures and physicochemical properties differ strongly from intracellular α-glucans as they fulfill other functions outside of the cells (Table 2).

**Table 2:** Representative α-d-Glucans Chemical Diversity and Their Distribution in Nature

Eukaryotes	Name	Geometry	Backbone	Branching	Location/Function
Animals, fungi and protozoa	Glycogen	Branched	(1→4)-α-d-glucan	(1→6)-α-d-glucan	Intracellular. Cytosol. Storage
Higher plants and green algae	Starch amylopectin	Branched	(1→4)-α-d-glucan	(1→6)-α-d-glucan	Intracellular. Plastids. Storage
	Starch amylose	Linear	(1→4)-α-d-glucan		Intracellular. Plastid. Storage
Red algae, glaucophytes	Floridean starch	Branched	(1→4)-α-d-glucan	(1→6)-α-d-glucan	Intracellular. Cytosol. Storage
Fungi	Nigeran	Linear	Alternating (1→3)α(1→4)-α-d-glucan		Wall component
Fungi	Pseudonigeran	Linear	(1→3)-α-d-glucan		Wall component
Fungi	Pullulan	Linear	(1→4)α(1→4)(1→6)-α-d-glucan		Extracellular
Prokaryotes					
Bacteria	Glycogen	Branched	(1→4)-α-d-glucan	(1→6)-α-d-glucan	Intracellular. Cytosol. Storage
Archaea	Glycogen	Branched	(1→4)-α-d-glucan	(1→6)-α-d-glucan	Intracellular. Cytosol. Storage
Bacteria	Capsular α-glucan	Branched	(1→4)-α-d-glucan	(1→6)-α-d-glucan	Extracellular Capsular component
Bacteria	Dextran	Branched	(1→6)-α-d-glucan	α(1→2,3,4)	Exopolysaccharide
Bacteria	Alternan	Branched	Alternating α(1→3)α(1→6)- d-glucan	α(1→3)	Exopolysaccharide
Bacteria	Mutan	Branched	α(1→3)- d-glucan	α(1→6)	Exopolysaccharide
Bacteria	Reuteran	Branched	(1→4)-α-d-glucan, including α(1→6)	α(1→6)	Exopolysaccharide
Bacteria	Amylose	linear	(1→4)-α-d-glucan		Exopolysaccharide

#### 2.2.3 The Capsule of *Mycobacterium tuberculosis*

The ability to form a capsule surrounding the cells is a feature frequently found among pathogenic bacteria and, in most cases, an important virulence factor. In contrast to most other capsule-forming bacteria, the presence of an outermost capsular layer surrounding cells of the human pathogen *Mycobacterium tuberculosis* has been a matter of debate for many years. In contrast to typical bacterial capsules, the mycobacterial capsular layer is thin, not visible using light microscopy, and has a loosely-attached structure sensitive to agitation and the presence of detergents typically added to liquid culture media to minimize clumping of mycobacterial cells. More recently, ultrastructural studies employing advanced cryo-electron microscopy techniques could unambiguously prove the capsule’s existence in *M. tuberculosis* and visualize it in a close-to-native state.^112^ The mycobacterial capsule is mainly composed of neutral polysaccharides and additionally also contains proteins and lower amounts of lipids.^113,114^ The capsular polysaccharides identified in *M. tuberculosis* comprise three types of polymers: (i) a branched, high-molecular-weight α-d-glucan mainly comprising an α(1→4)-linked core with α(1→6)-branches every 5 or 6 residues by mono- and di- glucosides, with a molecular mass estimated to be ca. 100 kDa by gel permeation chromatography;^113−116^ (ii) a d-arabino-d-mannan heteropolysaccharide exhibiting an apparent molecular weight of 13 kDa;^117^ and (iii) a d-mannan homopolysaccharide with an apparent molecular weight of 4 kDa exhibiting α(1→6)-glycosidic linkages with α(1→2)-branches.^117^ Of the three mentioned polysaccharides, α-glucan is the major capsular polysaccharide constituent of *M. tuberculosis*, representing up to 80% of the extracellular polysaccharides.^113,115,116^ Using an α-glucan-specific monoclonal antibody, the production of extracellular α-glucan material has also been demonstrated to occur during infection for *M. tuberculosis* cells grown in mice.^118^ Structural analyses of the intracellular (i.e., glycogen) and extracellular α-glucans produced by slow-growing mycobacteria revealed a similar composition and architecture indicative of a common biosynthetic origin.^116,117^ However, depending on the analytical methods used, also differences were reported with capsular α-glucan possessing a higher molecular mass and a more compact spatial organization than the glycogen isolated from the same species, which has led to the speculation that specific enzymes might be responsible for the synthesis of each polymer.^115^ More recently, a combination of enzymatic characterizations and biochemical analyses of mutant strains resulted in the discovery of the metabolic network and configuration of pathways required for intra- and extracellular α-glucans in *M. tuberculosis*(119) (Figure 3B). This study unambiguously showed that both forms of α-glucan polymers are synthesized by the same enzymatic machinery. Synthesis occurs intracellularly, and a portion of the produced material is exported to yield the extracellular α-glucan that build up the capsule. As will be elaborated in more detail in section 3.2, α-glucan in *M. tuberculosis* is produced by iterative cooperation of the maltose 1-phosphate-dependent maltosyltransferase GlgE and the branching enzyme GlgB (Figure 3B).^84^ The polymer produced in this iterative process comprises C chains of DP ∼9, A and B chains of DP ∼7–8, and a mean number of branches per B chain of 1.2–1.6, which is considerably lower than the value of 1.8–1.9 reported for classical glycogens. Thus, the resulting molecule is a high-molecular weight glycogen-like polysaccharide of ∼5 × 10^6^ Da but has a much less arboreal structure compared to glycogen described from other bacteria and from eukaryotic organisms and exhibits an A:BC chain ratio that is the smallest reported for α-glucans.^84,120^ Both the intracellular and extracellular polymers isolated from *M. tuberculosis* cells comprised β-particles that have diameters ranging from ∼30 to ∼60 nm and occasionally aggregate into larger α particles. The synthetic material produced *in vitro* using purified *M. tuberculosis* GlgE and GlgB proteins and maltose 1-phosphate as a substrate formed β-particles with similar diameter and morphology as the biological polymers isolated from *M. tuberculosis* cells.^84^ With an intracellular biosynthetic origin of all α-glucans in *M. tuberculosis*, the existence of a transport mechanism(s) for translocation of the polymer to the capsular space has to be postulated. However, such an α-glucan transporter has not been identified yet. It is tempting to speculate that the peculiar structure of *M. tuberculosis* α-glucan with a reduced degree of branching and a less arboreal architecture might be a feature facilitating export. However, no reports are available yet addressing the impact of α-glucan structure on extracellular deposition. *M. tuberculosis* has been shown to release extracellular vesicles that originate from the cytoplasmic membrane and contain cytosolic cargo. While it is theoretically conceivable that α-glucan is packed into the lumen of such vesicles for secretion, ELISA employing an α-glucan-specific monoclonal antibody could not detect this polysaccharide in purified vesicle preparations.^121^ Finally, theoretically it is possible that capsular α-glucan is produced extracellularly by secreted GlgE and GlgB proteins in a nucleoside-sugar-free biochemical reaction. However, this would necessitate secretion of substantial amounts of maltose 1-phosphate as substrate for the maltosyltransferase GlgE, but detection of this phosphosugar in cell-free culture supernatants has never been reported.

#### 2.2.4 α-Glucans in Biofilm Forming Bacteria

Many bacteria are capable of forming biofilms, which are microbial communities characterized by their adhesion to solid surfaces of biotic and abiotic origin. An essential element in establishment and maintenance of a biofilm is the production of an extracellular matrix of exopolymeric substances (EPS) consisting of polysaccharides, proteins, DNA, and lipids. The EPS surrounds the microorganisms lending structural integrity, mechanical stability, and cohesiveness to the biofilm. The composition of the exopolymeric polysaccharides is diverse and can be complex comprising a mixture of several different types of molecules. Various types of α-glucans have been reported as a minor or major component of exopolymeric polysaccharides for some biofilm-producing bacteria (Table 2).

A group of bacteria notoriously forming α-glucan-containing biofilms are lactic acid bacteria. Synthesis of α-d-glucans by lactic acid bacteria occurs extracellularly using sucrose as a substrate and only requires secretion of single GH70 glucansucrase enzymes, which employ an α-retaining double displacement mechanism. The specificities of these glucansucrases differ, leading to production of various types of α-glucans with diverse linkages of the glucose units as well as the branching pattern. Thus, these extracellular α-glucans also exhibit very different physicochemical properties. Based on the linkage composition, these α-d-glucan polysaccharides are classified into dextran with mainly α(1→6) linkages, mutan with predominate α(1→3) linkages, alternan with alternating α(1→6) and α(1→3) linkages, and reuteran with mainly α(1→4) linkages.^122−125^ In addition, some lactic acid bacteria produce and secrete GH70 branching sucrases that can add single α(1→2) or α(1→4)-branched residue using dextran as an acceptor, resulting in highly branched polysaccharides with comb-like structure.^126^

Dextran is a homopolysaccharide which is composed of d-glucose monomers with mainly consecutive α(1→6) linkages in the backbone and branches connected via α(1→3) and occasionally α(1→2) and α(1→6) linkages (Figure 2A).^127^ The sucrose-dependent GH70 glucansucrase enzymes that synthesize dextran are termed dextransucrases.^128^*Leuconostoc mesenteroides* produces dextran consisting of 95% α(1→6) linkages and 5% α(1→3) branching linkages.^123^ Concerning the length of the branches, differing values have been reported depending on the studied strain of *L. mesenteroides*. 40% of the branching side chains of dextran produced by *L. mesenteroides* strain NRRL B-512 contain only one glucosyl unit, while 45% of the branching side chains possess two glucosyl units, and the remaining are longer than two glucosyl units.^129^ Similarly, branches from dextran obtained from strain *L. mesenteroides* NRRL B-1397 were reported to possess α(1→2) branches comprising just one glucosyl unit and longer α(1→3) branches comprising on average five glucosyl units,^130^ whereas dextran from *L. mesenteroides* strain NRRL B-512F was demonstrated to contain side chains up to 33 glucose residues.^131^ The molecular weight of dextran produced by dextransucrase enzymes from different bacteria generally varies in the range of 9–500 × 10^6^ Da depending on the producing strains and enzymes. Dextran produced by *L. mesenteroides* exhibits a molecular weight of >2.0 × 10^6^ Da, while that produced by *Weissella cibaria* was described to have a higher-molecular weight of 4 × 10^8^ Da.^128,132^*Oenococcus kitaharae* DSM17330 synthesizes a dextran of over 10^9^ Da, which is the largest dextran reported to date.^133^ Linear dextrans exhibiting exclusively α(1→6) linkages are very flexible polymers that are generally highly soluble in water. The water solubility of different dextrans is modulated by their branching linkage pattern and degree of branching.^134^ The high-molecular weight dextran from *O. kitaharae* DSM17330 was reported to exhibit a gel-like behavior.^133^ Cryo-TEM and dynamic light scattering analysis revealed that the dextran synthesized *in vitro* by the dextransucrase of *L. mesenteroides* strain D9909 displayed well-defined spheroidal particles in solution, with diameters ranging from 100 to 450 nm.^135^

Mutans are water-insoluble α-glucans, mainly consisting of consecutive α(1→3) linkages in the glucan chain backbone but may also contain a minority of consecutive α(1→6) linkages as well as α(1→3) and α(1→6) branches (Figure 2A).^136^ Mutans are generally produced from *Streptococcus* strains by specific sucrose-dependent GH70 glucansucrase enzymes termed mutansucrases and are associated with the development of dental caries.^137^ Structural analysis of a water-insoluble mutan produced by *Streptococcus mutans* strain 6715 revealed the presence of 67% of continuous α(1→3) linkages in the backbone and 33% α(1→6) linkages extending linearly from the branches.^138^*Streptococcus salivarius* strain HHT produced a water-insoluble mutan containing a high proportion of 80% of α(1→3) linkages and short side chains of α(1→4) and α(1→6) linkages.^139^ The molecular weight of mutans produced by *S. mutans* strains has been reported to be in the range of ca. 2.4 × 10^3^ Da.^140^ Scanning electron microscopic examination of the mutan produced by *S. mutans* strain 20381 revealed a fibrillary structure consisting of granular formations.^141^

Reuterans are glucopolysaccharides found in *Lactobacillus reuteri* consisting of alternating consecutive α(1→4) linkages and single α(1→6) linkages in the glucan chain backbone as well as α(1→6) branches. The specific sucrose-dependent GH70 glucansucrase enzymes capable of forming alternating α(1→4) and α(1→6) linkages for synthesis of reuteran are termed reuteransucrases.^142^ Structural analysis of the reuteran produced by the reuteransucrase GtfA of *L. reuteri* strain 121 revealed a composition of 58% α(1→4) and 42% α(1→6) linkages, with a molecular weight of 34.6 × 10^6^ Da.^143,144^ It is built up from maltose, maltotriose, and maltotetraose building blocks connected by single α(1→6) linkages, with some of the α(1→4) linked building blocks carrying α(1→6) branches.^143^ The reuteran polysaccharide produced by the reuteransucrase GtfO of *L. reuteri* strain ATCC 55730 is composed of 80% α(1→4) linkages and 20% α(1→6) linkages, suggesting the presence of longer stretches with consecutive α(1→4) linkages instead of alternating α(1→4) and α(1→6) linkages.^128^ Similarly, the exopolysaccharide produced by *L. reuteri* strain SK24.003 possesses predominantly α(1→4) linkages (80%) and a lower degree of α(1→6) linkages with a molecular weight of 43.1 × 10^6^ Da and a radius of gyration of 43.6 nm.^145^ Structural modelling of reuteran from *L. reuteri* strain SK24.003 revealed that its alternating α(1→4)/α(1→6) backbone and branches are packed into a helical groove, generating a helical conformation in solution.^146^

Alternan is a high-molecular weight α-d-glucan homopolymer containing alternating α(1→6) and α(1→3) linkages (Figure 2A). The production of alternan has mainly been reported for strains of *L. mesenteroides* and *Leuconostoc citreum*. The specific sucrose-dependent GH70 glucansucrase enzymes capable of forming alternating α(1→6) and α(1→3) linkages for synthesis of alternan are termed alternansucrases.^147^ Alternan is a branched α-d-glucan with 7–11% 3,6-sustituted-d-glucosyl residues.^148^*L. citreum* strain ABK-1 encodes an alternansucrase that catalyzes the synthesis of an α-d-glucan with 60% α(1→6) linkages and 40% α(1→3) linkages.^149^ The molecular sizes of alternans produced by *L. citreum* strains SK24.002 and L3C1E7 were determined as 46.2 × 10^6^ Da and 5.88 × 10^6^ Da, respectively.^150,151^

In addition to typical glucansucrases, some lactic acid bacteria, particularly strains of *L. citreum*, have been reported to produce and secrete a distinct group of GH70 family enzymes, designated branching sucrases.^126^ Unlike typical glucansucrases, branching sucrases use sucrose as a substrate but do not catalyze α-d-glucan polysaccharide formation. They rather hydrolyze sucrose and transfer the glucosyl moiety to introduce branches into α-glucans produced by regular GH70 glucansucrases of the same bacterial strain.^126,152^ When dextran is provided as an acceptor substrate, branching sucrases catalyze the synthesis of α(1→2) or α(1→3) linked single glucosyl unit branches onto dextran, generating highly branched dextran with a comb-like structure, where up to 50% of the glucosyl monomers of the linear glucan backbone carry branches.^126^ The presence of α(1→2) or α(1→3) branches in branched dextran renders the polymer resistant to the hydrolysis by digestive enzymes of the gastrointestinal tract of mammals.^153–155^

In addition to capsular α-glucans present in *M. tuberculosis* as described above in section 2.2.3, extracellular α-d-glucan homopolymers with a glycogen-like structure comprising an α(1→4) linked core with α(1→6) branches have also been reported for some biofilm-forming bacteria. Species of the genus *Neisseria* express a GH13 amylosucrase, which is a sucrose-dependent α-amylase family enzyme that catalyzes the synthesis of a high-molecular weight linear α(1→4) linked core.^156^ First, this enzyme has been identified in the cytosol in some *Neisseria* species, leading to intracellular glycogen production.^157^ Later, this enzyme was also found to be secreted by *Neisseria polysaccharea* isolated from the throats of healthy children leading to extracellular polymer formation.^158^*In vitro*, in the presence of an activator α-glucan starter molecule (e.g., glycogen), purified amylosucrase protein catalyzes the synthesis of a linear amylose-like polysaccharide composed of only α(1→4) glucosidic linkages using sucrose as the substrate, exhibiting a DP of ∼55.^159,160^ In contrast, the α-glucan isolated from cells of *N. polysaccharea*, *N. perflava*, and others isolated from human dental plaque have been reported to comprise between 6 and 9% α(1→6)-linked branches,^161−164^ indicative of the presence of a branching enzyme acting on the linear α(1→4)-linked polysaccharide produced by GH13 amylosucrases.^165^ While initially thought to be restricted to species of the genus *Neisseria*, more recently GH13 amylosucrases have also been described for bacteria outside this genus such as various *Deinococcus* species, *Arthrobacter chlorophenolicus*, *Alteromonas macleodii*, *Methylobacillus flagellatus*, the cyanobacterium *Synechococcus* sp., and the halotolerant methanotrophic bacterium *Methylomicrobium alcaliphilum*, indicating that this mechanism of producing glycogen-like α-glucan is more widespread.^126^

For members of the genus *Aeromonas*, which are Gram-negative, water-borne bacteria that are ubiquitously found in aquatic environments, a surface α-glucan consisting of α(1→4)-linked glucosyl units with α(1→6)-branches has been described for *A. piscicola* AH-3 and *Aeromonas hydrophila* strains AH-1 and PPD134/91.^166−168^ The α-glucan is produced intracellularly by the UDP-glucose pyrophosphorylase (UGPase) GlgC and the glycogen synthase GlgA. In contrast to the classical GlgC-GlgA pathway for glycogen biosynthesis, *Aeromonas* GlgC synthesizes UDP-glucose instead of ADP-glucose, while *Aeromonas* GlgA can utilize both UDP-glucose and ADP-glucose as substrates.^166−168^ For *A. hydrophila* AH-3, it was demonstrated that the intracellularly produced glycogen-like α-glucan is exported to the cell surface involving WecP,^168^ which is the enzyme catalyzing the transfer of *N*-acetylgalactosamine to undecaprenyl phosphate to initiate O-antigen lipopolysaccharide (LPS) biosynthesis.^169^ The exported glycogen-like α-glucan is attached to the surface involving the O34-antigen polysaccharide ligase WaaL,^168^ which is the enzyme that ligates the O-antigen LPS to the LPS-core.^170^

Presence of extracellular α-glucan consisting of α(1→4)-linked glucosyl units with α(1→6) branches has further been described for the Gram-negative bacterium *Pseudomonas fluorescens*.^171^ In addition, an α(1→4)-linked α-glucan has been identified as part of the EPS produced by the biofilm-forming plant pathogen *Pseudomonas syringae pv. actinidiae* strain NZ V-13.^172^ While α-glucan biosynthesis has not been investigated in *P. fluorescens* and *P. syringae pv. actinidiae* yet, the configuration of pathways leading to α-glucan formation in the related bacterium *Pseudomonas aeruginosa* PAO1 has recently been elucidated, revealing a central role of the GlgE pathway in this organism.^173^ Thus, it is likely that α-glucan formation occurs on a similar pathway in all *Pseudomonads* (Figure 3C). For *P. aeruginosa* PAO1, however, it is unclear whether intracellularly produced α-glucan is secreted to become surface-exposed similar to *P. fluorescens* and *P. syringae pv. actinidiae*.

## 3 α-Glucan Metabolism

### 3.1 The Classical GlgC-GlgA Pathway in Bacteria

The classical bacterial glycogen metabolic pathway involves genes encoding the action of five enzymes. Three enzymes are involved in the anabolic route, (i) AGPase (*glgC*) activating the glucose moiety, (ii) glycogen synthase (*glgA*; GS) generating α(1→4)-linked linear glucose chains, and (iii) glycogen branching enzyme (*glgB*; GBE) introducing α(1→6)-linked glucan branches; while two enzymes are involved in the catabolic route, (i) glycogen debranching enzyme (*glgX*; GDE) cleaving α(1→6)-linked glucan branches, and (ii) glycogen phosphorylase (*glgP*; GP)^61^ (Figure 3A). Genes involved in the classical pathway of glycogen metabolism are often clustered in a single operon.^174−177^ In the case of the paradigmatic *E. coli*, these genes are located in a cluster of 15 kb organized in two neighboring operons.^89,91,178^ The most frequent order for the *glgA*/*B*/*C* triplet is BCA but the order CBA and BAC are also observed. The *glgP* and *glgX* genes are not always present near the glgA/B/C triplet, but if they are, their order is highly variable.^179^ The duplication of genes involved in glycogen metabolism was observed in several bacterial species, likely providing (i) functional redundancy, (ii) different kinetics, substrate specificities and/or expression profiles, and (iii) functional promiscuity/specialization of genes as a source of diversity and evolution of the metabolic pathways.^180,181^ Interestingly, certain bacterial species display additional functions clustered in the *glg* operon, as the *amy* (α-amylase) and *pgm*/*phx* (phosphoglucomutase) genes.^182^

#### 3.1.1 Biosynthesis

Glucose 1-phosphate plays a central role in the metabolism of glycogen (Figure 3A).^183^ Glucose 1-phosphate is produced by the phosphoglucomutase (Pgm) from glucose 6-phosphate. In turn, glucose 6-phosphate is produced either by phosphorylation of extracellular glucose by the hexokinase, or from the final steps of gluconeogenesis by transforming fructose 6-phosphate by the phosphoglucoisomerase.^38^ The activation of glucose 1-phosphate is the first committed and rate-limiting step of the classic glycogen biosynthetic pathway, involving the formation of ADP-glucose mediated by AGPase. AGPase catalyzes a condensation reaction between ATP and glucose 1-phosphate releasing pyrophosphate (PPi) diphosphate and ADP-glucose, requiring Mg^2+^ for activity.^61,184^ AGPase displays a bi–bi mechanism with ATP binding first, followed by glucose 1-phosphate and by the ordered release of PPi and ADP-glucose.^185^ Importantly, AGPase displays positive and negative allosteric regulation.^186−188^ The second step is carried out by GS, which generates linear α(1→4)-linked glucose chains.^61,189^ It is well established that the initiation step of glycogen biosynthesis in yeast and mammals requires the action of the enzyme glycogenin (GN), which is considered the first acceptor of glucose units.^190,191^ GN catalyzes an autoglycosylation reaction, the transfer of a glucose residue from UDP-glucose to a tyrosine residue (Tyr195 in human GN1).^192^ Fully sequenced genomes of bacteria known to accumulate glycogen have failed to reveal the presence of GN homologs.^193^ It was found that GS from *Agrobacterium tumefaciens* can not only elongate α(1→4)-linked glucans but also generate the primer required for the elongation process by catalyzing its own glycosylation.^194^ The oligosaccharides formed by GS were composed of two to nine glucose residues and, in addition, this α-glucan was released from the enzyme.^194^ Thus, it was proposed that bacterial GSs use this *de novo* synthesis mechanism in the absence of available soluble α(1→4)-glucans to provide itself with an initial substrate. It is speculated that GS preferentially catalyzes an elongation reaction of (i) malto-oligosaccharide primers generated during the initiation step or (ii) glycogen, inducing an apparent inhibition of the initiation reaction.^194^ The third step catalyzed by the GBE enzyme produces α(1→6)-linked glucan branches in the polymer. Biochemical and structural data indicate that GBEs only act on long polymers to transfer chains no shorter than six units and preferring chains eight or more sugars in length.^195^

#### 3.1.2 Degradation

The recovery of glucose 1-phosphate from the glycogen degradation pathway is carried out by GP.^196^ GP catalyzes the reversible phosphorolysis of α(1→4)-glucans to obtain glucose 1-phosphate,^197^ requiring pyridoxal phosphate (PLP) as a prosthetic group.^198^ GlgP acts directly on the glycogen polymer, while MalP most likely catabolize soluble malto-oligosaccharides. The majority of characterized polyglucan phosphorylases are unable to act on chains smaller than five glucose residues in length. GlgP is unable to bypass or hydrolyze the α(1→6) linkages and therefore stops two, three, or four residues from the first α(1→6) branch encountered to generate the so-called β-limit dextrin.^199^ Glucose 1-phosphate is subsequently converted to glucose 6-phosphate to enter into the glycolytic pathway providing energy to the cell. Due to the high degree of branching present in the glycogen molecule, a second type of enzyme is required to cleave the α(1→6)-glucosidic bonds that remain uncleaved by GP. Bacterial GDEs display only α(1→6)-glucosidase activity. GDE cleaves the α(1→6)-glucosidic linkage between these glucose residues and the linear α(1→4)-glucan chains of glycogen and relies on MalP and GP to process the remaining α(1→4)-bonds in this short-released chain.

#### 3.1.3 Regulation

The main regulatory step in the bacterial glycogen biosynthetic pathway is carried out by AGPase.^61^ This markedly contrast with the metabolic regulation of glycogen biosynthesis and degradation mechanisms in eukaryotic cells.^109^ AGPase has the ability to sense the energy status of the cell controlling its enzymatic activity by the action of allosteric regulators.^185,187^ AGPase activators are metabolites that reflect signals of high carbon and energy content of a particular bacteria or tissue, whereas inhibitors of the enzyme indicate low metabolic energy levels.^78,200^ Based on the regulatory profiles to different allosteric effector AGPases were classified into nine different classes.^200^

Bacterial AGPases are encoded by a single gene, producing a native homotetrameric protein (α4) with a molecular mass of ca. 200 kDa.^61,186,188^ The paradigmatic bacterial AGPase from *E. coli* (*Ec*AGPase), is positively regulated by glycolytic intermediates, including fructose 1,6-bisphosphate (FBP) as the main activator with pyruvate acting synergistically, and negatively regulated by AMP generated from the general metabolism.^187,200,201^ Covalent binding of pyridoxal resulted in permanent activation of *Ec*AGPase, while the presence of FBP protected the enzyme from binding to the compound, allowing the identification of Lys39 as a key residue in the activation mechanism.^202,203^ The structural and mechanistic aspects of this exquisite allosteric regulation arise from the AGPase tetrameric architecture that will be discussed in Section 5.1.1.1. Interestingly, histidine phosphotransporter protein (HPr), a protein associated with the PTS system and subject to phosphorylation/phosphorolysis modification, interacts with *E. coli* GP (*Ec*GP) regulating its oligomeric status and enzymatic activity.^204,205^ Therefore, this second point of regulation in the classical pathway prevents glycogen synthesis from acting like a futile cycle, tightly controlling the state of the pathway.

Finally, the glycogen metabolism regulation in *E. coli* also involves a complex assemblage of factors that are adjusted to the physiological and energetic status of the cell.^91^ At the level of gene expression, several factors have been described to control bacterial glycogen accumulation, including (i) the PhoP–PhoQ regulatory system,^206^ (ii) the carbohydrate phosphotransferase system (PTS),^207^ (iii) the carbon storage regulator CsrA,^208,209^ and (iv) and the cAMP-CRP responsive inner-membrane nucleoside transporters.^210^

### 3.2 The GlgE Pathway in Bacteria

The GlgC-GlgA pathway has been thought to be the only route for synthesizing intracellular glycogen-like α-glucan consisting of α(1→4)-linked glucosyl units with α(1→6)-branches in bacteria. However, in 2010, the presence of an alternative route that is not relying on a nucleoside diphosphate-activated donor substrate was discovered for glycogen-like α-glucan biosynthesis in mycobacteria, the GlgE pathway.^211,212^ Subsequently, it was found that the GlgE pathway is almost as frequently distributed among bacteria as the GlgC-GlgA pathway, being present in 14% of sequenced bacterial and archaeal genomes, whereas the GlgC-GlgA pathway is found in 20% of bacterial genomes.^213^ The GlgE pathway is very intimately connected and interrelated both in terms of biosynthesis and degradation with trehalose metabolism, as trehalose is a precursor for the formation of the activated donor substrate for GlgE, maltose 1-phosphate, and the glycogen-like α-glucan produced by the GlgE pathway can readily be mobilized to yield trehalose (Figure 3B).

#### 3.2.1 Biosynthesis

The biosynthesis of α-glucans via the GlgE pathway has first and best been studied in actinomycetes and particularly in mycobacteria (Figure 3B). In the GlgE pathway, α(1→6)-branched α(1→4)-glucans are produced by iterative cooperation of two essential enzymes, the maltosyltransferase GlgE (systematic name (1→4)-α-d-glucan:phosphate α-d-maltosyltransferase) and the branching enzyme GlgB.^84,212^ Both enzymes are GH13 family members.^226^ The maltosyltransferase GlgE uses maltose 1-phosphate as the activated donor substrate to produce linear α(1→4)-linked maltooligosaccharides.^212^ As soon as GlgE has formed a linear chain of ∼16 glucosyl residues, GlgB introduces an α(1→6)-branch of ∼7–8 glucosyl residues in length employing a strictly intrachain transfer mechanism. GlgE then preferentially extends the newly formed branch until it is long enough to undergo branching again by GlgB. Only occasionally, GlgE also extends previous branches so that they might become long enough to allow a second branching by GlgB. Therefore, each branched chain mostly carries just one further branch. These specificities of GlgE and GlgB promote an iterative process that results in a glucan polymer that exhibits a significantly lower degree of branching, resulting in a less pronounced arboreal structure compared to glycogen from other bacteria and from mammals.^84^ The branching enzyme GlgB from bacteria employing the GlgE pathway does not substantially differ from that of bacteria using the classical GlgC-GlgA pathway for glycogen formation,^214,215^ indicating that the evolution of the maltosyltransferase GlgE was key for the establishment of the GlgE pathway. Consistent with the strictly iterative process of synthesis of a glycogen-like polysaccharide via the GlgE pathway, the genes encoding GlgE and GlgB are co-transcribed as part of an operon in mycobacteria and all other bacteria possessing the GlgE pathway.^213^ However, while protein structures of GlgE^216−221^ and GlgB^215^ have individually been resolved from different organisms possessing the GlgE pathway, it is unknown whether direct physical interaction between both proteins occurs to mediate the iterative synthesis of α(1→6)-branched α(1→4)-glucans. GlgE from actinomycetes has been shown to form a homodimer and to catalyze the α-retaining transfer of maltosyl units from α-maltose 1-phosphate to maltooligosaccharides using a double-displacement mechanism, i.e., a ping-pong mechanism involving the release of the glucan extended by a maltosyl unit prior to the next reaction.^212,221^*In vitro* experiments using only purified GlgE and GlgB proteins recombinantly expressed from *M. tuberculosis* as well as maltose 1-phosphate as the donor substrate resulted in the formation of high-molecular-weight α(1→6)-branched α(1→4)-glucan particles resembling in chemical structure and supramolecular architecture those natively isolated from cells of *M. tuberculosis* and *Streptomyces venezuelae*,^84^ indicating that GlgE can initiate *de novo* α-glucan synthesis without the need of a primer, similar to the priming function of GlgA activity in the GlgC-GlgA pathway.^194^ In absence of maltose 1-phosphate substrate and presence of maltooligosaccharides, GlgE can also mediate disproportionation by transfer of maltosyl units from the non-reducing end of a donor molecule to the non-reducing end of an acceptor maltooligosaccharide.^212^ In addition to GlgE from actinomycetes, the maltosyltransferase has also biochemically been studied in great detail in recombinantly expressed form from *P. aeruginosa* strain PAO1^173^ (Figure 3C) and *Estrella lausannensis*(222) revealing very similar enzymatic characteristics of the GlgE proteins of different origin.

#### 3.2.2 Formation of the Maltose 1-Phosphate Donor Substrate

In contrast to glucosyltransferases such as the glycogen synthase GlgA that uses nucleoside diphosphate-coupled glucosyl units such as UDP-glucose or ADP-glucose as activated donor substrate, GlgE employs maltose 1-phosphate as activated donor substrate. Two alternative routes for biosynthesis of maltose 1-phosphate have been described, the GlgC-GlgM and the TreS-Pep2 (named TreS-Mak in some organisms) pathways. In actinomycetes such as *M. tuberculosis*, both pathways operate simultaneously, while other bacteria possessing the GlgE pathway only employ the TreS-Pep2 / TreS-Mak route (Figure 3B,C).

Both routes are closely connected to trehalose metabolism. Trehalose is an α,α(1→1)-linked glucose dimer that is abundant in many pro- and eukaryotic organisms. Trehalose is not representing an α-glucan *sensu stricto* and plays a broad variety of biological functions dependent on the producing organism, most of them being unrelated to α-glucan metabolism as has already extensively been reviewed.^223^ However, due to its important connection to the GlgE pathway, trehalose biosynthesis pathways will briefly be described here. Bacteria possessing the GlgE pathway for α-glucan production employ one or both of two alternative routes for *de novo* synthesis of trehalose. The most widespread route in prokaryotes is the OtsA-OtsB pathway. The trehalose 6-phosphate synthase OtsA (a Leloir-type glycosyltransferase) catalyzes the transfer of nucleoside diphosphate-activated glucose to glucose 6-phosphate to yield trehalose 6-phosphate with release of nucleoside diphosphate. In most cases, OtsA uses UDP-glucose. However, OtsA of *M. tuberculosis* (Rv3490) has been demonstrated to exhibit a 10-fold higher affinity for ADP-glucose than for UDP-glucose.^224^ The trehalose 6-phosphate phosphatase OtsB then catalyzes the dephosphorylation of trehalose 6-phosphate to release free trehalose and inorganic phosphate. The *M. tuberculosis* genome encodes two genes with homology to bacterial trehalose 6-phosphate phosphatases, *otsB1* (Rv2006) and *otsB2* (Rv3372). The encoded OtsB1 protein is much larger than OtsB2 (1327 aa vs. 391 aa, respectively). However, only the essential OtsB2 expresses trehalose 6-phosphate phosphatase activity and is relevant for trehalose biosynthesis^225^ while a Δ*otsB1* mutant of *M. tuberculosis* showed no obvious phenotype so that the function of OtsB1 is yet unknown.^226^ In the alternative route, the TreY-TreZ pathway, the maltooligosyltrehalose synthase TreY converts the terminal α(1→4)-glycosidic linkage at the reducing end of a linear α(1→4)-glucan into an α-1,1-bond yielding maltooligosyltrehalose. Maltooligosyltrehalose trehalohydrolase TreZ then hydrolytically liberates trehalose. As this pathway requires linear glucans, branched α-glucans first need to be processed by glycogen phosphorylase GlgP, which reduces the branch length by releasing glucose 1-phosphate, followed by debranching enzyme TreX, which hydrolyzes the α(1→6)-glycosidic branch linkages. TreX, TreY, and TreZ all belong to the GH13 protein family^227^ (Figure 3).

In the most common route for maltose 1-phosphate biosynthesis, the TreS-Pep2/TreS-Mak pathway, the trehalose synthase TreS mediates the conversion of trehalose to maltose by converting the α(1→1)-bond into an α(1→4)-glycosidic bond. Subsequently, maltose is rapidly and quantitatively phosphorylated in an ATP-dependent reaction to maltose 1-phosphate by the maltose kinase (systematic name ATP:α-maltose 1-phosphotransferase), which is named Pep2 or Mak depending on the organism.^228,229^ Previously, TreS was thought to exclusively mediate trehalose formation from maltose. However, although the equilibrium of purified TreS favors the formation of trehalose from maltose *in vitro*, flux through TreS *in vivo* is in the opposite direction whenever a maltose kinase is expressed,^230^ driven by the rapid and irreversible ATP-dependent phosphorylation of the formed maltose to maltose 1-phosphate by the maltose kinase.^216,229^ The observed finding of the direction of flux through TreS for consumption of trehalose is supported by the fact that TreS and Pep2/Mak are expressed as a fusion protein in many organisms with the exception of actinobacteria (i.e., mycobacteria and *Streptomycetes*).^213^ Recombinant bifunctional TreS-Mak from *Estrella lausannensis* has been enzymatically characterized and was found to exhibit an apparent molecular weight of 256 kDa. The TreS-Mak fusion protein produced maltose 1-phosphate in the presence of nucleoside triphosphates and trehalose concentration resembling physiological intracellular conditions.^222^ Likewise, the TreS-Pep2 fusion protein from *P. aeruginosa* strain PAO1 has been reported to exhibit similar enzymatic properties and to synthesize maltose 1-phosphate from trehalose and ATP, although the supramolecular architecture has not been studied.^173^ In contrast, TreS and Pep2 are expressed as individual proteins in actinobacteria. Here, they form a hetero-octameric complex composed of four subunits of TreS and four subunits of Pep2 with an apparent molecular weight of ca. 490 kDa, in which a homotetramer of TreS forms a platform to recruit dimers of Pep2 via a specific interaction domain as observed in *Mycolicibacterium smegmatis*.^231,232^

More recently, a second route for formation of maltose 1-phosphate was discovered in *M. tuberculosis*, the GlgC-GlgM pathway^119^ (Figure 3B). While *M. tuberculosis* GlgM (Rv1212c) was previously believed to be a glycogen synthase and named GlgA accordingly, it was shown that GlgM is a maltose 1-phosphate-producing glucosyltransferase (formal name ADP-α-d-glucose:α-d-glucose-1-phosphate 4-α-d-glucosyltransferase) three orders of magnitude more efficient at transferring glucose from ADP-glucose to glucose 1-phosphate than to glycogen.^119^ Recently, GlgM from the related bacterium *M. smegmatis* has been crystallized, and enzymatically characterized revealing very similar properties to the enzyme from *M. tuberculosis*.^233^ GlgM from mycobacteria is a GT4 family enzyme employing an α-retaining mechanism, while *bona fide* bacterial glycogen synthases are GT5 family members. Bioinformatic analysis of bacterial genomes revealed that ∼32% of all annotated GlgA homologues exhibit GT4 membership. It is striking that in every case where GlgA and GlgE coexist in Gram-positive bacteria (typically actinomycetes including mycobacteria and streptomycetes), the GlgA belongs exclusively to the GT4 family and never to the GT5 family. This coexistence strongly suggests that all these GlgA homologues actually have no glycogen synthase activity but rather represent maltose 1-phosphate-producing ADP-α-d-glucose:α-d-glucose 1-phosphate 4-α-d-glucosyltransferases and should be named GlgM accordingly.^119^ This finding also implies that the proportion of microbes that possess the classical GlgC-GlgA glycogen pathway is only ∼20% and thus lower than previously estimated.^213^ GlgM prefers ADP-glucose as the donor substrate and can use UDP-glucose at ca. 10-fold lower efficiency. Thus, GlgM is essentially linked to GlgC for the production of ADP-glucose. Similarly, the affinity of trehalose 6-phopshate synthase OtsA from *M. tuberculosis* for ADP-glucose was found to be one order of magnitude higher than for UDP-glucose.^224^ Mutational studies in *M. smegmatis* revealed that GlgM and OtsA are the main consumers of ADP-glucose in this organism as revealed by substantial intracellular ADP-accumulation in the *M. smegmatis* Δ*glgA*(u) Δ*otsA* double mutant. Furthermore, it was found that there is a redirection of the flux of ADP-glucose since neither the *M. smegmatis* Δ*glgA* nor the Δ*otsA* single mutants accumulated detectable amounts of ADP-glucose. When GlgM is inactivated, ADP-glucose is redirected through OtsA promoting trehalose formation, whereas the increased trehalose level in turn stimulates maltose 1-phosphate synthesis via TreS-Pep2.^119^ Thus, the TreS-Pep2 and GlgC-GlgM pathways for maltose 1-phosphate synthesis are linked via the shared use of the intermediate ADP-glucose by GlgM and OtsA. The flux of ADP-glucose seems to be sufficiently redirected such that the net rate of maltose 1-phosphate generation and α-glucan accumulation can be balanced to some extent when one of the two routes is perturbed.^119^

#### 3.2.3 Degradation

The structure of glycogen-like α-glucan produced by the GlgE pathway is virtually indistinguishable from those produced by the classical GlgC-GlgA pathway. Therefore, the GlgE pathway does not require a special set of enzymes for the degradation of the produced polysaccharide but relies on the same machinery as the GlgC-GlgA pathway. In fact, one set of degradative enzymes is also present in organisms such as *P. aeruginosa* PAO1 where both pathways have been reported to operate in parallel.^173^ The α(1→6)-branched α(1→4)-glucans produced by either pathway can be principally degraded to glucose 1-phosphate for energy production or to feed primary metabolism, or to trehalose (Figure 3). For this, glycogen phosphorylase GlgP reduces the branch length by releasing glucose 1-phosphate from the non-reducing ends of α-glucan chains until the branch length reaches DP4, where GlgP is unable to further degrade external chains due to steric constraints in the vicinity of branch points.^199^ The glycogen debranching enzyme TreX (named GlgX in other organisms) hydrolyzes the α(1→6) links in branched α-glucan. The enzyme has been shown to have a preference for short branch lengths of DP4 such as those produced by GlgP. Thus, TreX/GlgX is not active on glycogen with full-length branches, thereby preventing a futile cycle.^234,235^ Consequently, complete degradation of glycogen requires a close cooperation between GlgP and TreX/GlgX. Linear α(1→4) linked maltooligosaccharides produced by GlgP and TreX/GlgX can either be completely degraded to glucose 1-phosphate by GlgP or converted to trehalose by the TreY-TreZ pathway, where the maltooligosyltrehalose synthase TreY converts the terminal α(1→4)-glycosidic linkage at the reducing end into an α(1→1)-bond yielding maltooligosyltrehalose followed by release of free trehalose by the maltooligosyltrehalose trehalohydrolase TreZ. Although the OtsA-OtsB2 pathway dominates for trehalose synthesis *in vitro* and *in vivo* in a mouse infection model, the importance of glycogen-like α-glucan as a source for trehalose *in vivo* has been demonstrated for *M. tuberculosis* and *M. smegmatis*, for which trehalose is an essential metabolite.^225,230^ Not only does the combined inactivation of the *otsA* and *treY*-*treZ* genes result in an auxotrophic mutant requiring supplementation with exogenous trehalose but also blocking glycogen synthesis in combination with loss of *otsA*, i.e., in Δ*otsA* Δ*glgC* or Δ*otsA* Δ*glgM* double mutants.^119,225^ In *P. aeruginosa* strain PAO1, the α-glucanotransferase MalQ is a maltooligosaccharide disproportionating enzyme that is capable of producing maltose when maltotriose is provided as a donor molecule, which will be generated during disproportionation of maltohexaose as a substrate. Thus, when coupled to Pep2 activity and ATP as a substrate as a virtually irreversible sink, it was shown that MalQ is sufficient to produce maltose 1-phosphate from maltohexaose. Thereby, MalQ can bypass the TreY-TreZ-TreS reactions to feed the GlgE pathway with maltose from maltooligosaccharides in this organism.^173^ In summary, both the biosynthesis and degradation of glycogen-like α-glucan produced by the GlgE pathway are intimately interrelated with trehalose metabolism since trehalose can be readily converted to α-glucan via TreS-Pep2/Mak and GlgE-GlgB and recycled by GlgP-TreX/GlgX and TreY-TreZ.^213^

#### 3.2.4 Regulation

The regulation of the GlgE pathway regarding both the biosynthesis and mobilization of the glycogen-like α-glucan has very sparsely been studied. As mentioned above, due to the fact that biosynthesis and degradation occur in the same cellular compartment and the close link with trehalose metabolism, tight regulation is required to avoid futile cycles of parallel synthesis and mobilization. However, how this is mediated is largely unknown.

A key enzyme of the GlgE pathway is the essential maltosyltransferase GlgE. It was reported that enzymatic activity of GlgE from *M. tuberculosis* and other actinomycetes is negatively regulated *in vitro* and *in vivo* through phosphorylation by the serine/threonine protein kinase PknB. PknB specifically phosphorylates one serine (Ser-85) and six threonine (Thr-10, Thr-59, Thr-148, Thr-191, Thr-193, and Thr-370) residues.^236^ Negative regulation of GlgE by protein phosphorylation is a mechanism distinct from the regulation of the classical GlgC-GlgA-dependent glycogen biosynthetic pathway that involves allosteric regulation by metabolic intermediates.^200^ However, it is still unclear how phosphorylation of GlgE by PknB is regulated and whether this negative regulation of enzymatic activity can be reverted by dephosphorylation. A temperature-sensitive GlgE mutant of *M. smegmatis* was found to be rescued by overexpression of the *garA* gene, which is a forkhead-associated (FHA) domain protein that is a target of PknB itself.^237^ Since FHA domain proteins have been described to be modules that bind to phospho-Thr residues in signaling cascades, it was conceivable that GarA might directly interact with phosphorylated GlgE. However, no direct interaction of the GarA protein with GlgE neither in phosphorylated nor non-phosphorylated form could be detected. Most likely, GarA in artificially high concentration can act as decoy for PknB-mediated GlgE phosphorylation and plays no role in regulation of the GlgE pathway *in vivo*.^238^ Genes of the GlgE pathway from *M. tuberculosis* such as *glgE*, *glgB*, and *treS* have been shown to be upregulated under conditions mimicking lysosomal stress conditions implying increased production of glycogen-like α-glucans.^239^ Furthermore, increased capsular α-glucan levels have been reported for *M. tuberculosis* under phosphate-limiting conditions.^240^ However, for both cases, the underlying signaling mechanism has not been elucidated, and it is also unknown if and how increased production of glycogen-like α-glucans might provide a specific advantage under these stress conditions. In yet another study, blocking GlgE enzymatic activity and maltose 1-phosphate accumulation in *M. tuberculosis* has been reported to elicit a pleiotropic stress response that included the upregulation of the genes encoding the TreX-TreY-TreZ branch for trehalose accumulation.^212^ Theoretically, this could mean the existence of a feedback-loop that can sense reduced cellular α-glucan levels and responds to it by promoting maltose 1-phopshate production via the TreS-Pep2/Mak branch from trehalose. However, since trehalose also functions a compatible solute and general small-molecule stress protectant in many bacteria,^241^ the upregulation of trehalose biosynthesis in maltose 1-phosphate-stressed *M. tuberculosis* cells might simply represent a misled general stress response rather than a specific regulatory mechanism involved in α-glucan metabolism.^212^

*M. tuberculosis* has been shown to engage two alternative routes for providing the maltose 1-phosphate building block of the GlgE pathway (Figure 3B). However, the TreS-Pep2 and GlgC-GlgM pathways do not equally contribute to maltose 1-phosphate production and are obvious subject to regulation. The TreS-Pep2 pathway dominates in culture while the GlgC-GlgM-dependent route is more important during infection. The molecular basis underlying these regulatory mechanisms under different physiological conditions remains to be fully elucidated.^119^ In mycobacteria, there is an apparent rechanneling of ADP-glucose between the TreS-Pep2/Mak and the GlgC-GlgM pathways such that the net production maltose 1-phosphate can largely be compensated for when one of these pathways is perturbed. The accumulation of ADP-glucose was detected only in a *M. smegmatis* Δ*glgM* Δ*otsA* double mutant, while none was detected in the Δ*glgM* and Δ*otsA* single mutants. These findings are consistent with the flux of ADP-glucose being redirected through OtsA when GlgM is inactive, leading to an enhanced generation of trehalose and subsequent conversion to maltose 1-phosphate via the TreS-Pep2/Mak pathway.^119^

Similar to regulation of glycogen biosynthesis in bacteria employing the classical GlgC-GlgA pathway, regulation of the GlgE pathway might also take place by allosteric regulation of enzymes controlling metabolic flux of intermediates. For the classical GlgC-GlgA pathway, regulation occurs at the level of the AGPase GlgC, which is typically activated by metabolites of glycolytic pathways such as fructose 6-phosphate, fructose 1,6-bisphosphate, or pyruvate, and inhibited by AMP, ADP, and/or Pi.^200^ A very similar allosteric regulation might occur at the stage of GlgC for those bacteria possessing the GlgC-GlgM route for maltose 1-phosphate synthesis. For example, it has been shown that purified AGPase GlgC from *M. tuberculosis* was allosterically activated primarily by phosphoenolpyruvate and glucose 6-phosphate, while the enzyme from *Streptomyces coelicolor* exhibited sensitivity to allosteric regulation by mannose 6-phosphate, phosphoenolpyruvate, fructose 6-phosphate, and glucose 6-phosphate, whereas NADPH was a main inhibitor.^242^ Furthermore, the other route for maltose 1-phosphate synthesis from trehalose via the TreS-Pep2/Mak pathway might be subject to indirect allosteric regulation since the activity of trehalose 6-phosphate synthase OtsA from *M. tuberculosis* involved in providing the trehalose substrate for TreS-Pep2/Mak has been described to be increased by fructose 6-phosphate.^224^

### 3.3 The Sucrose to α-Glucan Pathway

**Figure 4:**
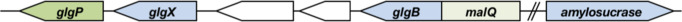
Arrangement of glycogen metabolizing genes in *Neisseria sicca*. Colors indicate the family members according to the CAZy classification: *glgP*, glycogen phosphorylase (GT35 family: dark green background); *glgX*, glycogen debranching enzyme; *glgB*, glycogen branching enzyme and amylosucrase (GH13 family: blue background); *malQ*, α(1→4)-glucanotransferase (GH77 family: light green background). Arrows indicate transcriptional orientation. Parallel black lines represent a physical separation between the genes. The white arrows represent unrelated glycogen genes that encode a subunit of the HlyD family efflux transporter (WP_003761172.1) and a hypothetical protein (WP_003761174.1). *glgP*; glycogen phosphorylase; *glgX*, glycogen debranching enzyme; *glgB*, glycogen branching enzyme; *malQ*, α(1→4)-glucanotransferase.

The sucrose-dependent glycogen pathway is unique among biosynthetic pathways discussed so far. Indeed, the glycogen is synthesized directly from sucrose through the combined actions of amylosucrase and branching enzymes without using an activated nucleotide-sugar or a phosphorylated sugar as building blocks.^243^ Initially found in the culture of *Neisseria perflava*,^244^ the majority of the amylosucrase studies have been conducted in *N. polysaccharea*. The genus *Neisseria* consists primarily of commensal Gram-negative cocci and two pathogenic species: *Neisseria meningitidis* and *Neisseria gonorrhoeae*. The term amylosucrase was coined by Herhe to describe a novel enzyme capable of synthesizing α-polysaccharides from sucrose.^244^ Early studies suggested, like other sucrose-utilizing enzymes such as dextransucrase, that amylosucrase can synthesize both α(1→4) and α(1→6) *O*-glucosidic bonds.^243,245^ Nonetheless, recombinant amylosucrases of *N. polysaccharea* and *Deinococcus radiodurans* formed linear α(1→4) glucan chains with no detectable α(1→6) linkage from sucrose.^159^ Two years later, Büttcher and coworkers characterized the branching enzyme of *Neisseria denitrificans* and demonstrated the stimulation of branching enzyme activity on amylosucrase activity.^165^ The branching enzyme, in fact, causes an exponential rise in the number of non-reducing ends. Each new extremity becomes a potential acceptor for the covalently attached glucosyl residue to amylosucrase. Interestingly, in a survey of the arrangement of glycogen genes in *Neisseria suica* (Figure 4), both *glgB* and *malQ* genes are fused to produce a large protein with a branching enzyme domain at the C-terminus and an α(1→4) glucanotransferase domain at the N-terminus. Currently, the amylosucrase (EC 2.4.1.4) is a member of the extensive GH13 α-amylase superfamily that establishes a transient covalent linkage with the glucosyl residue from sucrose and releases fructose.^246^

**Figure 5:**
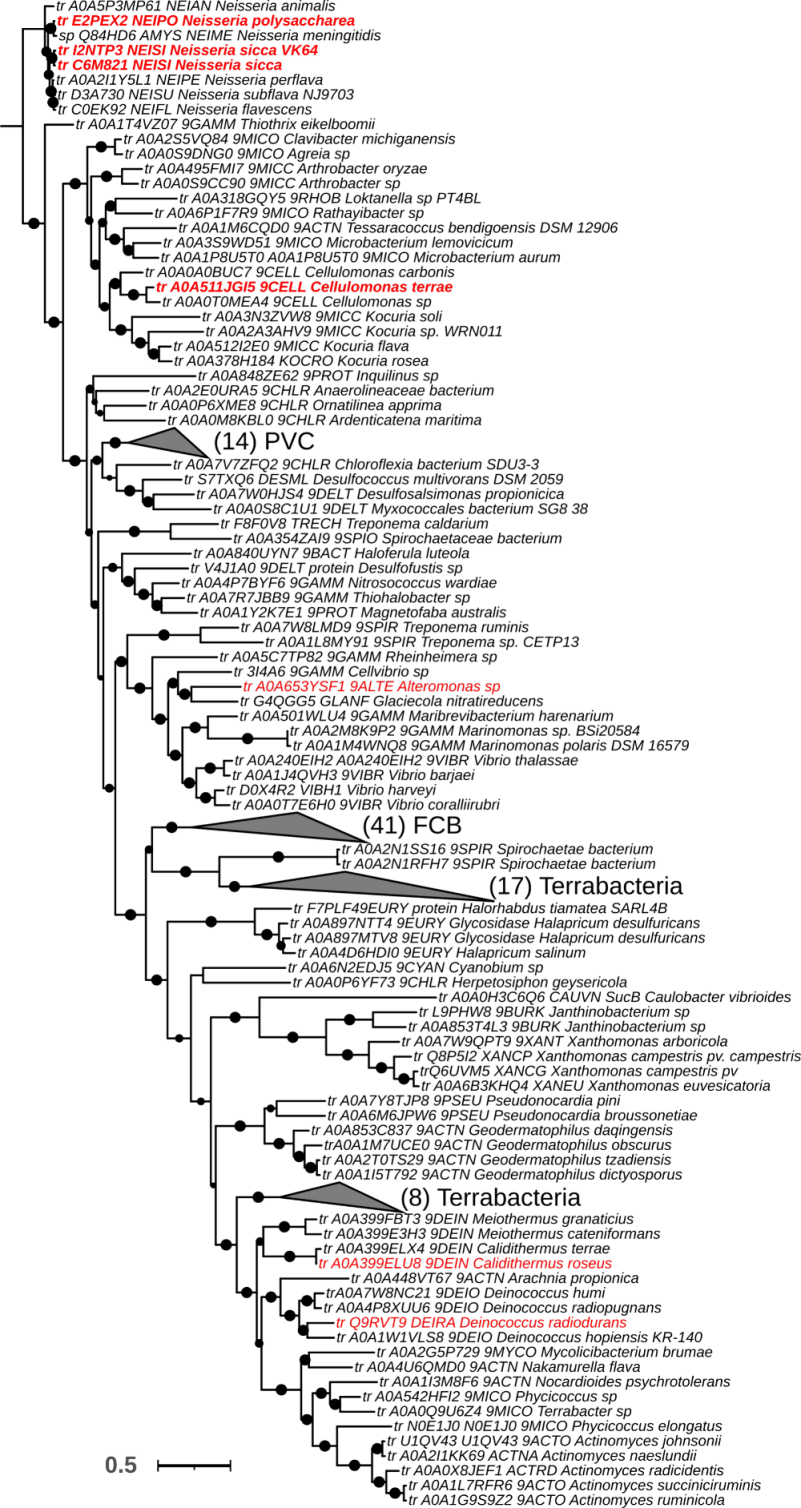
Maximum likelihood of putative-amylosucrases using substitution model LG4X implemented in IQTREE. The selected sequences were retrieved from the NCBI database and then aligned and then trimmed using MAFFT (v7.450) and TrimAl (V1.3) implemented in the webserver Phylemon 2 (http://phylemon2.bioinfo.cipf.es/), respectively. Black circles symbolize ultrafast bootstrap support values greater than or equal to 70. The scale bar indicates the number of substitutions per site. PVC stands for Planctomycetes, Verrumicrobia Chlamydiae. FCB stands for Fibrobacter Chlorobi Bacteroides. The amylosucrase in red indicate that they have been characterized enzymatically.

Initially described in the *Neisseria* genus, the expanding number of complete bacterial genomes revealed a widespread distribution of putative amylosucrase sequences across prokaryotes. As depicted in Figure 5, the phylogenetic tree was inferred with putative-amylosucrases identified in various bacteria classes, including the large *Terrabacteria* group [*D. radiodurans*,^247^*Arthrobacter*,^248^*Synechococcus* PCC7002,^249^*Cellulomonas*,^250^*Calidithermus timidus*,^251^*Truepera*^252^]; γ-proteobacteria [*Alteromonas macleodii*^253^]; β-proteobacteria [*Methylobacillus*^254^]; and α-proteobacteria [*Methylomicrobium alcaliphilum*^255^].

Few reports have been published on the physiological function of amylosucrase in prokaryotes. In *N. polysaccharea*, the amylosucrase contributes to intracellular glycogen synthesis to compensate for the lack of glycogen synthase activity and the synthesis of linear polysaccharides outside the cell. Amylosucrase has recently been hypothesized to be involved in the cyanobacterium’s sucrose metabolism pathway, *Synechococcus* PCC7002.^249^ This assumption is based upon the fact that amylosucrase and sucrose-related genes are arranged in an operon (*spsA*-*sppA*-*frkA*-*amsA*: sucrose synthase, sucrose phosphate phosphatase, fructokinase, and amylosucrase). Sucrose biosynthesis is an osmotic protectant tightly regulated in response to environmental changes. Therefore, sucrose homeostasis is maintained by a balance of synthesis and degradation, either with glycogen serving as a buffer or through the invertase activity (EC3.2.1.26) that hydrolyzes sucrose into glucose and fructose. In terms of energy consumption, hexokinase and fructokinase will consume one ATP each for recycling both glucides. Interestingly, transferring the glucosyl residue of sucrose onto the glycogen pool decreases ATP consumption by 50% as well as the osmotic pressure, avoiding the glucogenesis route. This alternate pathway provides a significant advantage to bacteria thriving in a poor carbon environment.

Over the past years, amylosucrases have attracted more attention due to their capability of isomerization and transglycosylation. To date, many efforts are pursued to engineer amylosucrase enzyme with better thermal stability properties,^256,257^ higher yield of sweeteners production through isomerization (turanose: glucose α(1→3)-fructose), trehalulose (glucose α(1→1)-fructose), and to improve their ability to glycosylate unnatural acceptors.^258^

### 3.4 Archaeal Glycogen: The Origins

In recent years, metagenome-assembled genomes have considerably increased our understanding of microbial diversity on Earth, particularly in the kingdom of Archaea, the third branch of the tree of life. According to collected samples, the population of Archaea and their phylogenetic topology are continuously expanding and changing. The Archaea kingdom is now constituted of the Euryarchaeota, the TACK group (Thaumarchaeota, Aigarchaeota, Crenarchaeota, and Korarchaeota), the DPANN (Daipherotrites, Parvarchaeota, Aenigmarchaeota, Nanoarchaeota Nanohaloarchaeota), and more recently the Asgard superphylum, which comprises Lokiarchaeota, Odinarchaeota, Thorarchaeota, and Heimdallarchaeota phyla.^259^ Remarkably, these archaea share unexpected similarities with eukaryotic cells.^260^ This discovery gives us new insights into the origin of eukaryotic cells. Despite intense debates, a growing body of phylogenomic evidence supports the idea that the Eukaryota phylum emerged from a proto-eukaryotic cell related to the archaeal Asgard superphylum, suggesting a two-domain rather than a three-domain tree of life (). In Archaea, the glycogen metabolism pathway and its regulation are mainly unexplored. The first evidence of glycogen in Archaea came from the pioneering work of König in 1982. König and colleagues observed and biochemically characterized the glycogen accumulation in numerous archaea, including the thermoacidophilic archaea *Sulfolobus acidocaldarius*, *Thermoproteus tenax*, *Desulfurococcus mucosus*, and *Desulfurococcus mobilis* (TACK group), and *Thermoplasma acidophilum* (Euryarchaeota).^261^

Exploration of the glycogen metabolism route in Archaea is currently facilitated by the use of annotated databases and blast search, notwithstanding the necessity of this laborious purifying and characterization effort for confirming glycogen synthesis. In this Review, putative glycogen-metabolizing enzymes were retrieved from the UniProt and CAZy databases for each representative Archaea phylum. Table 3 summarizes the glycogen gene content and the number of isoforms. Several members of the TACK, Asgard, Euryarchaeota, and DPANN superphyla lack all genes related to carbohydrate metabolism, as seen in this table. It is worth noting that other molecules, such as polyphosphate granules, polyhydroxyalkanoates, and triacylglycerol, have been found as potential energy storage alternatives.^262^ Moreover, several studies demonstrate the importance of syntrophic relationships among microbial communities. A recent analysis of the interaction between *Candidatus* Nanohalobium constans and *Halomicrobium*, two halophilic archaea, provides an outstanding example.^263^

**Table 3:**
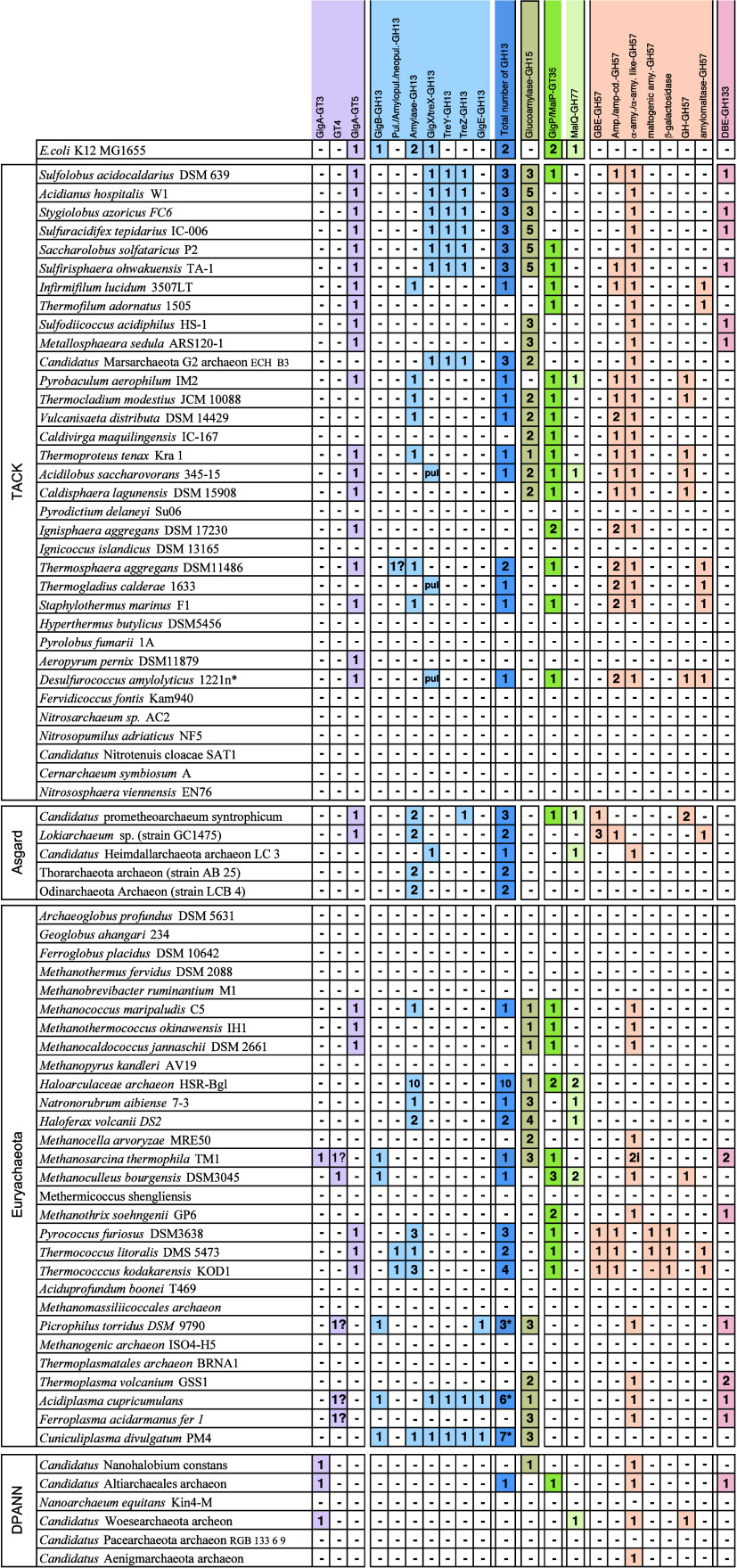
Archaea Glycogen-Metabolizing Enzymes Content^a^

a Representatives of each archaeal phylum were chosen based on their availability in the CAZy or Uniprot databases. TACK group and DPANN are superphyla that include several phyla whose first letters form the acronyms: Thaumarchaeota, Aigarchaeota, Crenarchaeota, and Korarchaeota (TACK) and Daipherotrites, Parvarchaeota, Aenigmarchaeota, and Nanoarchaeota Nanohaloarchaeota (DPANN). The glycogen-metabolizing enzymes and their isoform numbers were classified. For the sake of clarity, background colors were employed. For instance, enzymes belonging to the GH13 family have a blue background. The number associated with GT or GH represents the family. GlgA-GT3 and GlgA-GT5: glycogen synthase activities; GT4: glycosyltrasferase; GlgB-GH13: branching enzyme; Pul./Amylopul./neopul.-GH13: pullulanase/amylopullulanase/neopullulanase; GlgX/TreX: debranching enzyme; TreY-GH13: maltooligosyl trehalose synthase; TreZ: maltooligosyl trehalose hydrolase; GT35-GlgP/MalP are acronyms for glycogen phosphosphorylase and maltodextrin phosphorylase; GBE-GH57: glycogen branching enzyme; Amp./amp-cd.-GH57 amylopullulanse/amylopullulanase cyclodextrinase; DBE-GH133: amylo-α(1→6) glucosidase. Question marks indicate that the function of GT4 in glycogen metabolism requires clarification. In the column GH13 total number, an asterisk denotes the presence of trehalose synthase (TreS). The letter “i” indicates the presence of one inactive isoform.

#### 3.4.1 The Formation of α(1→4) Bonds in Archaea

Like prokaryotes and eukaryotes, the synthesis of α(1→4)-glucan chains relies mainly on the nucleotide-sugar-dependent glycogen pathway. In contrast, only a few archaea belonging to the superphylum Euryarchaeota (Picrophilus torridus, Acidiplasma cupricumulans, Cuniculiplasma divulgatum) utilize the alternate glycogen route, known as the GlgE pathway (Table 3). Two families, GT3 and GT5, of the 115 GT families reported in the CAZy Database catalyze the transfer of the glucose moiety of NDP-glucose onto the non-reducing ends of growing α(1→4)-glucan chains. GlgA-GT3 and GlgA-GT5 differ in their preference for the nucleotide-sugar donor and in their capacity to initiate *de novo* glucan chain synthesis. In contrast to ADP-glucose-dependent GlgA-GT5, heterotrophic eukaryotes GS-GT3 utilizes UDP-glucose and requires a primer, i.e., short glucan, to initiate glycogen synthesis. A GT belonging to family 8, termed glycogenin, specifically synthesizes the primer, composed of 12 to 15 glucose residues, by successive self-glycosylation reactions. Both GlgA-GT5 and GlgA-GT3 are represented among Archaea according to the CAZy database. GlgA-GT5 glycogen synthases are widely distributed in the TACK group and to a lesser extent in Euryarchaeota, whereas GlgA-GT3 glycogen synthases are confined to the DPANN group and Euryarchaeota.

Archaeal GlgA-GT5 have been characterized biochemically in *Sulfolobus acidocaldarius*, *Thermococcus hydrothermalis*, and *Pyrococcus furiosus*.^261,264,265^ These studies demonstrate that archaeal GlgA-GT5 utilizes either ADP-glucose or UDP-glucose as a nucleotide sugar, and other nucleotide diphosphate glucose derivatives such as GDP-glucose for *Pyrococcus furiosus* glycogen synthase.^265^ Intriguingly, Sueda and colleagues have recently proven that the NDP-sugar pyrophosphorylase gene of *Haloarcula japonica* encodes a GDP-glucose pyrophosphorylase (GGPase) activity and not an ADP- or UGPase, as was expected.^266^ Because the GlgA-GT5 has not been characterized enzymatically, the selectivity of glycogen synthase toward GDP-glucose is unknown.

**Figure 6:**
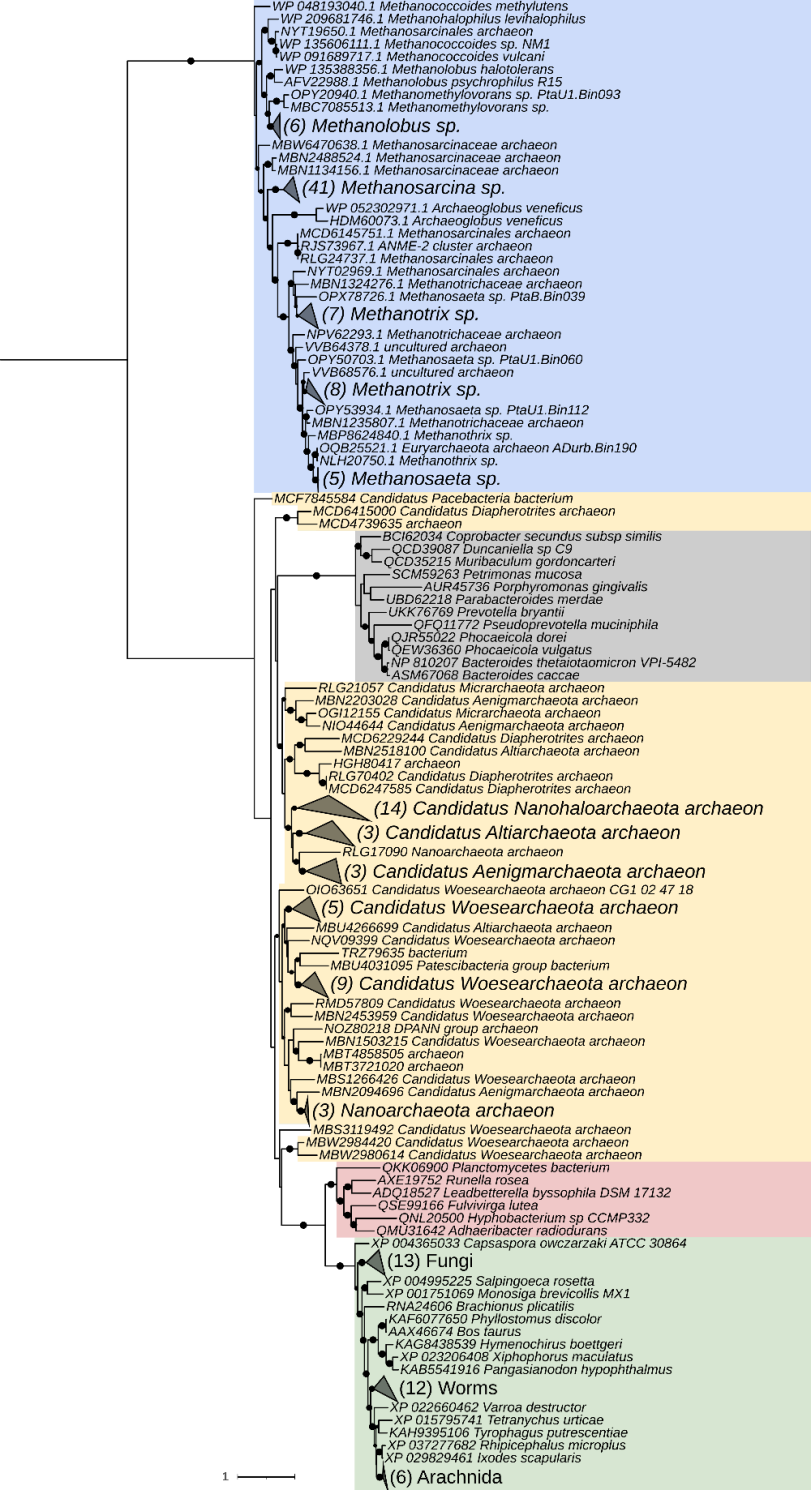
Maximum likelihood phylogeny of glycogen synthase from the GT3 family based on the LG4X substitution model implemented in IQTREE. The chosen sequences were obtained from the NCBI database, aligned, and trimmed using MAFFT (v7.450) and TrimAl (V1.3) implemented in the website Phylemon 2 (http://phylemon2.bioinfo.cipf.es/). Similar taxa were collapsed for better readability. The numbers in parentheses represent the number of taxa in the clade that has been collapsed. The colors represent glycogen synthases GT3 from Euryarchaeota (blue), DPANN (yellow), Bacteroidetes (grey), PVC (red), and Eukaryota (green). The scale of the tree indicates a single substitution per site. Black circles symbolize ultrafast bootstrap support values greater than or equal to 80. The scale bar indicates the number of substitutions per site.

The putative GlgA-GT3 activities of archaea have not yet been enzymatically characterized. However, the presence of glycogen in methanogenic *M. thermophila* TM1 indicates that glycogen synthase GT3 is almost certainly functioning.^267^ To better understand the evolution of GlgA-GT3, a phylogenetic tree was inferred with glycogen synthase-GT3 sequences retrieved from PVC (Planctomycetes, Verrumicrobia, Chlamydiae), Eukaryota, DPANN, Euryarchaeota, and FCB group. As depicted in Figure 6, all Euryarchaeota GlgA-GT3 sequences are monophyletic and phylogenetically distinct from DPANN and other GlgA-GT3 sequences. This highlights a unique evolution of GlgA-GT3 in this group, which may be associated with the connection between glycogen and methanogenesis pathways in *Methanotrix* sp., *Methanococcoides* sp., and *Methanosarcina* sp. (Methanosarcinales order).^268^ In contrast, putative GlgA-GT3 sequences of the DPANN group are more closely related to PVC and heterotrophic eukaryotic organisms. GlgA-GT3 sequences belonging to Bacteroidetes members (FCB group) are nested inside the DPANN (Figure 6). A plausible hypothesis is that Bacteroidetes gained the GlgA-GT3 gene from the DPANN through horizontal gene transfer. It is worth noting that Bacteroidetes have conserved a fragment of the GlgA-GT5 gene in their genome.

As stated previously, heterotrophic eukaryotic glycogen synthases-GT3 require glycogenin activity to initiate glucan synthesis. Thus, a question naturally arises on the presence of glycogenin-like proteins in archaea. Interestingly a glycogenin-like protein was annotated (LC1Nh 1199) in *Candidatus Nanohalobium constans*. However, a homology search using the Hidden Markov model did not reveal any hit with eukaryotic glycogenin. To date, the existence of glycogen-like in DPANN remains questionable, and the enzymatic properties of archaeal GlgA-GT3 remain unknown.

It should be stressed that *P. torridus*, *C. divulgatum*, *A. cupricumulans*, and *Methanoculleus bourgensis* (Euryarchaeota) lack glycogen synthase activity and instead utilize the GlgE glycogen pathway, which is based on a putative maltose 1-phosphate:maltosyltransferase (Table 3).

As mentioned in section 3.2, maltose 1-phosphate serves as a building block for the GlgE pathway. The latter can be derived either from trehalose followed by two enzymes: a trehalose synthase (TreS) directly isomerizes maltose into trehalose (TreS-GH13 family), and then a maltokinase phosphorylates maltose into maltose 1-phosphate, or directly by the transglycosylation between ADP-glucose and glucose 1-phosphate via GlgM activity.

Except for vertebrates, trehalose is a ubiquitous disaccharide present in all living organisms. In response to environmental stress, it is produced via many routes. In Archaea, numerous trehalose pathways have been identified, including the TreT pathway, discovered in archaea and thermophilic bacteria, which synthesizes trehalose from NDP-glucose and glucose,^269−271^ the TreS pathway, mentioned above, found in *P. torridus*,^272^ and the TreX-TreY-TreZ path that converts glycogen into trehalose using three successive enzymes: TreX (glycogen debranching enzyme), TreY (maltooligosyl trehalose synthase), and TreZ (maltooligosyl trehalose hydrolase).^273^

**Figure 7:**
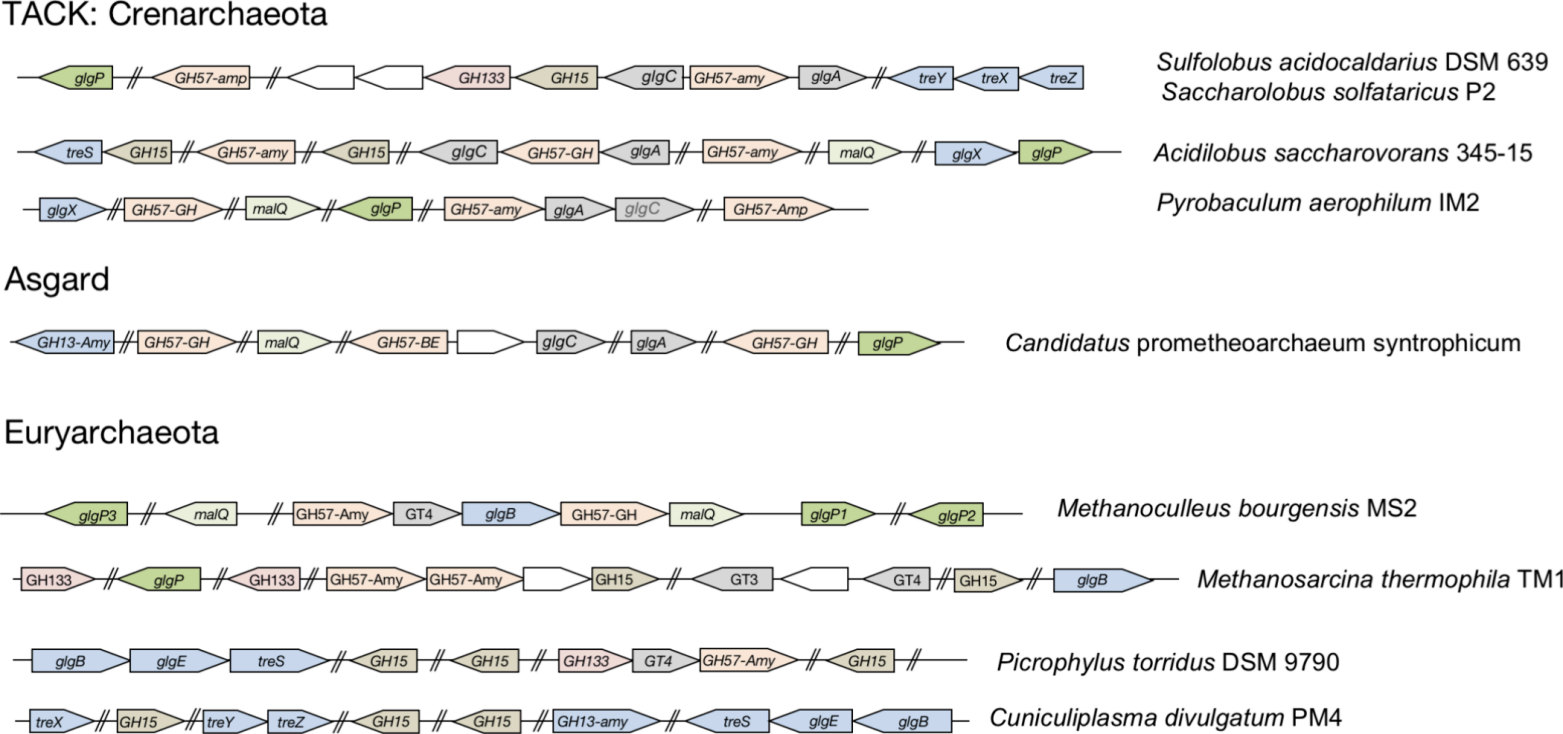
Arrangement of glycogen metabolizing genes in Archaea. The glycogen related genes were annotated according to the CAZy classification. Directional arrows denote transcriptional direction. Physical segregation between the genes is shown by parallel black lines. The white arrows represent unrelated glycogen genes. For clarity, the colored backgrounds of glycogen-related genes correspond to the enzymatic activities listed in Table 3. The number associated with GT or GH represents the family. GT3, GT3-glycogen synthase; GT4, NDP-glycosyl transferase; *glgA*, GT5-glycogen synthase; *glgB*, GH13-branching enzyme; *glgX/treX*, debranching enzyme; *treS*, trehalose synthase; *treY*, maltooligosyl trehalose synthase; *treZ*, maltooligosyl trehalose hydrolase; *glgP*, glycogen phosphosphorylase; GH57-BE, glycogen branching enzyme; GH57-GH, glycosyl hydrolase; GH57-amy, α-amylase-GH57; GH57-amp, amylopullulanase-GH57; GH15, glucoamylase; *glgC*, AGPase; GH133, amylo-α(1→6) glucosidase; *glgE*, maltose 1-phosphate transferase; *malQ*, α(1→4) glucanotransferase.

In the archaeal strains that harbor putative GlgE proteins, both putative TreS and Mak proteins were found in *P. torridus* (TreS, WP 011176870.1; Mak, WP 011176871.1), in *A. cupricumulans* (TreS WP 201779456.1; Mak, KQB33759.1), in *C. divulgatum* (TreS, SIM87424.1 A0A1N5WSF9), suggesting that GlgE path may operate. In *P. torridus*, a gene encoding a GT4 polypeptide (WP 011178040.1) forms a cluster with genes encoding putative amylo-α(1→6) glucosidase type debranching enzyme-GH133 and GH57-amylase (Figure 7). The GT4 family consists of several glycosyl transferase activities, including the recently identified GlgM enzyme of mycobacteria. However, the diversity of GT4 activities and the low level of similarity with mycobacterium GlgM (about 20%) make it difficult to conclude if GT4 gene encodes a GlgM-like activity.

Another intriguing situation is observed for the methanogenic archaea *Methanoculleus bourgensis* DSM3045 (Table 3; Figure 7). A survey of the genomic organization of the glycogen genes highlights an annotated gene (ID: IJ7JAJ9) that encodes a putative glycosyltransferase family 4 (GT4) nested among glgB and putative-amylase (GH57) genes. As revealed by a Blast search, the GT4 gene (BN140 RS11275) is confined to a few Euryarchaeal species. Given the absence of glycogen synthase GT3 or GT5 in the *M. methanoculleus* genome, it is tempting to speculate that the GT4 gene encodes an uncharacterized glycogen synthase.

#### 3.4.2 The Formation of α(1→6) Bonds in Archaea

The branching enzymes first cleave an α(1→4) glycosidic bond inside a glucan chain and subsequently transfer the glucosyl chain to the adjacent chain at the so-called branching point, or α(1→6) position. According to CAZy classification, branching enzymes fall into two glycosyl hydrolase families: GH13 and GH57. The GH57 was established due to an increasing number of enzymes lacking conserved regions characteristic of the GH13 family.^274^ Members of the GH57 family, which adopt the (β/α)_7_-barrel fold and five conserved domains,^275^ have been identified in archaea and a few eubacterial taxa, including cyanobacteria.^276^ Currently, the GH57 family encompasses various activity such as α(1→4)-glucanotransferase, amylopullulanase, β-galactosidase, maltogenic amylase, amylopullulanase-cyclodextrinase, α-amylase, and glycogen branching enzymes.^277^ 3D structures have been elucidated for several branching enzymes: *Thermococcus kodakarensis*,^278^*Thermotoga maritima*,^279^*Thermus thermophilus*,^280^ and *Pyrococcus horikoshii*.^281^

**Figure 8:**
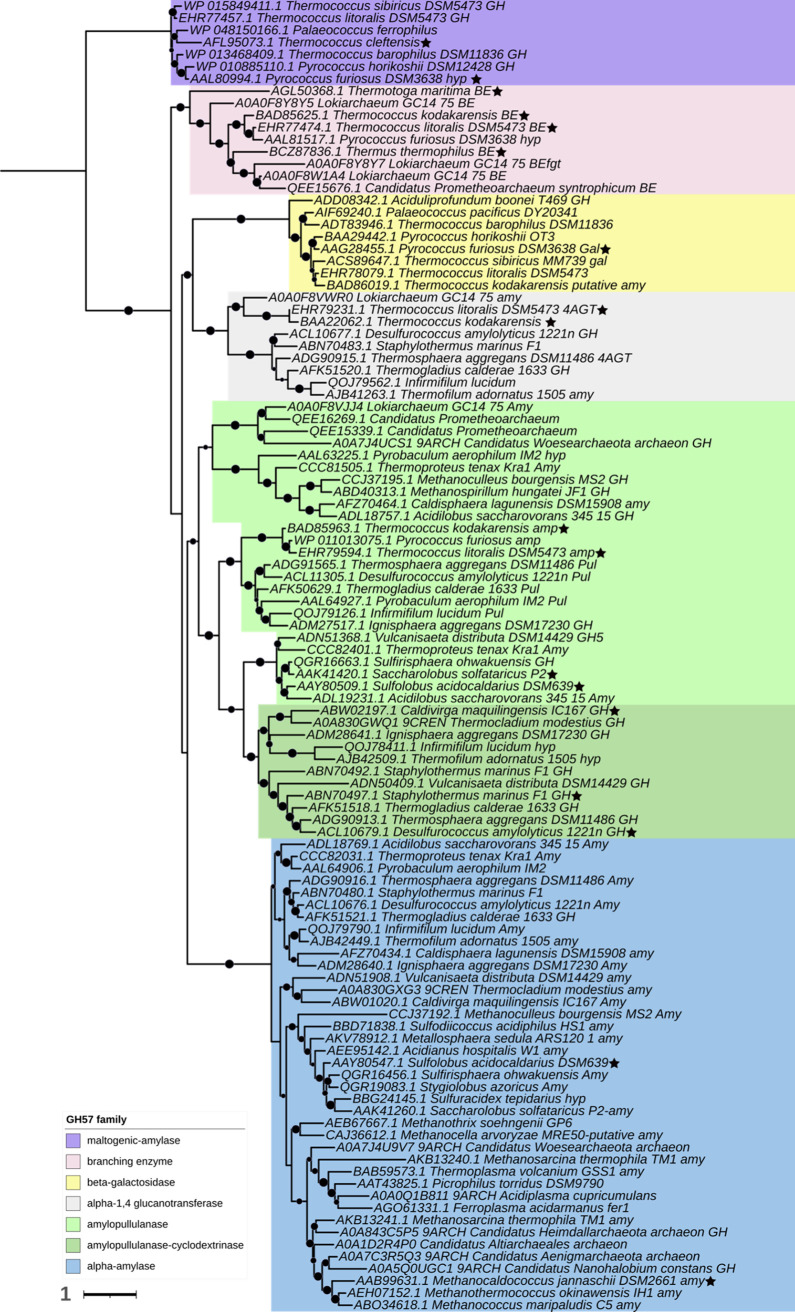
Maximum likelihood phylogeny of the archaeal GH57 family using the LG4X substitution model implemented in IQTREE. The chosen sequences were obtained from the NCBI database, aligned, and trimmed using MAFFT (v7.450) and TrimAl (V1.3) implemented in the website Phylemon 2 (http://phylemon2.bioinfo.cipf.es/). GH57 enzymes subjected to a detailed biochemical characterization are pointed by black stars. Black circles symbolize ultrafast bootstrap support values greater than or equal to 70. The scale of the tree indicates the number of substitution per site.

As indicated in Table 3, the glycogen branching enzymes GH13 and GH57 are restricted to Asgard and Euryarchaeota (for review, see ref (282)). Unlike the branching enzyme GH13, the archaeal branching enzymes GH57 of *P. horikoshii* and *Thermus thermophilus* possess both amylase and branching enzyme activities.^280,281^ The balance between amylase and branching enzyme activities in *P. horikoshii* is associated with the occurrence of a flexible loop that modifies access to the catalytic site.^281^ Astonishingly, no branching enzyme GH57 or GH13 activity was observed in the TACK group despite the presence of glycogen,^261^ strongly suggesting the existence of undiscovered branching enzyme activity. A phylogenetic tree of archaeal GH57-proteins was inferred based on the archaea listed in Table 3 and supplemented with additional sequences (Figure 8). The enzymatic properties were established according to the annotation of the CAZy database and bioinformatics approach.^283^Table 3 provides an overview of Archaea’s GH57-enzyme content. A striking observation is that α-amylase/α-amylase-like GH57 is preserved in all archaea lacking a “classical” branching enzyme (Table 3; Figure 8). Could this amylase-GH57 be the missing branching enzyme? It is important to note that only a few enzymatic characterizations have been conducted on these GH57-amylases. Kim and coworkers reported the characterization of the GH57-amylase (AAB99631.1) of *Methanocaldococcus janaschii*.^284^ However, the enzymatic characterization mainly focused on the hydrolysis activity of the recombinant GH57-amylase toward various polysaccharides. Considering that archaeal branching enzymes exhibit dual functions of amylase and branching enzyme activities, further investigations are warrented to determine whether the GH57-amylase of *M. janaschii* harbors a branching enzyme activity.

The characterization of a series of null mutants defective in the glycogen metabolism pathway of *Sulfolobus acidocaldarius* DSM 639 has provided some insight.^285^ Functional classification of enzymes can be achieved through glycogen content assays. Consequently, a mutation in either an anabolic or catabolic enzyme leads to a decrease or an excess of glycogen, respectively. All glycogen phenotypes were in line with the presumed function of the catabolic glucoamylase-GH15 and anabolic glycogen synthase mutants, except for the null GH57-amylase mutant, which displayed a dramatic decrease in glycogen level.^285^ This atypical phenotype could be explained by the lack of branches within the storage polysaccharide, rendering it extremely sensitive to intracellular hydrolase activities. Interestingly, a similar phenotype was documented in the Arabidopsis branching enzyme mutant, wherein the chloroplast harbors multiple β-amylase activities that prevent the accumulation of linear glucan chains.^286^

#### 3.4.3 The Catabolism Pathway in Archaea

The glycogen catabolism pathway involves a series of enzymes that hydrolyze α(1→4) and α(1→6) bonds. To complete glycogen digestion in *E. coli*, glycogen phosphorylase (GlgP) collaborates with the debranching enzyme (GlgX). Glycogen phosphorylase (GT35) converts the reducing end of glucan and orthophosphate into glucose 1-phosphate. The α(1→6) linkages or branching points impede further phosphorylase action, leading to the formation of phosphorylase limit-dextrin, which contains short-branched glucans (3 to 4 residues glucose). The debranching enzyme specifically trims these short glucan chains (GlgX), thus preventing a futile cycle of synthesis and degradation, as the GlgX is synthesized during glycogen synthesis. Maltodextrin phosphorylase (MalP) and α(1→4) glucanotransferase (MalQ) further contribute to the catabolism of the short glucans released by GlgX. This enzyme plays a central role in the short glucan metabolism during glycogen breakdown and in the maltose catabolism pathway by converting short glucans into longer glucans which are more accessible to maltodextrin phosphorylase for hydrolysis. It should be stressed that the null *malQ* mutant of *E. coli* cannot grow in the presence of maltose. In *Enterobacteriaceae*, the catabolic enzymes result in the release of glucose 1-phosphate and glucose from glycogen. Given this information, it is interesting to explore the catabolic process in Archaea.

#### 3.4.4 An Alternate Mechanism for Glycogen Breakdown in Archaea

Table 3 illustrates the variations in the number of glycogen phosphorylase genes in various archaeal strains. For instance, *Acidianus hospitalis* and *Stygiolobus azoricus* (TACK) lack glycogen phosphorylase activity, while *Methanotrix soehngensis* and *Methanoculleus bourgensis* (Euryarchaeota) possess two and three glycogen phosphorylases, respectively. These isoforms likely mirror their affinities for linear glucans and branched polysaccharides, as described for the maltodextrin and glycogen phosphorylase activities of *E. coli*. More significantly, the absence of glycogen phosphorylase activity in certain archaeal strains reveals a unique catabolic pathway where, at variance with enterobacteria, glycogen phosphorylase does not drive the glycogen degradation process but appears mostly devoted to the metabolism of imported short glucan.^287^ In line with this finding, a biochemical characterization of *glgP* null mutant of *S. acidocaldarius* displays a normal amount of glycogen in comparison to the wild-type strain, reinforcing the idea that GlgP is not a crucial enzyme in glycogen breakdown.^285^

The designations GlgX and TreX were assigned to the debranching enzymes based on their presence in the glycogen or trehalose operons. Both enzymes are considered to be identical in terms of enzymatic properties. However, the biochemical analysis of *Sulfolobus sp.* TreX reveals a bifunctional enzyme with α(1→4) glucanotransferase and amylo-α(1→6) glucosidase activity.^288,289^ Like in mammals, the α(1→4) glucanotransferase firstly cleaves the α(1→4) linkage prior to the last glucose residue hooked in α(1→6) linkage and transfers the glucan chain to the non-reducing end of an adjacent chain. Thanks to amylo-α(1→6) glucosidase, the residual branched glucose residue is eliminated. As described, the debranching step does not produce any short chains. A notable observation is the wide distribution of genes that encode amylo-α(1→6) glucosidase (EC3.2.1.33; GH133) activity among Archaea. The characterization of the null amylo-α(1→6) glucosidase mutant (GH133: AAY80544.1) of Sulfolobus, designated GlgX by the authors, results in an excess glycogen phenotype, suggesting its participation in the glycogen catabolism pathway.^285^

In contrast to *E. coli*, archaea possess additional hydrolytic enzymes, including glucoamylase-GH15 and different glycosyl hydrolases GH57, which are controlled by catabolic inhibition.^290^ These enzymes are secreted and have a function in starch utilization.^291,292^ The amylopullulanase mutant of *Sulfolobus acidocaldarius* DSM639, for instance, lost the capacity to thrive on a minimum medium containing starch as the only carbon source.^293^ Lee and colleagues have demonstrated that the starch medium stimulates the production of many extracellular catabolic enzymes in *P. furiosus*, including maltogenic amylase-GH57 and amylopullulanase-GH57.^287^ Two specialized transporters for trehalose/maltose and maltodextrin are then used to import maltose and maltodextrin inside *P. furiosus*.^294^ In the case of *P. furiosus* and other archaea lacking α(1→4) glucanotransferase activity GH77 or GH57, maltodextrins are likely further processed by glucoamylase-GH15/maltodextrin phosphorylase. Interestingly, in contrast to glycogen phosphorylase of *Thermococcus litoralis*, which liberates glucose 1-phosphate from maltodextrin containing at least four glucose residues,^295^ glycogen phosphorylases of *P. furiosus* can digest a glucan chain up to maltose, allowing complete hydrolysis into glucose by a glucoamylase-GH15.^296^

## 4 Chemical Reactions in α-Glucan Synthesis and Degradation

**Figure 9:**
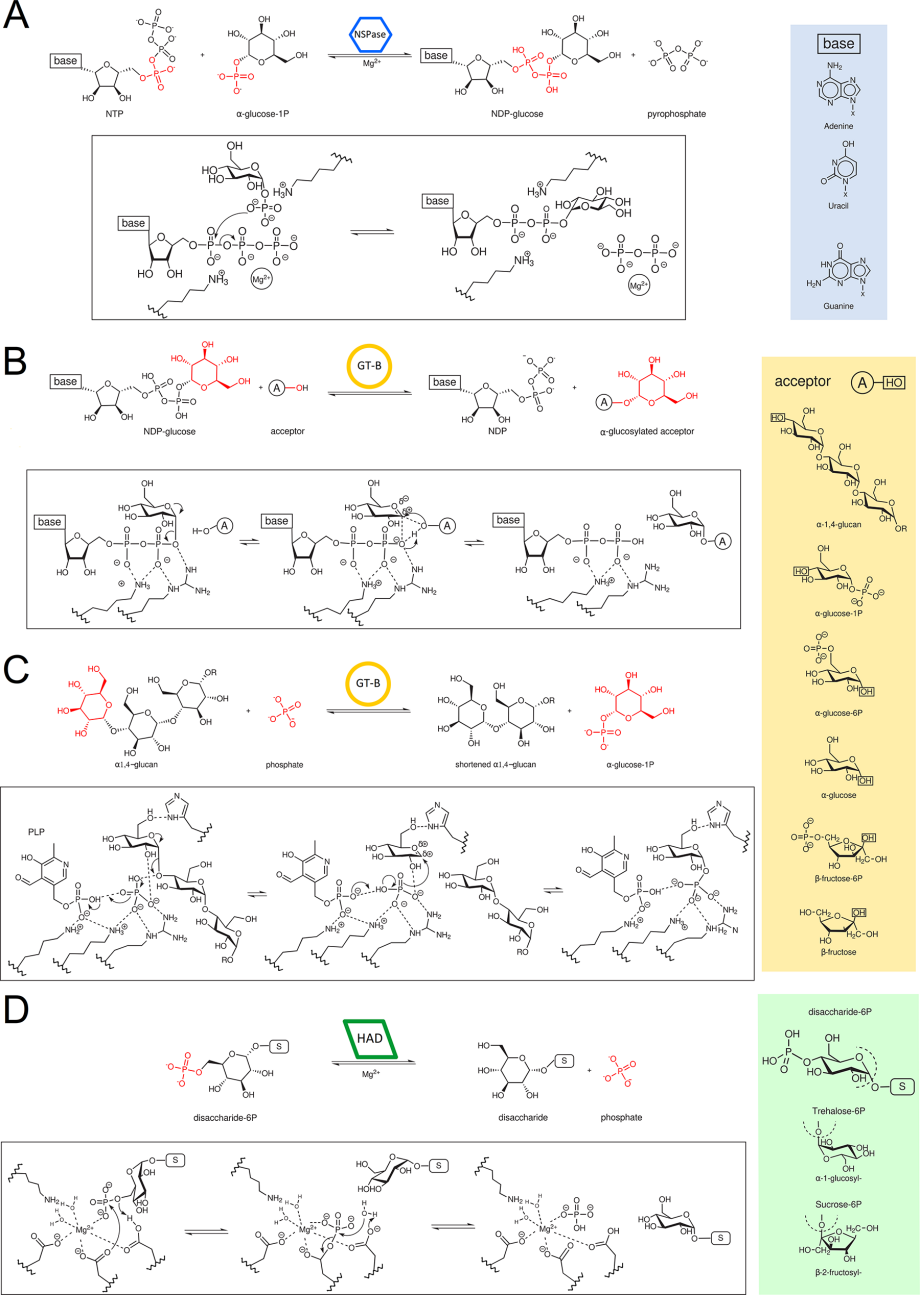

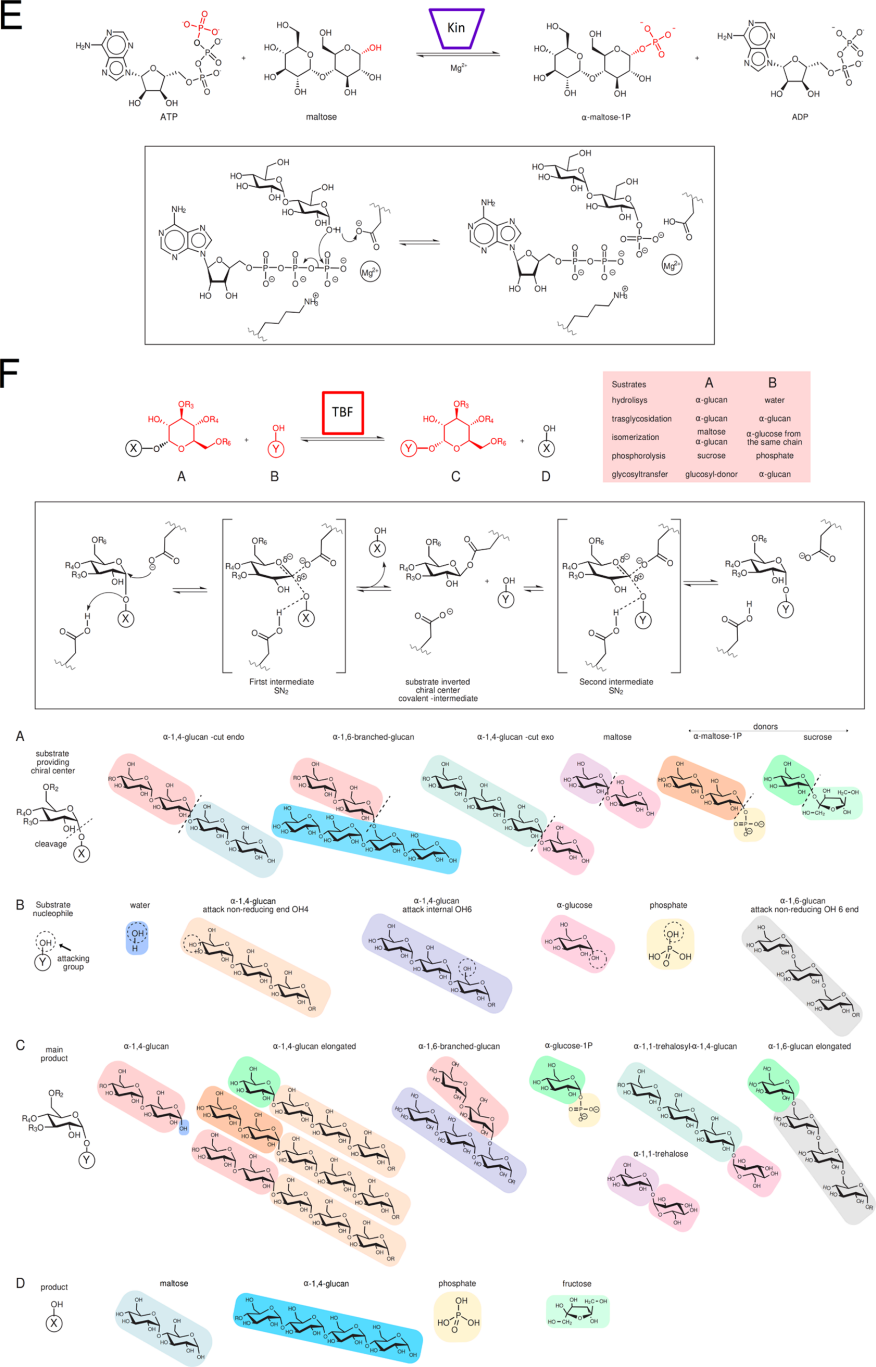
Reactions and catalytic mechanism of enzymes in the α-glucan and disaccharide metabolism. Intervening chemical groups and molecule motifs from the substrates and products representations are colored in red. (A) *Top view*, the general reaction for nucleotide glucose biosynthesis mediated by nucleotide glucose pyrophosphorylases. The generic nucleotide triphosphate reacts with glucose 1-phosphate (glucose 1P) in the presence of Mg^2+^ to bring forth the corresponding nucleotide diphospho-glucose and pyrophosphate. Frequently used nucleotide bases are indicated on the right on a blue-shaded box. *Bottom view*, the catalytic mechanism of phosphotransfer mediated by the nucleotide glucose pyrophosphorylase, involves a couple of lysine residues that polarize the phosphoester bonds. (B) *Top view*, the general reaction for glucose transfer mediated by glycosyltransferases. The generic nucleotide diphosphate-glucose and an acceptor bearing a nucleophilic hydroxyl group react to produce a nucleotide diphosphate and the glucosylated product. Examples of acceptor substrates are displayed on the right on a yellow shaded box with the reactive hydroxyl group marked with a rectangular box. *Bottom view*, the proposed S_N_i-type catalytic mechanism for most of the retaining glycosyltransferases. (C) *Top view*, the general reaction for the phosphorolysis of a non-reducing end of an α-glucan to form glucose 1-phosphate. *Bottom view*, the 5'-phosphate group of the essential cofactor pyridoxal phosphate functions as an acid-base to promote attack by the substrate phosphate on the the α-glucan anomeric carbon. (D) *Top view*, the general reaction of disaccharide 6-phosphate phosphatases. Frequently used substrates are indicated on the right on a green shaded box. *Bottom view*, the catalytic mechanism showing a group of carboxilic groups from acid residues (Asp/Glu) that coordinates a Mg^2+^ atom, with one also acting in the nucleophilic attack of the phosphate group to formg a phospho-protein covalent intermediate. In a second step, an activated water molecule attacks the covalent intermediate, releasing phosphate. (E) *Top view*, the general reaction of maltokinase, showing the reaction between the ATP with maltose to bring forth maltose 1-phosphate. *Bottom view*, the proposed catalytic mechanism showing the nucleophilic attack of the anomeric OH group of maltose to the γ-phosphate group of ATP. (F) *Top view*, A general reaction for the catalysis mediated by TIM-barrel folds in the context of α-glucan metabolism. Depending on the nature of the reactions presented in the red shaded box, substrates (A and B) outcomes products (C and D). *Central view*, a general two-step double inversion S_N_2 mechanism, showing the participating acidic residues acting as a base and forming the covalent intermediate. Possible hydrogens or α-linked-chains substitutions in the α-glucose moiety are indicated as R groups in the pyranose ring (R2, R4, and R6) to represent the variable nature of the substrates under this mechanism. *Bottom view*, a brief list of substrates (A and B) and products (C and D), highlighting in different shade colors the other moieties exchanged in the reaction, permitting to read from top to bottom different reactions. For example, α(1→4)-glucan (substrate A) reacts with water (substrate B) bringing a remaining α(1→4)-glucan (product C) and maltose (product D), whereas maltose 1-phosphate (substrate A) and α(1→4)-glucan (substrate B) brings forth an elongated α(1→4)-glucan chain (product C) and phosphate (product D).

The evolution of enzymatic reactions and catalytic/molecular/structural mechanisms play key roles in the articulation of (new) metabolic pathways. α-Glucan metabolism offers a remarkable framework of catalysts using a defined building block, α-d-glucose, and a few ancillary substrates for its activation and polymerization. Altogether, each of these enzymes can be considered a piece of machinery dedicated to preserving the stereochemistry of α-d-glucose. Although certain underlying mechanisms are common among enzymes involved in bacterial α-glucan metabolism, as will be evident later, the overview presented here points to fundamental aspects of the building block processing. Therefore, the enzymatic reactions comprise three groups: (i) the synthesis of gluco-saccharide donors, (ii) the formation of α-glucosyl bonds between donors and sugar acceptors, and (iii) the disruption of glucosyl bonds. Here we present these chemical reactions and base mechanisms that present the enzymes-domain that perform such functions (Figure 9).

### 4.1 The Synthesis of Glucose Nucleotide Donors

The synthesis of NDP-glucose donors follows a mechanism parallel to other nucleotide-activated compounds.^297,298^ Two enzymes are dedicated to the synthesis of ADP-glucose and UDP-glucose, the two major glucose donors in bacterial α-glucan metabolism, AGPase (EC 2.7.7.27)^184^ and UGPase (EC 2.7.7.9),^299−301^ respectively. GGPase, which synthesizes GDP-glucose (EC 2.7.7.34), can also play a role in the synthesis of trehalose in a few bacteria.^302,303^

NDP-glucose donors are synthesized as a condensation reaction between NTP and glucose 1-phosphate following a bimolecular nucleophilic substitution S_N_2(P) that exchanges the phosphate-ester bond from the NTP to the glucose 1-phosphate.^304^ The transfer of the nucleoside monophosphate occurs via the nucleophilic attack of an oxygen atom from the PO_4_ group in glucose 1-phosphate to the phosphorus atom in the α-PO_4_ from NTP, producing the displacement of PPi via a pentavalent phosphate transition state, and leading to the inversion of the NTP α-PO_4_ upon nucleophilic attack. Notably, the reaction is held in the presence of a divalent metal cation with a strong preference for Mg^2+^.^305,306^ The divalent metal cation minimizes the repulsion between different PO_4_ groups and lowers the energetic barrier of the enzymatic triphosphate hydrolysis, weakening the leaving-group bond, stabilizing the transition state, and strengthening the interaction with the nucleophile.^307^ In addition, positively charged residues in the active site coordinate the substrate's PO_4_ groups in place.^308^ Finally, proton exchange is balanced by the solvent. Overall, the energy balance in breaking and forming the phosphodiester group is near zero; therefore, the reaction is reversible. Nevertheless, hydrolysis of pyrophosphate catalyzed by inorganic pyrophosphatases pulls this reaction to completion^309^ (Figure 9A).

### 4.2 Glucosyl Transfer Reactions

#### 4.2.1 Glucosyl Transfer from an NDP-Glucose Donor

NDP-sugar-dependent glycosyltransferases (GTs) act on a broad range of acceptors to create new glycosidic linkages, generating a significant amount of structural diversity in biological systems.^310−312^ The transfer of a glycosyl group can proceed with retention or inversion of the anomeric configuration of the product with respect the donor substrate, and consequently, GTs are classified as ‘retaining’ or ‘inverting’.^310,313−315^ In reactions involving α-glucan biosynthesis from an NDP-α-d-glucose donor, a ‘retention’ mechanism is obliged. The reaction mechanism for ‘retaining’ GTs has been a matter of debate. By analogy with glycoside hydrolases, a double-displacement mechanism was first proposed.^310,313−315^ This mechanism requires a suitable positioned nucleophile, typically Glu or Asp, in the catalytic center that mediates the formation of a glycosyl-enzyme intermediate during the first part of the reaction, resulting in a first anomeric inversion. In the second part of the reaction, the activated acceptor substrate attacks the glycosyl-enzyme intermediate, resulting in the formation of a product with total retention of the anomeric configuration.^314^ Amino acid sequence alignments do not show consistently conserved amino acid residues for such an important role. Members of family 6 GT were first predicted to display such a putative nucleophile.^315,316^ Quantum mechanics/molecular mechanics (QM/MM) metadynamics analysis of the bovine α(1→3)-galactosyltransferase (α3GalT) GT6 mechanism supports that the donor saccharide forms a covalent Glu-Gal intermediate adduct prior to transfer to the acceptor.^316,317^ However, experiments with human GTA/GTB GT6s found that mutating Glu303 to Cys or Asp only slightly slows down the reaction.^318,319^ Native ternary complexes of the bovine α3GalT GT6 in the presence of UDP-Gal, lactose, and the divalent cation cofactor, revealed the substrates are organized very similarly to other ‘retaining’ GTs, with the putative nucleophile Glu317 participating in a hydrogen bond with the acceptor β-Gal O4 atom, supporting a role in acceptor binding.^320,321^ As an alternative possibility, an unusual single-displacement mechanism named as ‘front-face’ or S_N_i, substitution nucleophilic internal-like mechanism, was proposed, in which the nucleophilic attacks from the same face as the leaving group departure. Specifically, the acceptor hydroxyl nucleophile is deprotonated by the donor β-phosphate oxygen and attacks the anomeric carbon atom of the sugar donor from the same side as the leaving nucleotide, and involves a short-lived oxocarbenium ion intermediate.^310,316^ Several structural snapshots of enzyme-donor-acceptor Michaelis complexes for ‘retaining’ GTs strongly support the S_N_i-type mechanism, including the glucosyl-3-phosphoglycerate synthase (GpgS) from *M. tuberculosis* and the xyloside α-(1→3)-xylosyltransferase XXYLT from *Mus musculus*(310,322) (Figure 9B).

Very recently, mass spectrometry analyses of WbbB from family GT99, an enzyme that adds a terminal β-Kdo (3-deoxy-d-*manno*-oct-2-ulosonic acid) residue to the *O*-antigen saccharide, forms a covalent adduct between the catalytic nucleophile, Asp232, and Kdo.^323^ Similarly, the formation of covalent adducts with Kdo were reported in a member of the GT107 family, KpsC, a dual-module enzyme that is essential for biosynthesis of ‘group 2’ capsular polysaccharides in *E. coli* and other Gram-negative pathogens.^324^ Crystal structures of WbbB and KpsC variants revealed the presence of Kdo-adducts, supporting the occurrence of a double displacement mechanism in this two GT families, rather than the generally observed S_N_i mechanism.^323,324^ This suggests that despite the considerable divergence between WbbB (GT99) and KpsC (GT107), the presentation mode of Kdo in the active site is likely a key feature of the mechanism in both enzyme families. The evolutionary origins of these enzymes remain an intriguing area of research that encourages further study and exploration.^323,324^

The SNi mechanism is proposed to occur in several enzymes in the α-glucan metabolism, where the nucleophilic attack comes from different acceptors leading to other glucoside products. Examples of these enzymatic activities are (i) glycogen synthase GS (EC: 2.4.1.11 and EC: 2.4.1.21),^325−327^ where the attack of the OH_4_ group from the non-reducing end of α(1→4)-glucan elongates the glucan chain; (ii) the maltose 1-phosphate synthase GlgM (EC: 2.4.1.342;^119^ where the attack comes from the OH_4_ group from DG1P; (iii) the trehalose 6-phosphate synthase OtsA (EC: 2.4.1.15)^328^ and the trehalose synthase TreT (EC 2.4.1.245)^271^ where the attack comes from the OH_1_ group from either α-d-glucose or DG6P; and (iv) the sucrose synthase SUS (EC: 2.4.1.13),^329^ and sucrose-phosphate synthase SPS (EC 2.4.1.14),^330^ where the OH_2_ group attack comes from either d-fructose or F6P.

#### 4.2.2 Maltose Transfer

A particular reaction for generating a new bond between two α-d-glucose moieties is catalyzed by the α(1→4)-glucan:maltose-1-phosphate maltosyltransferase GlgE (EC 2.4.99.16),^211,331^ a GH13 family enzyme^246^ which transfers maltose from maltose 1-phosphate to the non-reducing end of an α-glucan chain. This enzymatic reaction follows a SN_2_-type double displacement mechanism that retains the configuration of the α-glucose, and has experimentally been well supported at structural level.^216,218,219,221^ Specifically, in the first step, an aspartic acid residue acts as a nucleophile/base attacking the C1 position of maltose 1-phosphate while a second acidic residue donates a proton to the phosphate group to facilitate the release of the leaving group. This step ends in a transient covalent intermediate with the aspartate bound to C1, with the inversion of the anomeric configuration of the participating glucose moiety in maltose. In a second step, the OH group from the C4 non-reducing end of the glucan acceptor attacks the C1 generating the new glycosidic linkage. This second attack causes the scission of the covalent-intermediate bonding regenerating the nucleophile/base, leading to the second inversion of the glucose anomeric configuration, releasing an α(1→4)-glucan elongated with two glucose moieties (Figure 9F).

#### 4.2.3 Glucose Transfer from Sucrose

Another mechanistic alternative for α-glucan elongation is presented by the so-called glucansucrases, a group of bacterial enzymes that use sucrose as a donor for α-glucan synthesis. Here the glycosyl transfer occurs through a concerted mechanism that involves sucrose hydrolysis generating a glucosyl-protein covalent intermediate, and the subsequent transfer of the glucose moiety to the acceptor.^85,126,332,333^ These enzymes belong to the GH13 and GH70 families, displaying a mechanism similar to hydrolase and transglycosylase reactions. A glutamic acid residue protonates the sucrose oxygen linkage assisting the sucrose glycosidic bond break, releasing a fructosyl group. An aspartic acid residue located on the opposite side of the sugar ring attacks the glucose C1 leading to the formation of a covalent glycosyl-enzyme intermediate (with transient change in the anomeric configuration of glucose). The intermediate is rapidly broken by the nucleophilic attack from an OH group that is activated by the glutamic acid residue, transferring the glucose while reversing the anomeric confirguration, an overall retaining mechanism.^334^ Typical activities include (i) amylosucrase (EC 2.4.1.4)^243,335^ that forms α(1→4)-glucan linkages; (ii) dextransucrases (EC 2.4.1.5;^336,337^ that produce α(1→6)-glucan linkages; and (iii) mixed mechanism enzymes as alternansucrases (E.C. 2.4.1.140)^338^ synthesizing α-glucans with alternating α(1→3) and α(1→6), α(1→4)-glycosidic bonds, and reuteransucrase) producing α(1→4) and α(1→6) linkages resulting in no heterogeneous structure with no repeating units^339^ (Figure 9F).

### 4.3 Glucosidic Bond Breakage

#### 4.3.1 Glucosidic Bond Hydrolysis

Glycosidic bond hydrolysis can take place at the extremes or within the α-glucan chain, depending on the enzyme architecture and specificity. Interestingly, many of these enzymes rely on a common reaction involving a double displacement mechanism with the formation and hydrolysis of a covalent glucosyl-enzyme intermediate. Indeed, all the glycoside hydrolases discussed here are comprised in the GH13 family, displaying a retaining mechanism.^340^ In a first step, the glucose anomeric carbon undergoes a nucleophilic attack by a catalytic aspartate residue with the concomitant donation of a proton to the oxygen glycosyl linkage from a glutamic acid residue. The attack leads to the formation of the glucosyl-enzyme covalent intermediate displaying the inversion of the glucose anomeric center. In a second step, the intermediate undergoes the nucleophilic attack of the anomeric center by a water molecule activated by the glutamic acid residue resulting in an oxocarbenium state that transitions into the inversion of the anomeric center, releasing α-d-glucose.^61,341^ Enzymes displaying this mechanism include (i) α-amylases MalS, AmyA, AmyQ (EC 3.2.1.1),^342−344^ which hydrolyzes α(1→4)-glucan linkages; (ii) α(1→6)-glucosidases (EC 3.2.1.10, EC 3.2.1.20, EC 3.2.1.70);^345,346^ (iii) trehalose 6-phosphate hydrolase TreA/TreC (EC 3.2.1.93);^347^ (iv) TreY that releases trehalose from α(1→4)-glucan-α-d-glucanosyl-trehalose (EC 3.2.1.141);^348^ and (v) glycogen debranching enzyme GlgX that hydrolyzes α(1→6)-glucan linkages (EC 3.2.1.68)^235^ (Figure 9F).

#### 4.3.2 Glucosidic-Phosphate Bond Hydrolysis

The removal of phosphoryl groups from trehalose 6-phosphate and sucrose 6-phosphate is the final step for synthesizing the corresponding disaccharides. These reactions are catalyzed by two enzymes belonging to the haloacid dehydrogenase (HAD) superfamily, (i) trehalose 6-phosphate phosphatase (EC: 3.1.3.12; OtsB or TPP)^349−351^ and (ii) sucrose 6-phosphate phosphatase (EC: 3.1.3.24, SPP).^352,353^ The scission of the phosphoryl group from the sugar relies on a divalent metal cation, Mg^2+^, for activity, which is coordinated to a pair of carboxylates group from aspartic residues. The catalytic mechanism was extensively studied using the phosphoserine phosphatase homologue as a model (PSP).^354,355^ PSP hydrolyses the phosphoryl group of phospho-l-serine to produce serine with the generation of a phosphor-enzyme intermediate, which is further hydrolized leading to the product release. An oxyanion hole comprising two aspartic acids and a lysine residue and the divalent metal cation Mg^2+^, stabilizes the negative charge of the deprotonated oxygen, while a third aspartic acid residue performs a nucleophilic attack into the 5′-phosphate group leading to a putative pentavalent transition state. Finally, in PSP, an aspartate residue assists in protonating the serine product, activating/deprotonating water that attacks the intermediate releasing phosphate. The dephosphorylation of the disaccharide phosphate mediated by TPP and SPP presumably follows the same mechanism, not only due to the strict conservation of the catalytic residues in the active site but also supported by single-point mutagenesis analysis^349,352,356^ (Figure 9D).

#### 4.3.3 Phosphorolysis

A basic mechanism for breaking the glycosidic bonds of α(1→4)-glucan consists of phosphorolysis, exhibited by enzymes that present pyridoxal phosphate (PLP) as a covalently linked coenzyme in the active site, facilitating acid/base catalysis.^198,357−359^ This is the mechanism exhibited by bacterial glycogen phosphorylase (GP) and maltodextrin phosphorylase (MalP; EC 2.4.1.1), homologs of the classical eukaryote GPs.^196^ Specifically, an inorganic phosphate anion receives a proton from the PLP phosphate group. This exchange is stabilized by the coordination of PLP by protein basic groups. Concomitantly, the α(1→4)-glucan substrate interacts with the phosphate bringing the glycosidic bond close to the PLP, resulting in the transfer of a proton from the phosphate group to the oxygen linkage breaking the α(1→4)-glycosyl linkage generating a 4′-OH group nonreducing end of the leaving chain. As a result, a C1 carbocation intermediate is generated at the non-reducing end of the glucose moiety, which is attacked by the phosphate anion in a nucleophilic addition that produces the second product glucose 1-phosphate with retention of the α configuration. Importantly, this is a reversible reaction mechanism, that can perform an α(1→4)-glucan synthase activity acting as a glucose 1-phosphate-dependent glycosyltransferase *in vitro*(196,360) (Figure 9C).

In addition to the described mechanism, a group of enzymes of the GH13 family in bacteria display disaccharide phosphorylase activities.^246^ Specifically, some bacteria bear sucrose phosphorylases (EC 2.4.1.7),^361,362^ and the sucrose 6(F)-phosphate phosphorylase S6FP (EC 2.4.1.329).^363^ These enzymes also break down the glycosyl bond in a PLP-independent manner. Their phosphorolysis mechanism is similar to previously described hydrolytic enzymes, involving a double displacement mechanism but with Pi as an attacking group (Figure 9F).

### 4.4 Cut and Paste Enzymes

#### 4.4.1 Trans-glycosylation

A group of enzymes of the GH13 and GH77 families have the ability to reprocess segments of the α-glucan chain.^246,364^ These enzymes display an α-glucan transferase activity based on the classic hydrolytic double inversion mechanism. After hydrolysis, the catalysis continues with the synthesis of a new glycosyl bond since the covalent intermediate is attacked by a OH group of an α-glucan acceptor, which is a better nucleophile than water. It is worth noting that hydrolysis can also compete. Therefore, these transglycosylation reactions present a kinetically controlled mechanism.^365^ Specifically, the first step of the reaction involves the nucleophilic attack of the anomeric carbon by an aspartic acid group, and the concomitant donation of a proton from a glutamic acid residue to the glycosidic oxygen leading to the formation of a glycosyl-enzyme intermediate. In the second step, the glutamate residue deprotonates an OH group of the α-glucan acceptor which attacks the anomeric carbon releasing the final product with retention of the anomeric configuration.^341,366^ Two enzymes display this mechanism: (i) the glycogen branching enzyme GlgB (EC 2.4.1.18)^364^ transfers a segment of an α-(1→4)-glucan chain to an internal OH6 group in the same or neighboring α-glucan chain, forming a new branch joined with an α(1→6)-glycosidic bond, and (ii) the amylomaltase 4-α-glucanotransferase MalQ (EC 2.4.1.25)^341,366,367^ that transfers an oligo α(1→4)-glucan to a new position in an acceptor, which may be glucose or a (1→4)-α-d-glucan (Figure 9F).

#### 4.4.2 Isomerization

A special mechanism in saccharide remodeling involves the glycosyl-bond isomerization between glucose moieties. Two enzymes belonging to GH13 family^246^ display this capacity in the context of α-glucan metabolism: (i) maltooligosyl trehalose synthase TreY (EC 5.4.99.15)^368^ converts the glucosidic bond between the two last glucose residues of an α(1→4)-glucan chain into an α(1→1) bond, generating a non-reducing end, and (ii) trehalose synthase TreS (EC 5.4.99.16)^232,369^ catalyzes the conversion of the α(1→4) bond in maltose to an α(1→1) bond forming trehalose. Therefore, these enzymes are isomerases with intramolecular transferase activity. In the case of TreS, a reaction with 5-fluoroglycosyl fluorides leads to a covalent glycosyl-enzyme intermediate consistent with a two-step, double displacement mechanism.^370^ In the case of TreY, a pocket at the active site cavity recognizes the substrate enabling the rotation of the separated +1 glucose to form the α(1→1) bond^368^ (Figure 9F).

### 4.5 Maltoside Phosphorylation

Besides GlgM NDP-sugar-dependent synthesis of maltose 1-phosphate, some bacteria produce this donor by the action of a maltokinase (Mak/pep2, EC 2.7.1.175).^229,371^ Interestingly, in some organisms Mak form a complex with TreS, an oftentimes fused forming a unique protein Mak/TreS.^213^ Mak uses ATP as the main phosphate donor and the divalent metal cation Mg^2+^ for maximal enzyme activity. The OH1 group of maltose is oriented towards a catalytic aspartate residue, which act as a base to abstract the proton, attacking the γ-phosphate of ATP. A second aspartate residue positions the divalent metal cation, coordinating the ATP phosphate similarly to the aminoglycoside phosphotransferase (EC 2.7.1.95)^372−376^ (Figure 9E).

## 5 The Catalytic Folds Observed in Prokaryotic α-Glucan Processing Enzymes

The tertiary structure of the catalytic domains of enzymes involved in the α-glucan and disaccharide metabolism is substantially dominated by the α/β topology, mainly comprising Rossmann-like and TIM barrel folds. This aligns with the assumption that this structural class of globular proteins appeared early in evolution.^377^ A view on how metabolic pathways evolve conceptualizes survival of the fittest applies not only to genes, and to their encoded enzymes, but also their metabolites. This hypothesis proposes that the origin of new metabolites and new enzymes in a network arise from enzyme-metabolite coevolution, driving enzyme recruitment and pathway evolution.^378^ In this context, we present selected structural information from bacterial α-glucan and disaccharide processing enzymes. Structures are presented clustered by catalytic fold, enzymatic reaction, and metabolites to reflect underlying structural evolutive commonalities on the enzymatic pieces of the machinery handling the α-glucose moiety.

### 5.1 The Rossmann Fold

**Figure 10:**
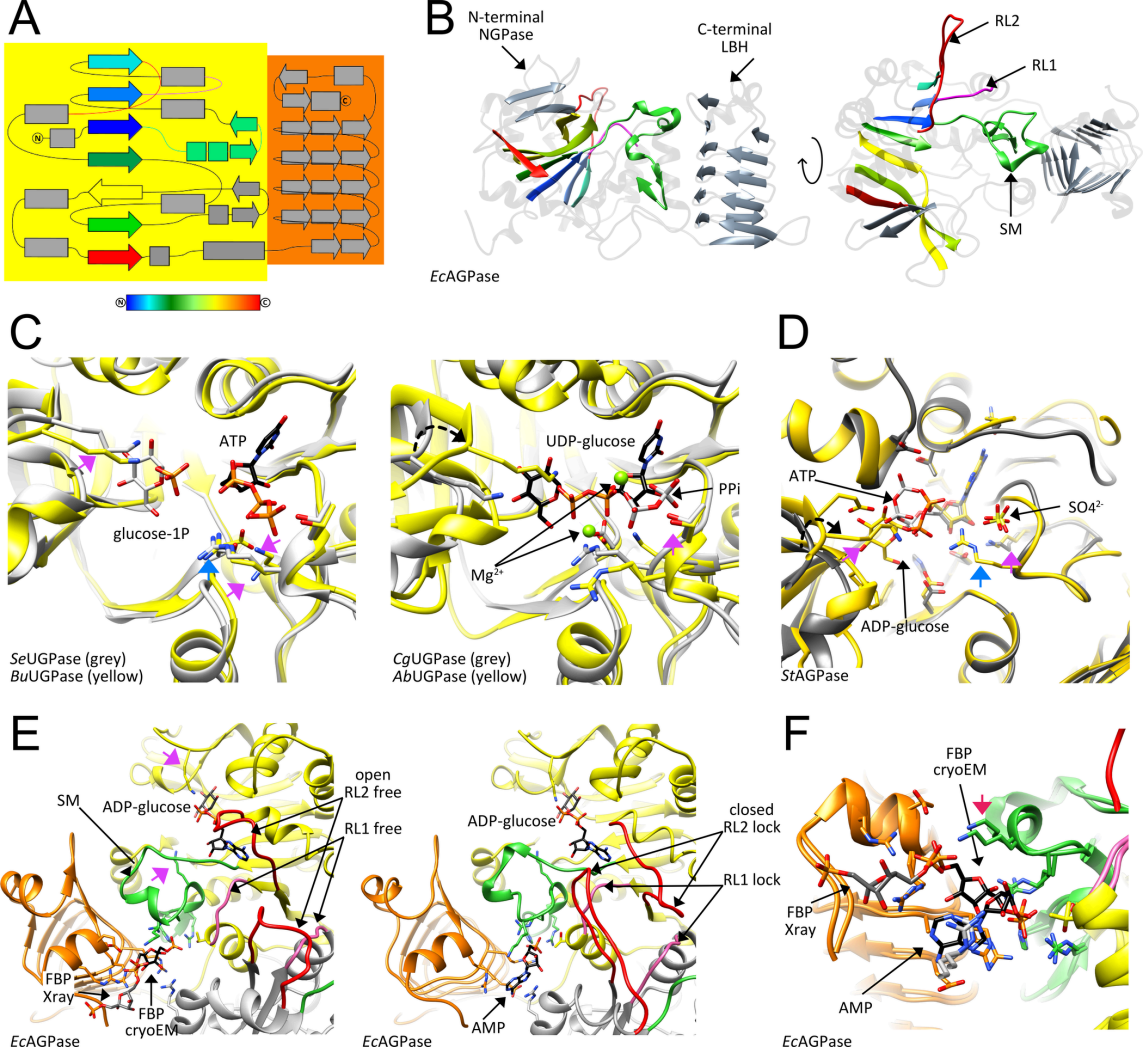
Structure, catalysis, and regulation of NDP-glucose pyrophosphorylases. (A) Schematic representation of the secondary structure of AGPase showing bars representing α-helices and arrows as β-strands. The highlighted domains show the Rossmann fold catalytic domain (yellow background) and the C-terminal left-handed β-helix domain (orange). Arrows of the central β-sheet of the Rossmann fold domain are colored according to the key bar indicating the direction N-terminal to C-terminal. (B) Two views of the overall architecture of AGPase from *E. coli* as visualized by CryoEM (PDB 6R8U; grey).^186^ (C) Close views of the active sites of selected UGPases showing the superposition of complexes with ligands. *Left panel*, the structural superposition of the UGPase from *Sphingomonas elodea* in complex with glucose 1-phosphate (PDB 2UX8, grey)^420^ with that of *Burkholderia ambifaria* in complex with UTP (PDB 5VE7, yellow).^668^*Right panel*, the structural superposition of the UGPase from *Corynebacteria glutamicum* in complex with UDP-glucose and Mg^2+^ (PDB 2PA4, grey)^422^ with that of *Acinetobacter baumanii* in complex with UTP and pyrophosphate (PDB 6IKZ, yellow).^669^ (D) Structural superposition of two AGPase complexes from *S. tuberosum* with ATP (PDB 1YP3, grey)^404^ and ADP-glucose (PDB 1YP4, yellow).^404^ Equivalent residues shown in (C) and (D) are highlighted by colored arrows. (E) The allosteric regulatory mechanism of AGPases. Two views of a selected protomer of the tetrameric AGPase from *E. coli* in complex with the preferred allosteric positive regulator FBP, as observed by X-ray crystallography and CryoEM; N-terminal domain, yellow; C-terminal domain, orange). The ADP-glucose ligand was placed based on the AGPase complex from *S. tuberosum* (PDB 1YP4).^404^ The secondary structure elements involved in the regulatory mechanism, the SM (green) communicates the regulatory cleft providing an intra-protomer signal. The RL1 (pink) and RL2 loops (red) are displayed in open conformation. (F) Structural superposition of the preferred positive and negative allosteric regulators, FBP and AMP, respectively, as observed in the corresponding X-ray crystallography and CryoEM structures.^186,188,408^

First described for the structure of lactate dehydrogenase (LDH),^379^ Rossmann folds (Figure 10) are widespread across the whole metabolism in all living organisms and are the most frequent protein fold in the Protein Data Bank.^380^ The Rossmann fold was identified by comparing the tertiary structure of LDH with other nucleotide-cofactor binding enzymes,^379^ revealing the arrangement between β-strands and α-helices with a common handedness for the conserved nucleotide-binding motif, suggesting a very distant divergent evolution or a convergence folding-to-function in these enzymes. Indeed, this fold was estimated present at a precellular life origin, according to measurements of evolutionary distance based on structural sequence aligned structures of nucleotide binding enzymes, including dehydrogenases, kinases, and flavodoxins.^381^ Currently, these views are still under debate as some structural analysis suggests these types of folds could have evolved independently multiple times into different Rossmann-like groups revealing convergent evolution.^380^ In contrast, other studies indicate the existence of an ancestral βαβ segment that comprises the core resulting to be the seed for the evolution of these enzymes.^382^

The classical Rossmann fold topology comprises a three-layered α/β/α sandwich formed by consecutive βαβαβ super-secondary structures, in which β-strands assemble forming a central β-sheet, with strand order classical topology 321456.^383^ The βαβ segment is proposed as the minimal Rossmann-like motif where β-strands and α-helices are packed by lateral interactions connected by loops of variable size. The connecting loops between certain secondary structure elements comprised in the α/β/α sandwich topology and the central β-sheet contribute to build up a cleft that accommodates the active site. Some of these loops are key elements for the binding of phosphorylated nucleoside substrates in other enzymes including dehydrogenases, NTPases and kinases.^384−386^ Known as Phosphate-binding-loops (P-loops) and Glycine-rich-loops (G-loops),^387^ these structural elements contain conserved sequences so-called Walker-motifs, comprising the motif A, GxxGxGK[S/T] and motif B, DxxG where x is any residue and h correspond to a hydrophobic residue.^384−386^ Interestingly, the possibility of transferring the biochemical function by insertion of these loops in small designed proteins suggests these minimal elements emerged for nucleotide binding and catalysis, evolving later upon acquisition of higher sequence and structural complexity.^388^

#### 5.1.1 Nucleotidyl-Transferases NDP-Sugar Pyrophosphorylases

**Figure 11:**
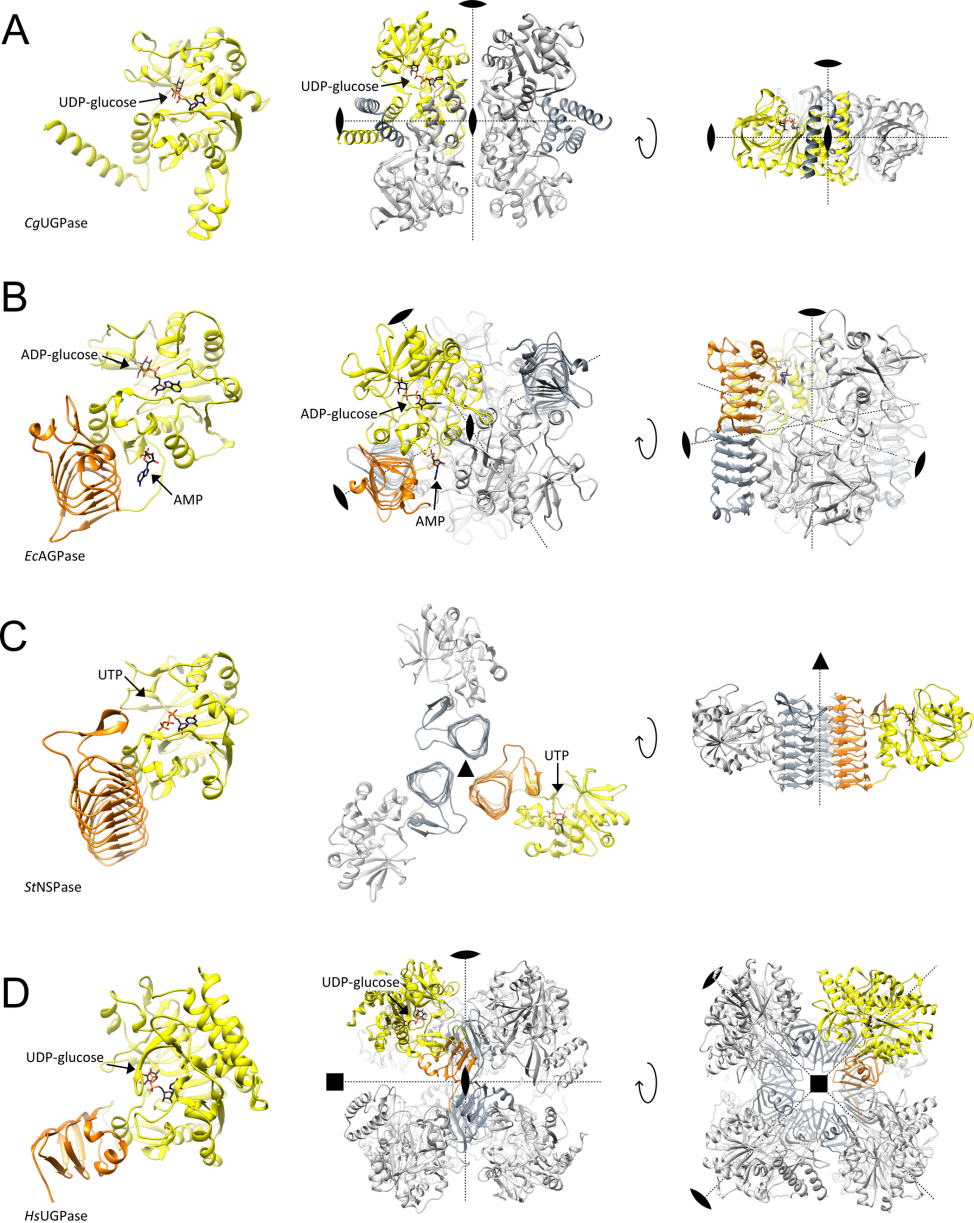
Oligomeric states of NDP-glucose pyrophosphorylases. Selected structures highlight this family's common architecture, presenting from left to right a detail of the protomer and two orthogonal views of the oligomeric assembly. The reference protomer catalytic Rossmann-fold domain is shown in yellow, and when present, the C-terminal LβH domain is in orange. (A) The structure of the UGPase from *Corynebacteria glutamicum* in complex with magnesium and UDP-glucose highlighting the absence of a C-terminal domain and two views of the tetrameric architecture with other protomers in grey (PDB 2PA4).^422^ (B) The structure of *E. coli* AGPase in complex with AMP (PDB 6R8U)^186^ and ADP-glucose by superposition with AGPase from *S. tuberosum* (PDB 1YP4).^404^ On the right, the two views of the tetrameric architecture of *Ec*AGPase show the other three protomers in grey, with the C-terminal domain in dark grey. (C) The archaeal structure of the *Sulfurisphaera tokodaii* glucose 1-phosphate thymidylyltransferase (PDB 5Z09)^424^ in complex with UTP and two views of the trimeric arrangement. Note the longer C-terminal LβH compared to the AGPase, and the difference in the participation in the oligomeric arrangement. (D) The structure of the human UGPase in complex with UDP-glucose (PDB 4R7P).^418^ Note the difference in the relative orientation of the C-terminal domain and its impact in the octameric oligomerization.

NDP sugars in α-glucan metabolism are synthesized by a set of enzymes known as nucleotide-diphosphate-sugar pyrophosphorylases (NSPases), which are metal-dependent nucleotidyl-transferases comprising a catalytic Rossmann fold domain. Two NSPases are mainly implicated in the α-glucan and glucose disaccharide metabolism in bacteria, (i) AGPase (EC: 2.7.7.27) and UGPase (EC: 2.7.7.9), involved in the biosynthesis of ADP-glucose and UDP-glucose, respectively. Both nucleotide sugars differ in the spectrum of reactions they are involved in, with ADP-glucose being specific to α-glucan and some glucose disaccharides in bacteria, archaea, and plants;^61^ whereas UDP-glucose is the commonly used precursor of glycogen in heterotrophic eukaryotes, while also used in glycoprotein, glycolipid, cellulose, callose, sucrose, and trehalose synthesis.^109,389^ Interestingly, other NSPases have the ability of transferring cytidylyl- (EC: 2.7.7.33);^390^ guanidylyl-^303^ and thymidylyl groups (EC: 2.7.7.24),^391^ to glucose 1-phosphate revealing the centrality of nucleotide-activated glucose in the bacterial metabolism (Figures 10 and 11).

The first reported structure of a NSPase family member was that of the *N*-acetylglucosamine 1-phosphate uridyltransferase GlmU from *E. coli*, involved in peptidoglycan synthesis. GlmU protomer is a two-domain protein whose N-terminal domain corresponds to the signature NSPase fold while the C-terminal domain is a Left-Handed β-Helix (LβH).^298^ The NSPase central core domain consists of a Rossmann fold comprising seven strands mixed β-sheet (topology 3214657, underlined antiparallel) decorated by alternating α-helices. NSPase catalytic domains have been previously described as glycosyltransferase A-like (GT-A-like) domains, based on their structural similarity to other members of the GT-A superfamily,^188^ supporting a distant evolutionary relationship. Many NSPases display a C-terminal extension comprised by the LβH fold, belonging to the widespread β-Solenoid fold architecture, particularly the class flat triangular “T-type” cross-section associated with bacterial transferases.^392^ Specifically, the LβH is formed by the stacking of triangular coils comprising successive β-strand linear segments and β-arcs that change the main chain direction in ca. 60°.^393^ Stacked coils form a coordinated network of hydrogen bonds creating β-sheet walls resulting in a triangular prism shape. The internal amino acid side chains are packed to the central axis of the LβH, generating a compact hydrophobic core. Interestingly, the LβH domain of GlmU also comprises a glucosamine-1-phosphate acetyltransferase activity (EC 2.3.1.157) unique in the NSPase family.^394^ It is worth noting that the UDP-*N*-acetylglucosamine 3-*O*-acyltransferase LpxA from *E. coli* (EC: 2.3.1.129)^395^ also comprises an LβH domain involved in catalysis, highlighting the participation of such domains in nucleotide-sugar derivative synthesis. Importantly, in most NSPases the LβH domain presents a different orientation with respect to the GT-A like fold, enabling different forms of oligomerization. LβH oligomerization includes (i) top-to-top dimerization as observed in archeal GDP-mannose pyrophosphorylase,^396^ (ii) base-to-base dimerization allowing tetramerization as observed in the AGPase from *E. coli*,^188^ (iii) side-by-side parallel trimerization as in the case of GlmU from *E. coli*,^298^ and (iv) top-to-top dimerization allowing octamerization as in eukaryotic UGPases.^397,398^

##### 5.1.1.1 ADP-Glucose Pyrophosphorylase

Most bacterial AGPases are characterized as homotetramers (α4), although some species have been reported to exhibit heterotetramers^187,200,399−402^ (Figure 11). In contrast, plant AGPases display heterotetrameric architectures built of small and large subunits (α2β2). However, in plant non-photosynthetic tissues, homotetrameric forms have also been reported.^403^ AGPase regulatory properties emerge from the communication across the tetramer;^61,186,404,405^ therefore, such variability in the AGPase architecture and subunits arrangement accounts for differential regulatory properties in different organisms, highlighting the prominent function as a control enzyme.

Several crystal structures of bacterial AGPases have been reported, including (i) AGPase from *A. tumefaciens* AGPase (*At*AGPase) in complex with sulfate in the allosteric cleft or the allosteric activator pyruvate,^405,406^ (ii) *At*AGPase mutant S72C,^407^ and (iii) *Ec*AGPase in complex with the preferred physiological positive and negative allosteric regulators, FBP and AMP, respectively.^188,408^ More recently, the CryoEM structures of the *Ec*AGPase in complex with FBP and AMP unveiled the allosteric regulatory mechanism of this paradigmatic model.^186^ It is worth noting that the crystal structure of a recombinant truncated homotetrameric form of the small subunit of the AGPase from *Solanum tuberosum* (*St*AGPase) was solved in its unliganded form, and in complex with either ATP (ATP in an off-catalytic position), or ADP-glucose and ADP, highlighting the binding of substrates and products into the active site.^404^ Interestingly, a sulfate ion was found in these structures in positions presumably related to the allosteric inhibitor, phosphate. The small subunit of *St*AGPase is evolutionarily related to the cyanobacterial enzyme, reflected by the high percentage of identity among the protomers.^409^

The N-terminal NSPase catalytic domain contains the active site where ATP and glucose 1-phosphate accommodate. The nucleotide moiety occupies the N-terminal region of the domain, being surrounded by three key loops involved in nucleotide binding, catalysis and allosteric regulation, the nucleotide-binding G-rich loop and the regulatory loops 1 and 2 (RL1 and RL2).^188,404,410^ The G-rich loop is part of an insertion composed of short secondary structure elements, the so-called sensory motif,^188^ that faces both the active and the regulatory sites, comprising key lysine residues involved in catalysis and allosteric regulation (Figure 10). The glucose 1-phosphate moiety binds to a deep pocket located in the C-terminal region of the NSPase domain.^188,298,391,404,411,412^ The C-terminal displays the triangular prism LβH domain arranged in close contact with the GT-A like catalytic domain.^188^

The AGPase protomers build into a physiological and functional homotetrameric structure that can be viewed as a dimer of dimers. The most important contribution to the dimer interface is the triangular base of the LβH prism of each protomer resulting in two antiparallel β sheets.^188,186^ The tetramer assembles mainly by interactions between the N-terminal GT-A-like domains from different dimers, showing a D2 symmetry. Four allosteric sites are located in the corresponding clefts between the GT-A-like and LβH domains of neighboring protomers from different dimers.^186,188^ Specifically, *Ec*AGPase structures in complex with the preferred positive and negative physiological allosteric regulators, FBP and AMP, revealed that the corresponding binding sites partially overlap.^186,188^ The allosteric clefts display a conserved positively charged pocket where a phosphate group of FBP and AMP binds.^404,405,407,408^ As expected, certain residues located in the allosteric cleft, interact differently with the positive and negative regulators, such as Lys39 placed in the sensory motif and associated to the activation mechanism.^203,400,410^ In contrast, Arg130 located in a neighbor protomer enables a stacking interaction with the negative regulator AMP promoting the crosstalk with the RL2 loop in active site. Strikingly, CryoEM structures of *Ec*AGPase in the presence of FBP and AMP, respectively, revealed the activation and inhibition signal transduction mechanisms. The allosteric regulators promote a conformational switch of the regulatory loop RL2, from an AMP-‘locked’ to an FBP-‘free’ state, modulating ATP binding and modulating the enzymatic activity of AGPase.^186,188,408^ Recently, the crystal structure of *At*AGPase was solved in the presence of the positive regulator pyruvate.^406^ Although pyruvate is a weak activator by itself, it synergically enhances the FBP activation.^201^ The homotetrameric *At*AGPase binds two molecules of pyruvate in a planar conformation. The pyruvate binding site is located in a fissure between two contiguous LβH domains of the same dimer.

##### 5.1.1.2 UDP-Glucose Pyrophosphorylase

UDP-glucose was the first nucleotide sugar discovered and arguably the most extended glucose donor in all metabolic pathways.^413,414^ UGPases are present in all kingdoms of life.^389^ UGPase protomers comprise a canonical NSPase domain, with or without a C-terminal LβH domain, showing diverse forms of oligomerization, from monomers^415,416^ to octamers^398,417,418^ (Figures 10 and 11).

Bacterial UGPases, often referred to as GalU/GalF, have been extensively studied, with several structures reported including (i) UGPase from *C. glutamicum* in its unliganded form and in complex with UDP-glucose and Mg^2+^,^419^ (ii) UGPase from *Sphingomonas elodea* in complex with glucose 1-phosphate,^420^ (iii) UGPase from *Helicobacter pylori* in its unliganded form and in complex with UDP-glucose and Mg^2+^,^421^ (iv) UGPase from *Burkholderia ambifaria* in complex with UTP (PDB code 5VE7), and UGPase from *E. coli* in its unliganded form.^422^ Most bacterial UGPases display a NSPase domain and a long C-terminal α-helical hairpin instead of an LβH domain. Two protomers interact by the nucleotide-binding region of the catalytic domain, whereas the corresponding α-helical hairpins hooks each-other dimer, forming a D2 symmetry homotetramer. In contrast, the archeal glucose/glucosamine-1-phosphate uridylyltransferase from *Sulfolobus tokodaii* displays a C-terminal LβH by which the enzyme trimerizes similarly to GlmU.^423,424^ The catalytic site, including the G-rich loop and key catalytic residues, is essentially preserved among UGPases and AGPases, supporting a common mechanism. Major differences are observed in other loops, including the RL2 loop, whose equivalent in bacterial UGPases is shorter, reflecting its participation/specialization in the allosteric regulation mechanism of AGPases. The fact that certain NSPses comprise or not the LβH domain associated with the catalytic domain in different organisms might suggest that, during evolution, the common ancestor of these enzymes acquired the LβH domain before acquiring a high specificity for sugar residues and nucleotides.

#### 5.1.2 The GT-B Fold Enzymes

GTs are classified in families based on the amino acid sequence similarities.^314,425^ GTs utilize a donor substrate containing a substituted phosphate leaving group. The most common sugar donor substrates are nucleotide-diphospho-sugars. There are marked contrasts between the diversity of three-dimensional fold observed for glycoside hydrolases with respect to the limited folds used by GTs. Specifically, only three major folds have been described for GTs, GT-A, GT-B, and GT-C, with some families adopt other unique folds.^312,314,315,426,427^ GT-A and GT-B comprise NDP-sugar-dependent GTs, the so-called Leloir GTs, consisting of two abutting or separated Rossmann fold domains, respectively.^310,314^ Leloir GTs have evolved and diversified outcoming enzymes each specifically selected to construct a particular glycosidic bond. This selection is driven by their specificity toward a defined nucleotide sugar donor scaffold and the acceptor. In the context of a particular sugar motif such as glucose, the pathway that the sugar follows is determined by the activating nucleotidic scaffold, which provides a means for its recognition by the GT, therefore partitioning the sugar motif into different areas of the metabolism. GT-B GTs are the only class present in the α-glucan and glucose disaccharide metabolism, following a retaining catalytic mechanism.^313^ Interestingly, phylogenetic analysis of the GT-B fold families highlights the retaining and inverting mechanisms, which agrees well with root separation in two phylogenetic branches, with few exceptions of inverting GT-B inside the retaining branch,^428^ thus supporting an ancestral relationship between mechanism and enzyme family.

**Figure 12:**
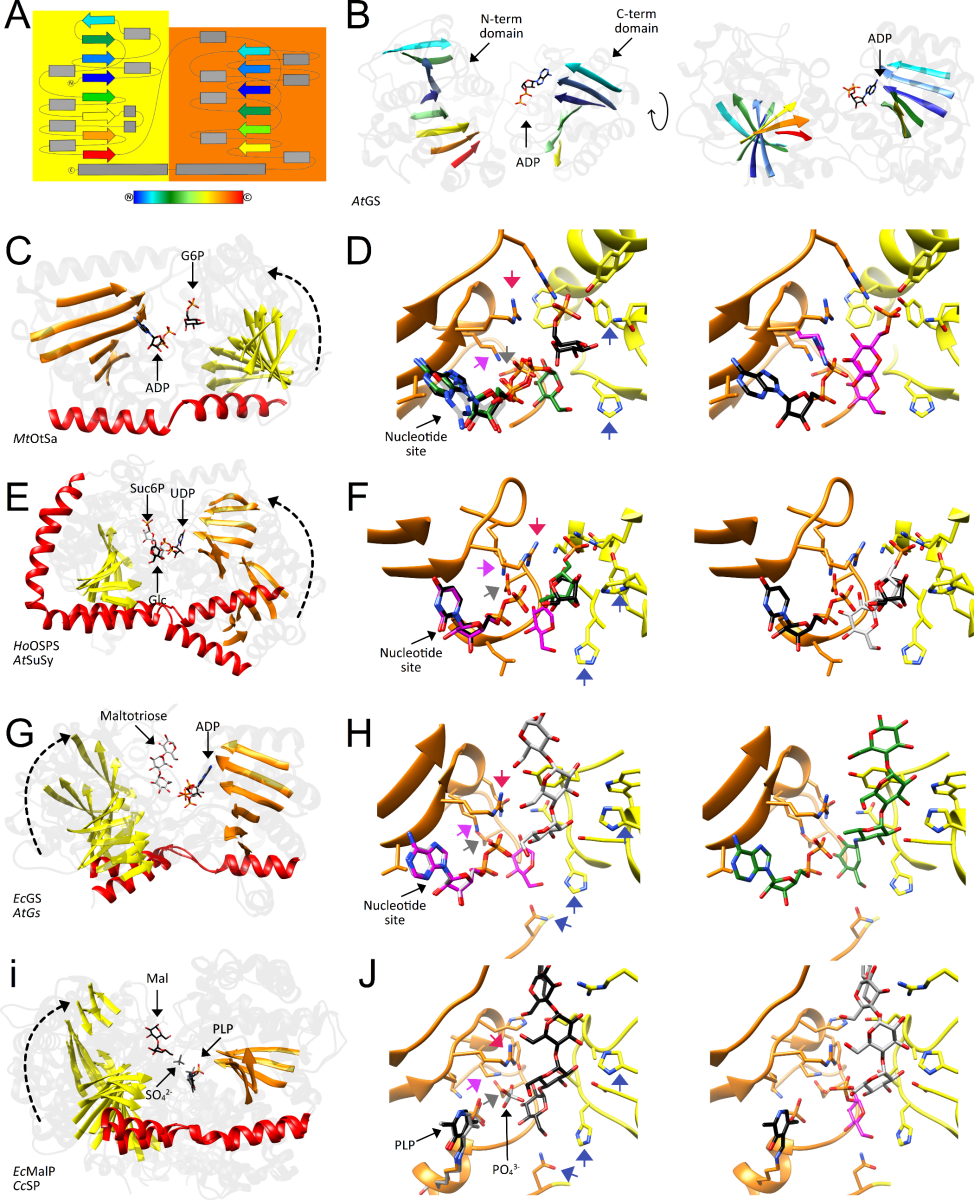
Structure and catalysis of GT-B fold. (A) Representation of the secondary structure of GT-B fold as a diagram showing bars representing α-helices and arrows as β-strands. The domains are highlighted, showing the N-terminal acceptor binding domain (yellow background) and the C-terminal nucleotide binding domain (orange background). For both Rossmann-fold domains, the strands of the central β-sheet are colored independently according to key bar indicating the direction N-terminal C-terminal. Note the C-terminal α-helix completes the N-terminal domain. (B) Two views of the architecture of *A. tumefaciens* glycogen synthase in complex with ADP (PDB 1RZU),^189^ displaying the classic GT-B architecture. (C) Open-to-close motion (indicated by a curve dashed arrow) as observed by the superposition of tructure of *M. thermoresistibile* OtsA *apo* and in complex with ADP and glucose 6-phosphate (PDB 5JIJ and 5JIO).^440^ Both structures central β-sheets are colored (yellow, N-terminal domain, orance, C-terminal domain), the C-terminal α-helix in red, and the rest in semitransparent. Both structures are superimposed on the C-terminal domain to highlight the relative movement of domains. (D) On the right side, the superposition of *M. thermoresistibile* OtsA as reference (PDB 5K44)^440^ with superimposed substrates ADP and glucose 6-phosphate (PDB 5JIO, ligands in black),^440^ ADP-glucose (PDB 5K41, ligand in green),^440^ and GDP-glucose (PDB 5K42, ligand semitransparent).^440^ Note the GDP-glucose nucleotide ring in a displaced position with respect the preferred substrate ADP-glucose. The left panel shows the reference structure *Mt*OtsA in complex with trehalose 6-phosphate (PDB 5K44, ligand in pink)^440^ in the superposition with ADP (PDB 5JIO). (E) Open-to-close motion by the superposition of the structure of *Halothermothrix orenii* sucrose 6-phosphate synthase (SPS) in complex with sucrose 6-phosphate (PDB 2R68)^436^ and its close plant homolog from *A. thaliana* Sucrose Synthase (SuSy) in complex with the UDP-glucose breakdown products UDP and a d-glucosyl derived intermediate (PDB 3S28).^446^ The coloring is equivalent to C, but here both structures are superposed on the N-terminal domain. (F) In the right panel, the superposition of substrates using *At*SuSy as reference in complex with UDP and fructose (PDB 3S27, ligands in pink),^446^ superposed ligands from breakdown product of the UDP-glucose, UDP and the glucosyl hydrolytic derivative (PDB 3S28),^446^ and the HoSPS in complex with fructose 6-phosphate (PDB 2R66, ligand in green).^436^ Note the similar positioning of the sugar substrate in both enzymes. The left panel shows the user the reference structure *At*SuSy the superposition (PDB 3S28, ligands in black)^446^ and the superposition with the *Ho*SPS in complex with the product sucrose 6-phosphate (PDB 2R68, ligands in white).^436^ Note the superposition of the fructose motif between both enzymes. (G) Open-to-close motion by the superposition of the structure of glycogen synthases from *A. tumefaciens* in complex with ADP (PDB 1RZU, ligand in black)^189^ and *E. coli* in complex with maltotriose and ADP (PDB 3CX4, ligands in white).^455^ The same coloring is used to depict the structures; here both structures are superposed on the C-terminal domain showing the sample placement of the nucleotide. Note the coincident position of the nucleotide. (H) In the right panel, the superposition of substrates using *E. coli* as reference GS in complex with ADP-glucose breakdown products ADP and a derived d-glucosyl intermediate (PDB 3GUH, ligands in pink),^456^ the complex with ADP and maltotriose (PDB 3CX4).^455^ The sugar product from ADP-glucose hydrolisis occupies the position suggesting the ready for transference to maltotriose. The left panel shows the use of the same reference structure (PDB 3GUH),^456^ the superposition of ligands from starch synthase from *Cyanobacterium* sp. CLg1 bound to ADP, and the glucan analog acarbose (PDB 6GNF, ligands in green),^450^ mimicking the product post glucosyl transfer. (I) Open-to-close motion by the superposition of the structure of *E. coli* MalP with cofactor PLP in complex with maltose and sulfate (PDB 1AHP, ligands black)^431^ and *Corynebacterium callunae* with PLP cofactor (PDB 2C4M, to be published, ligands black).^670^ Structures are superposed on the C-terminal domain showing the ovelapping placement of PLP. (J) In the right panel, the superposition of substrates using *Ec*MalP as reference with cofactor PLP in complex with pentaoligosaccharide (PDB 1E4O, ligand color black)^468^ and in complex with pentaoligosaccharide (not shown) and phosphate (PDB 1L6I, ligands white).^359^ The phosphate is placed near the position for phosphorolysis to occur. The left panel shows the use of the same reference structure (PDB 1E4O),^468^ the superposition with *Ec*MalP in complex with glucose 1-phosphate (PDB 1L5V, ligands in pink),^359^ and *Ec*MalP in complex with maltotetraose (PDB 2AZD),^469^ showing the location of post reaction products. In panels D, F, H, and J, colored arrows indicate the conservation of conserved residues at the active site. In particular, residues implicated in catalysis are lysine (pink arrow), arginine (red arrow), and glutamic acid (grey arrow). Blue arrows point to conserved residues interacting with glucose residues.

The GT-B fold was first reported for the structure of the rabbit (*Oryctolagus cuniculus*) muscle glycogen phosphorylase (*Oc*GP; EC: 2.4.1.1; GT-35).^196^ The structural arrangement of the two Rossmann fold domains generates an inter-domain cleft where the active site is located.^429^ The crystal and CryoEM structures of GT-B family members revealed that both domains display a high level of flexibility, essential for substrate recognition and catalysis^61,189−191,430−432^ (Figure 12). Although GPs reverse reactions exhibit a retaining mechanism, the main physiological function is phosphorolysis assisted by PLP as a covalently attached cofactor. Interestingly, the first *sensu stricto* reported NDP-sugar-dependent GT-B structure corresponds to the T4 bacteriophage β-glucosyltransferase BGT (EC: 2.4.1.27; GT-63) which transfers glucosidic residues to DNA through an inverting mechanism.^433,434^

GTs involved in the metabolism of α-glucans and glucose disaccharide are classified into five GT-B families, including the GT3, GT4, GT5, GT20, and GT35^425^ (Figure 12). A phylogenetic analysis of the GT-B fold landscape using Hidden Markov Models showed that these five families are clustered together, revealing a close ancestral relationship.^435^ More recently, a deep-learning based study revealed that GT families involved in the metabolism of α-glucan and glucose disaccharide comprise two clusters: (i) GT-B0, includes families GT4, GT5, and GT20, comprises disaccharide synthesis enzymes and the bacterial and plant GSs, and (ii) GT-B1, which consists of GT3 and GT35 enzymes, comprising heterotrophic eukaryote GSs and glucan phosphorylases.^312^

##### 5.1.2.1 Maltose 1-Phosphate Synthase GlgM

**Figure 13:**
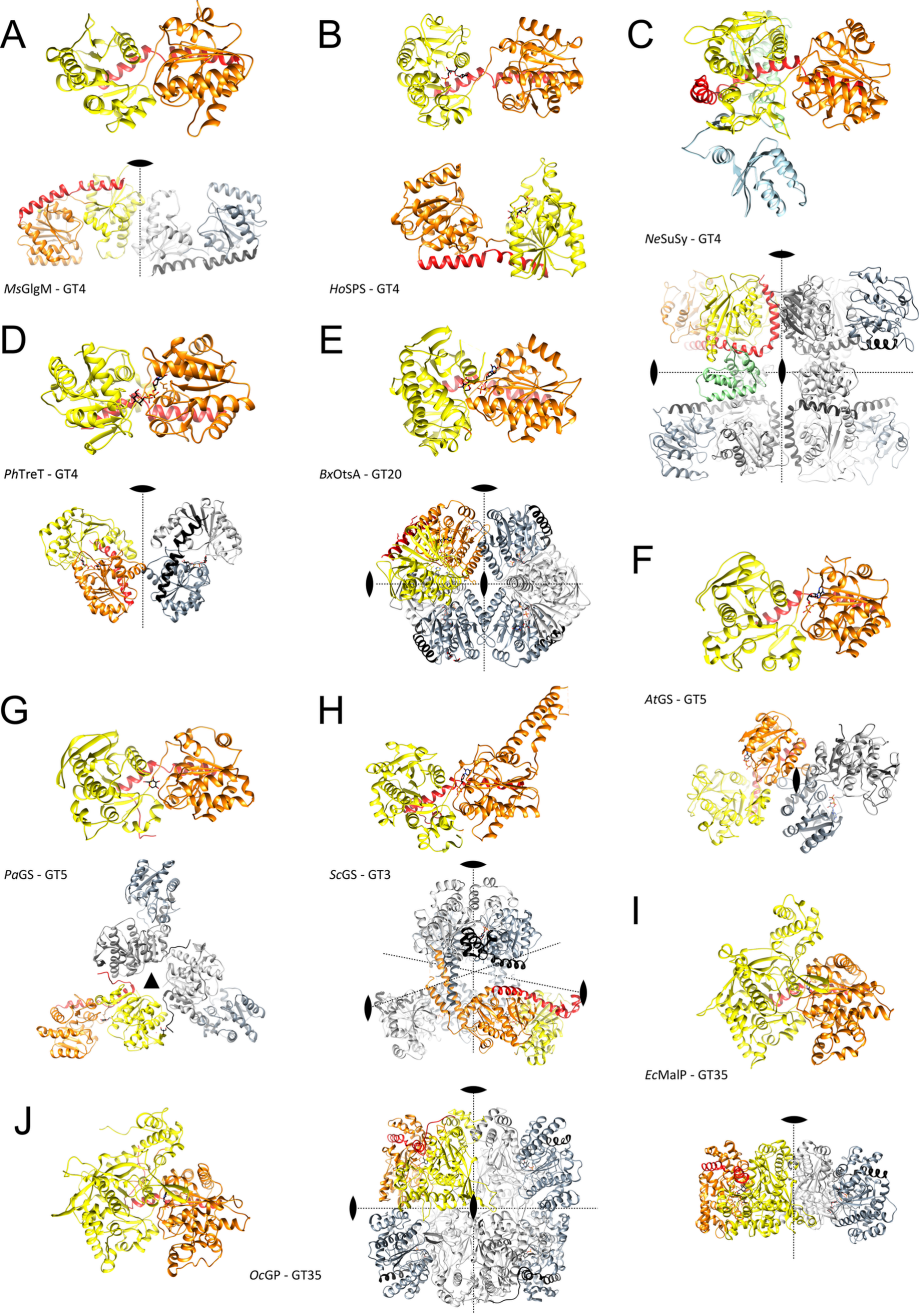
Oligomeric states of GT-B fold enzymes. A gallery of structures and oligomeric states of the GT-B. Selected structures highlight the typical architecture of GT-B enzymes presenting from left to right a detail of the protomer and a view of the oligomeric assembly. In the top of each panel, the reference protomer structure is presented colored in yellow for the N-terminal domain and orange for the C-terminal domain, except for the C-terminal α-helix shown in red. Accessory domains are depicted in different colors. On the bottom of the panel, the oligomeric arrangement with the rest of the protomers is shown in different grey shades. Symmetry axes are shown (oval shape two-fold symmetry, and triangle three-fold symmetry). (A) Structure of *M. smegmatis* α-maltose 1-phosphate synthase GlgM and it dimer highlighting the N-terminal domain oligomerization interaction (PDB 6TVP, GT-4).^233^ (B) Two views of the monomeric structure of *H. orenii* Sucrose Phosphate Synthase (SPS) in complex with sucrose 6-phosphate (PDB 2R68, GT-4).^436^ (C) *N. europaea* Sucrose Synthase SuSy tetramer (PDB 4RBN, GT-4).^444^ Note the oligomerization role of the two N-terminal domains (highlighted in light green and light blue) and the N-terminal domain. (D) Archaeal *P. horikoshii* trehalose synthase TreT and its dimer presenting a C-terminal interaction (PDB 2XA2, GT-4).^269^ (E) Structure of *Burkholderia xenovorans* OtsA tetramer (PDB 5VOT, to be published, GT-20).^671^ The tetrameric oligomerization involves both N- and C-terminal domains. (G) Structure of the *A. tumefaciens* GS (PDB 1RZU, GT-5).^189^ The dimeric form appears in the crystal structure, possibly representing a physiological assembly. F. Archeal *P. abyssi* GS and trimeric assembly mediated by N-terminal interactions (PDB 3FRO, GT-5).^458^ (H) Eukariotic *S. cerevisiae* GS in basal state in complex with UDP and tetramer (PDB 3O3C, GT-3).^432^ Note the long α-helix at the C-terminal domain providing interaction for the oligomerization. (I) *Ec*MalP an its dimer (PDB 1AHP, GT-35),^431^ the oligomerization mediated by N-terminal with an α-helical insertion playing a prominent role. (J) Eukaryotic *O. cuniculus* GP (PDB 9GPB, GT-35).^672^ Note the high similitude between *Ec*MalP protomer and dimerization with the *Oc*GP tetramer when considered as dimers of dimers.

The α-maltose 1-phosphate synthase GlgM belongs to the GT4 family (Figure 13A). As described earlier, GlgM uses ADP-glucose and glucose-1P as the main substrates (EC: 2.4.1.342).^119^ GlgM also uses UDP-glucose as a donor with glucose 1-phosphate. However, it is less efficient. Surprisingly, UDP-glucose is not used as a donor when glycogen is used as the acceptor, further restricting the ability of the enzyme to generate glycogen.^119,224^ The crystal structure of GlgM from *M. smegmatis* is determined in its unliganded form.^233^ GlgM comprises a homodimer in which three parallel α-helices from the N-terminal domain of one subunit interact in an antiparallel fashion with the equivalent helices in a noncrystallographic two-fold-related subunit to give a six-helix bundle. The structural comparison of GlgM with bacterial GSs (GT-5) revealed that the C-terminal domains architecture are essentially preserved, with several residues participating in ADP-glucose binding essentially conserved. In contrast, the N-terminal domains show more significant differences in secondary structure. The central parallel β-sheet is two strands shorter in GlgM relative to the bacterial GlgA GSs, a characteristic also observed in other GT4 family members including sucrose phosphate synthase.^233,436^ The fact that GlgM is mostly present in Actinobacteria,^119^ and not present in other GlgE containing bacteria such as Chlamydiales,^222^ raises the question about the evolutive origin of GlgM, either by speciation from other GT-B disaccharide synthase or a reduction of function from an ancestral GS.

##### 5.1.2.2 Trehalose 6-Phosphate Synthase OtsA and Trehalose Synthase TreT

Two groups of bacterial GT-B enzymes account for the synthesis of the α,α-trehalose scaffold using either glucose 6-phosphate or α-glucose as acceptors, the so-called trehalose 6-phosphate synthase (T6PS; OstA) or trehalose synthase (TS; TreT), respectively (Figure 12 and 13).

T6PSs belong to the GT20 family, comprising two activities, UDP-glucose-dependent (EC 2.4.1.15) and ADP-glucose-dependent enzymes (EC: 2.4.1.347). T6PSs have been extensively studied, with several structures reported including (i) OtsA from *E. coli* in its unliganded form and in complex with either UDP and glucose 6-phosphate, UDP-glucose, UDP-2-deoxy-2-fluoroglucose, or a glycomimetic inhibitor,^437−439^ (ii) OtsA from *S. venezuelae* in its unliganded form, a GDP-glucose dependent enzyme,^303^ and (iii) OtsA from *Mycobacterium thermoresistibile* in its unliganded form and in complex with either ADP and glucose 6-phosphate, ADP-glucose, GDP-glucose, glucose 6-phosphate, trehalose 6-phosphate, trehalose, or ADP and fructose 6-phosphate.^440^ Interestingly, thermophilic bacteria and archaea TreTs (EC: 2.4.1.245) belong to the GT4 family and use several NDP-glucose donors with similar efficiency, including ADP-glucose, UDP-glucose, and GDP-glucose.^441,442^ Two archeal TreT three dimensional structures have been experimentally determined by X-ray crystallography, including (i) TreT from *P. horikoshii* in its unliganded form and in complex with either UDP, or UDP-glucose,^269^ and (ii) TreT from *Thermoproteus uzoniensis* in complex with either UDP-glucose or trehalose.^443^

Although OtsA and TreT show a conserved GT-B fold, comparison of the individual protomers highlights important structural differences due mainly to the presence of additional β-strands flanking the central β-sheet and insertions in the N-terminal domain of the GT20 family, regions involved in OtsA tetramerization. In addition, OtsA from *M. thermoresistibile* complexes with ADP-glucose or GDP-glucose highlight subtle differences in the binding site that explains the preference for ADP-glucose.^440^ Interestingly, three single-point mutations allow the conversion of the donor substrate preference to UDP-glucose.

##### 5.1.2.3 Sucrose 6-Phosphate Synthase and Sucrose Synthase

There are two highly related enzymes implicated in the synthesis of sucrose in bacteria, the sucrose synthase (SuSy; EC 2.4.1.13) and the sucrose-phosphate synthase (SPS; EC: 2.4.1.14), both belonging to the GT4 family (Figures 12 and 13). There are several three dimensional structures experimentally determined up to date, including (i) SuSy from the chemolithoautotroph *Nitrosomonas europaea* in its unliganded form,^444^ (ii) the halophilic anaerobic bacteria *Halothermothrix orenii* SPS in its unliganded form and in complex with fructose 6-phosphate,^436^ and (iii) the SPS from the cyanobacteria *Thermosynechococcus elongatus* in its unliganded form and in complex with hydrolyzed UDP-glucose.^445^ In addition, the homologous plant enzyme SuSy-1 from *Arabidopsis thaliana* was reported in complexes with UDP and fructose.^446^ It is worth noting that SuSy enzymes can use sucrose to reversibly produce the nucleotide-sugar *de novo*, linked to starch biosynthesis in heterotrophic tissues of plants,^447^ and other polysaccharides in cyanobacteria.^329^ Interestingly, cyanobacteria SPSs can use UDP-glucose and ADP-glucose as substrates.^47,448^

Most of the reported structures display a single canonical GT-B fold; nevertheless, SuSys form *A. thaliana* and *N. europaea* display two additional contiguous N-terminal domains, so-called sucrose synthase (SSN). Notably, the SSN-1 accessory domain comprises α-helices and four β-strands whereas SSN2 is formed by a five α-helices bundle, involved in enzyme tetramerization. Besides these differences, the structural superposition of all SuSy and SPS enzymes discloses a common binding site to accommodate the substrates sucrose and NDP-glucose.

##### 5.1.2.4 Bacterial Glycogen Synthase

Compared to disaccharide synthases, GSs are more sophisticated enzymes capable of processive catalysis by associating with the nascent α-glucan and performing rounds of glycosyl-transferand α(1→4)-glucan polymerization.^109,449^ Prokaryotic GSs (GlgA) belong to the GT5 family and comprise three subgroups according to the nucleotide-sugar donor specificity, including (i) UDP-glucose (EC: 2.4.1.11), (ii) ADP-glucose (EC: 2.4.1.21), and (iii) UDP-glucose/ADP-glucose (EC: 2.4.1.11/EC: 2.4.1.21). In addition, plant GSs, so-called starch synthases (EC: 2.4.1.21), are also classified in the GT5 family, resembling bacterial GS, particularly close to cyanobacterial GSs.^450^ On the other hand, heterotrophic eukaryote GSs (EC: 2.4.1.11) are classified into the GT3 family, preserving the overall architecture of the catalytic core when compared with the bacterial and plant homologues, but displaying important insertions of secondary structural elements.^61,192,432,451^ This architecture enables the necessary conformational changes for allosteric regulation, such as the activation by glucose 6-phosphate and inhibition by covalent phosphorylation^109,452^ also facilitating the interaction with glycogenin^190,191,453,454^ (Figures 12 and 13).

Several bacterial three dimensional structures of full-length GSs have been experimentally determined by X-ray crystallography, revealing catalytic and substrate specificity aspects, including (i) GS from *A. tumefaciens* in its unliganded form and in complex with ADP,^189^ (ii) GS from *E. coli* in complex with ADP and glucose, ADP, and oligosaccharides.^455,456^ In addition, the granule bound starch synthase CLg1GBSS from *Cyanobacterium* sp. was reported in complex with ADP and acarbose.^450^ Furthermore, crystal structures of archeal GS from *Pyrococcus abyssi* were solved in its unliganded form and in complex with 1,5-anhydro-d-fructose, and bound to an α(1→4)-glucan in a non-catalytic glycogen-binding site.^449,457,458^ There is limited information about the oligomeric state of bacterial GSs, with early reports pointing to the existence of GS from *E. coli* as dimers, trimers, and tetramers.^459^ Nevertheless, reported structures on GS from *E. coli* revealed only monomeric forms, whereas the asymmetric crystal unit points to a possible dimer of GS from *A. tumefaciens*. Interestingly, crystal structures and electron microscopy studies on archeal GS from *P. abyssi* revealed functional trimerization mediated by the N-terminal domains.^457^ Heterotrophic eukaryote GSs of the GT3 family revealed additional α-helices in the GT-B fold core allowing functional tetramerization.^61,109,432^ All structural studies on GSs from the GT3 and GT5 families support the occurrence of an open-to-close movement critical during catalysis and regulatory mechanisms.^61,189−192,432,451,454,456^

##### 5.1.2.5 Glycogen Phosphorylase and Maltodextrine Phosphorylase

From its elucidation,^460,461^ eukaryotic glycogen phosphorylase (GP) is one of the most studied enzymes with ca. 250 structures currently deposited in the Protein Data Bank (PDB; https://www.rcsb.org/). Nevertheless, these enzymes, belonging to the GT35 family, have been structurally studied to a lesser extent in the case of prokaryotes. There are two homologous α(1→4)-glucan phosphorylases in bacteria: (i) the maltose-inducible maltodextrin phosphorylase MalP, dedicated to the processing of environmental extracellular α-glucans as a source of carbon and energy and having a reduced activity against glycogen,^462−464^ and (ii) the glycogen phosphorylase GP (GlgP) specialized in the recovery of glucose from intracellular glycogen storages.^462,465,466^

The structural analysis of these bacterial enzymes relies mostly on the reported crystal structures of MalP from *E. coli* due to the lack of a *bonafide* experimentally determined bacterial GP structure. The crystal structures comprise (i) MalP from *E. coli* in complex with either maltose, acarbose, tetra- or penta-thio-oligosaccharides and phosphate, glucose 1-phosphate, glucose 1-phosphate, and maltopentaose;^359,431,467-468,469^ (ii) the starch phosphorylase from *Corynebacterium callunae* (PDB code, 2C4M, to be published); and (iii) the α(1→4)-glucan phosphorylase from *S. mutans*. The typical two Rossmann-fold domains GT-B architecture comprises a central catalytic cleft with the C-terminal domain containing the covalently attached PLP prosthetic group site^198,359,431,467−469^ (Figures 12 and 13).

As in other GT-B enzymes, the active site is located in a deep cleft between the N- and C-terminal domains.^198,431^ PLP lies covalently attached to C-terminal domain lysine residue facing the catalytic center (K646 in MalP from *E. coli*), whereas the α(1→4)-glucan substrate binds mainly to the N-terminal domain, which in the ‘close’ conformation forms a channel between both domains.^359^ This channel imposes a minimum length for the linear oligosaccharide to reach the catalytic center, correlating with the reduction of glycogen chains up to four glucose residues from the branch point.^359^ In MalP from *E. coli*, three highly conserved lysine residues, K534, K540, and K554, coordinate the PLP phosphate group and the arrangement of the phosphate moiety toward the reactive glycosidic bond. All the structures on MalP from *E. coli* support a model in which substrate binding and product release require the occurrence of open-to-close motion.^198,359,431,467−469^

#### 5.1.3 Rossmann-like HAD Domain Disaccharide Phosphatases

**Figure 14:**
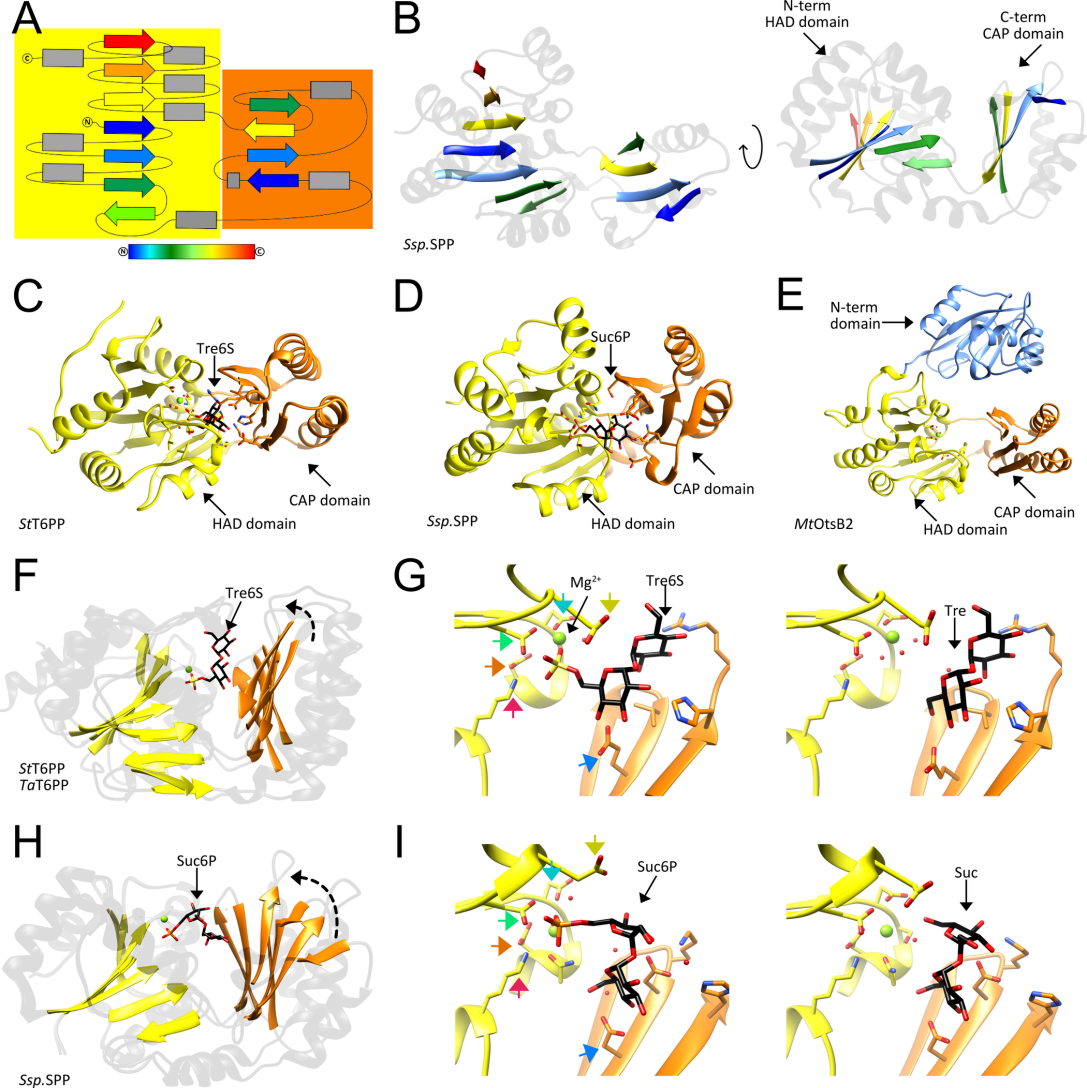
Structure and catalysis of HAD-fold phosphatases. (A) The secondary structure of HAD phosphatases is represented as a diagram showing bars representing α-helices and arrows as β-strands. The domains are highlighted to show the N- and C-terminal Rossmann-like folds, in yellow and orange background correspondingly. Arrows of the central β-sheet are colored according to the color key bar indicating the direction N-terminal-to-C-terminal. (B) Two views of the architecture of the *Synechocystis* sp. sucrose phosphatase (SPP) (PDB 1S2O)^352^ presenting the canonical HAD phosphatase two domain arrangement, where the β-sheets of both Rossmann-like folds are independently colored from the direction N-terminal C-terminal. (C) The structure of *S. typhimurium* trehalose 6-phosphate phosphatase (StT6PP) in complex with trehalose 6-sulfate (Tre6S) and Mg^2+^ showing the classical two domain architecture of the catalytic fold HAD phosphatases. (D) Structure of the *Synechocystis* sp. sucrose-phosphatase (SPP) in complex with sucrose 6-phosphate (PDB 1U2T).^352^ (E) Structure of the trehalose 6-phosphate phosphatase (OtsB2) from *M. tuberculosis* (PDB 5GVX),^349^ showing the N-terminal accessory domain in light blue. Note the strictly conserved architecture of the *St*T6PP and OtsB2 catalytic fold and the subtle differences with SPP. (F) The open-to-close motion of the HAD domain phosphatases (indicated by a curve dashed arrow) as observed by the superposition of the structure of *Se*T6PP in complex with Tre6S and Mg^2+^ (PDB 6UPC)^350^ and the *T. acidophilum* T6PPb in complex with Mg^2+^ (PDB 1U02).^473^ Both structures have been superposed by their N-terminal domain β-sheets (yellow). (G) In the left panel, detail of the active site of *St*T6PP in complex with the substrate analog Tre6S and Mg^2+^ (PDB 6UPC)^350^ representing the coordination of the substrate pre-reaction. In the right panel, StT6PP in complex with the product trehalose (PDB 6UPC).^350^ (H) Similar to F, the open-to-close motion by superposition of *Synechocystis* sp. SPP. F. in complex with Mg^2+^ (PDB 1S2O)^352^ and in complex with sucrose 6-phosphate (PDB 1U2T).^352^ (I) In the left panel, detail of the active site of *Synechocystis* sp. SPP in complex with the substrate sucrose 6-phosphate (PDB 1U2T), and in the right panel with the product sucrose (PDB 1TJ4).^352^ In F and H, conserved residues interacting with substrates and implicated in catalysis are indicated by corresponding colored arrows.

Haloacid dehydrogenase superfamily (HAD) comprises a diverse group of enzymes that catalyze the cleavage of substrate C-Cl, P-C, and P-OP bonds, including different substrate specificities and activities, such hydrolases, ATPases, isomerases, transferases, and different types of phosphatases.^470^ All HAD enzymes comprise (i) a catalytic core Rossmann-like fold domain comprising a central parallel β-sheet (topology 32145) surrounded by α-helices^354,355^ and (ii) a small CAP domain responsible for the diversification of substrate specificity within the family.^471^ The two domains face each other and are separated by a cleft, with the enzyme adopting both open and closed forms that facilitate substrate recognition and product release^472^ (Figure 14).

##### 5.1.3.1 Trehalose 6-phosphate phosphatase OtsB2

Several crystal structures of bacterial trehalose 6-phosphate phosphatases (T6PP; OtsB2) provide insights into substrate specificity and catalytic mechanism of trehalose dephosphorylation, including (i) T6PP from *Salmonella enterica* in its unliganded form and in complex with either Mg^2+^, trehalose 6-sulfate and Mg^2+^, and trehalose and Mg^2+^,^350^ (ii) T6PP from *Burkholderia pseudomallei* in complex with Mg^2+^,^351^ (iii) T6PP from *M. tuberculosis* in complex with trehalose 6-sulfate and Mg^2+^,^349^ and (iv) an archeal T6PP from *T. acidophilum* in complex with glycerol and Mg^2+^ (Figure 14).^473^ The HAD-like domain displays the Rossmann-like domain with a central β-sheet which is extended with a β-hairpin motif forming a β-meander before the connection with the CAP-domain. The CAP domain shows a characteristic 2-layer sandwich α–β plaits. Interestingly, OtsB2 from *M. tuberculosis* comprises an extra N-terminal domain connected to the HAD-like domain by a long loop, remaining adjacent to the active site.^349^ The N-terminal domain truncation results in 70% loss of the enzymatic activity. T6PP complexes with trehalose 6-sulfate and trehalose show the disaccharide bound into the cleft in an extended conformation, oriented by charged residues from both the HAD-like and the CAP domains.^349,350^ As presented before in Section 4.3.3, the catalytic mechanism of TPP, as other HAD phosphatases, involves the formation of a covalent enzyme-substrate intermediate through the nucleophilic attack of a conserved aspartate, assisted by a lysine, on the phosphorus atom from the substrate. This nucleophilic residue belongs to long loop emerging from the HAD-domain first β-strand contains a DxDx(T/V) motif participating in the coordination of the divalent metal cation Mg^2+^. Moreover, the active site, resembling that of other HAD phosphatases, includes other two aspartic acids which coordinate Mg^2+^ for dephosphorylation.^349^

##### 5.1.3.2 Sucrose 6-phosphate phosphatase

To date, the sucrose 6-phosphate phosphatase (S6PP) from cyanobacterial *Synechocystis sp.* is the only reported enzyme of this class.^352,474^ This enzyme has been extensively studied including experimental structural data of its unliganded form and complexes with sucrose, sucrose 6-phosphate, sucrose and phosphate, glucose, trehalose, cellobiose, and maltose.^352,474^ S6PPs share the structural fold observed for T6PPs, showing a high degree of similarity with the corresponding HAD-like and CAP domains. Nevertheless, subtle changes can be observed including a short α-helix in the transition between the HAD-like and CAP domains that replaces the last β-sheet in T6PPs. S6PPs present a shorter loop emerging from the HAD-domain first β-strand that account for a more open cleft, compared to T6PPs. The S6PP complexes with sucrose and sucrose 6-phosphate indicate that this loop is not required to position the disaccharide into the substrate binding site since the sucrose arranges in a different orientation when compared with trehalose 6-phosphate in T6PP.^352,474^ The glucose moiety of sucrose binds buried into the cleft plays a critical role in substrate recognition and specificity. Supporting this notion, the affinity for the glucose moiety accounts for inhibition by sucrose and glucose.^352,474^ The structural and biochemical data support a common catalytic mechanism for S6PPs and T6PPs mediated by the nucleophilic attack of the substrate to form a covalent phospho-enzyme intermediate.

#### 5.3.4 Maltokinase Mak/Pep2

Currently, several crystal structures of the mycobacterial enzyme have been reported, including Mak/Pep2 from *M. tuberculosis* in its *apo* form and in complex with maltose,^375^ Mak/Pep2 from *Mycobacterium vanbaalenii* in its *apo* form, and in complex with ATP, and the non-hydrolizable ATP analog AppCp,^228^ and Mak/Pep2 from *M. smegmatis*, as a component of the Mak/Pep2-TreS complex.^232^ The structure of Mak/Pep2 is curious in the context of other enzyme folds participating in the α-glucan metabolism. Mak/Pep2 resembles the bacterial 5-methylthioribose kinase, despite low global amino acid sequence identity,^228^ displaying many conserved structural motifs associated with nucleotide binding and enzymatic activity.^228,475^

**Figure 15:**
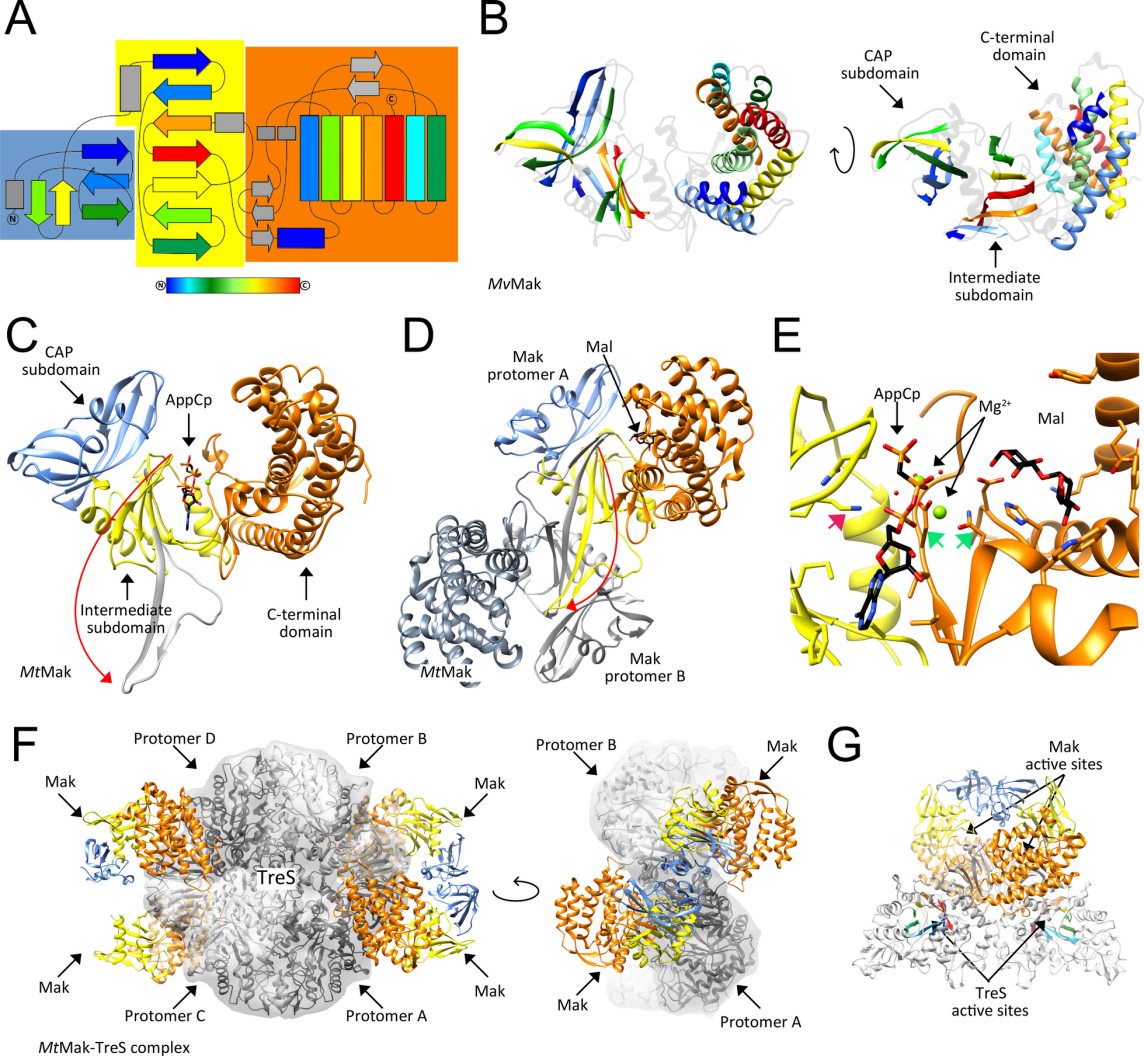
Structure and catalysis of Maltokinase. (A) Representation of the secondary structure of Maltokinase showing bars representing α-helices and arrows as β-strands. The background colors indicate the CAP domain (blue), the intermediate domain (yellow), and the C-terminal domain (orange). Arrows of the central β-sheet and the C-terminal α-helices are colored according to the color key bar indicating the direction N- to C-terminal. (B) Two views of the architecture of MaK/Pep2 from *M. vanbaalenii*. The secondary structure elements are colored according to the scheme shown in panel A (PDB 4U98).^228^ (C) Comparison of the maltokinase protomer by superposition of *Mv*Mak in complex with ATP analog AppCp (PDB 4U98, colors)^228^ and *M. tuberculosis* MaK (PDB 4O7P, grey)^375^ showing the alternative intermediate domain β-sheet difference (red arrow) between monomeric and dimeric maltokinases. (D) Structure of the dimer of *Mt*MaK complexed with maltose presents the reference protomer in colors and the second protomer in grey (PDB 4O7P).^375^ The intermediate domain swapped β-sheet is indicated with the arrow. (E) Detail of the active site by superposition of *Mv*Mak in complex with ATP analog AppCp (PDB 4U98)^228^ and maltose from *Mt*MaK complex (PDB 4O7P).^375^ (F) Two views of the octameric complex of *M. smegmatis* TreS with Mak showing the central TreS tetramer in grey. (G) Detail of the *Ms*TreS-Mak complex highlighting the active site of both enzymes located on distant protomers reveals the impossibility of substrate channeling between sites.

The mycobacterial Mak/Pep2 structures reveal a two-domain fold architecture comprising the N-terminal CAP subdomain, containing three antiparallel β-strands that form a curved β-sheet and enclosing the N-terminal α-helix and a β-hairpin. A second intermediate subdomain contains a central seven-stranded antiparallel β-sheet having two lateral α-helical segments. Finally, the C-terminal domain is composed of two central four α-helical bundles, a β-hairpin, and a two-stranded β-sheets^375,228^ (Figure 15). Both N- and C-terminal domains remain strongly bound by salt bridges and hydrogen bonds. The active site of Mak/Pep2 enzymes is located within a deep pocket between the intermediate subdomain and the C-terminal domain. Reported structural complexes in the presence of ATP and non-hydrolizable analogs clearly define the site where the adenine group locates. Importantly, a strictly conserved Lys149 interacts directly with the α-phosphate, and the γ-phosphate is hold by the so-called ‘P-loop’ residue Ser136. Mg^2+^ sites comprise side chains of Gln310 and Asp322, making bridges with ATP phosphates. On the other hand, maltose accommodates mainly into a pocket located in the C-terminal domain.^375^

The N-terminal CAP subdomain presents large B-factors, suggesting conformational flexibility.^228^ The possible structural/biological function of this subdomain is regulation as well as representing an anchoring point to TreS.^231,232^ It is worth noting that Mak/Pep2 and TreS form a hetero-octameric complex.^231,232^ Although the crystal structure of the Mak/Pep2:TreS complex appears to rule out substrate channeling between the corresponding active sites,^231^ the structural arrangement possibly enhances the enzymes recruitment facilitating the succession of isomerization (TreS) and phosphorylation (Mak/Pep2) reactions to transform trehalose into maltose 1-phosphate. In addition, formation of the complex may provide a mean for allosteric effects, as observed for similar complexes.^232,476^ Specifically, the Mak/Pep2:TreS complex has an overall diamond-shape, where the TreS tetramer forms the core. Each TreS protomer pairs with a Pep2/Mak protomers at the apices of the tetramer resulting in a 4 + 4 configuration,^232^ mainly mediated by interactions with the C-terminal domain of TreS and Mak/Pep2 N-terminal CAP subdomain. Interestingly, both enzymes are part of a single polypeptide in some members of the phylum Chlamydiae,^222^ although presenting a monomeric form might present a similar relative arrangement.

### 5.2 TIM Barrel Fold Enzymes (TBF) and α-Glucan Metabolism

The TIM-barrel is a versatile scaffold present in many enzymes involved in pathways of the central metabolism.^477^ TIM is an acronym for the glycolytic enzyme triose-phosphate isomerase, the first reported crystal structure for the scaffold.^478^ TIMs are α/β proteins displaying an (α/β)_8_ topology. Specifically, the central barrel core comprises eight parallel β-strands. Each β-strand interacts with a corresponding α-helix in antiparallel orientation surrounding the barrel core. Overall, α-helices and β-strands display an angle with the β-barrel central axis,^479^ whereas the overall fold shows a degree of elliptical shape.^480^

TIM barrel fold (TBF) structures revealed a toroidal shape with pseudo-C8-fold symmetry that defines two faces, where the catalytic face is oriented at the C-terminal end of the β-strands.^479^ As a result of the overall directionality of the β-barrel and α-helices, TIM barrel domains have a global dipole across the toroid, although it does not correlate with the location of the active site.^481,482^ In addition to the important amino acid sequence differences between TBF enzymes, the overall shape dictates the conservation of intrinsic domain dynamics.^483^ Moreover, TBF enzyme families, such as GHs, exhibit preserved long-range electrostatic interactions among charged residues directed toward the barrel center and the interaction energy in the structural residue network.^483^ The clustering of TBF enzymes according to geometrical characteristics such as the length of helices and sheets and the location of the elliptical axes indicates that GH α-amylases (GH13) have commonalities with distant enzymes including glycolytic enzymes such as enolase and pyruvate kinase.^480^

As a result of the relevance of α-glucans in biology and the evolutionary versatility of these enzymes, TBF α-glucan processing enzymes present a large number of functions in terms of substrate specificity and α-glycosidic bond reactions.^484,485^ Several of these functions are markedly improved by accessory domains such as Carbohydrate Binding Modules (CBM), which themselves comprise a large diversity.^486^ These modules involved in the polymer binding contribute to enzyme-polymer heterogeneous catalysis, as stated by the Sabatier principle, meaning enzymes interact with substrates with intermediary strength, permitting binding association-dissociation kinetics are optimal to permit effective catalysis.^487,488^ The double inversion mechanism is central to all activities, and the reaction can be either under thermodynamic or kinetic control.^246,313,340^ For example, the comparison of debranching enzymes architecture and accessory domains belonging to very different TBF GH-fold families reveals considerable differences,^489^ whereas debranching/branching enzymes can be clustered inside the same GH subfamily with the seemingly same structure.^235,490,491^ Furthermore, it has been proven that subtle differences as a single point mutation can account for changes in function.^492^ Since function may hold for structural extreme differences, but nuances can also alter the outcome function, it is challenging to extract a rationale using a reductionist approach. With this in mind, in the following sections, we present the overall makeup of GH families’ structural organization for selected functions. Later, we present a parallel comparison of TBF-substrate complexes to highlight commonalities in enzyme-metabolite coevolution for these enzymes.^378^

#### 5.2.1 TIM-Barrel α-Glucan GH13 Family

TBF enzymes classified into the GH13 family comprise the most significant number of specificities related to α-glucan metabolism.^246,340,493^ The GH13 family is one of the most studied from a structural point of view, and it is further subdivided into several subfamilies. Structures corresponding to selected functions associated to glycogen and disaccharide metabolism include (i) 4-α-glucanotransferase (EC 2.4.1.25);^289^ (ii) GlgE α(1→4)-glucan phosphate α-maltosyltransferase (EC 2.4.99.16);^216−219,221,494^ (iii) GlgB glycogen branching enzyme (EC 2.4.1.18); (iv) amylosucrase (EC 2.4.1.4),^156,332,333,495−497^ sucrose phosphorylase (EC 2.4.1.7);^362,498−500^ (v) sucrose 6-phosphate phosphorylase (EC 2.4.1.329);^501^ (vi) AmyA α-amylase (EC 3.2.1.1);^502−505^ (vii) GlgX glycogen debranching enzyme (EC:3.2.1.196);^235^ and (viii) malto-oligosyltrehalose trehalohydrolase TreZ (EC 3.2.1.141),^348^ trehalose synthase TreS (EC 5.4.99.16),^231,369^ and malto-oligosyltrehalose synthase TreY (EC 5.4.99.15).^506^

**Figure 16:**
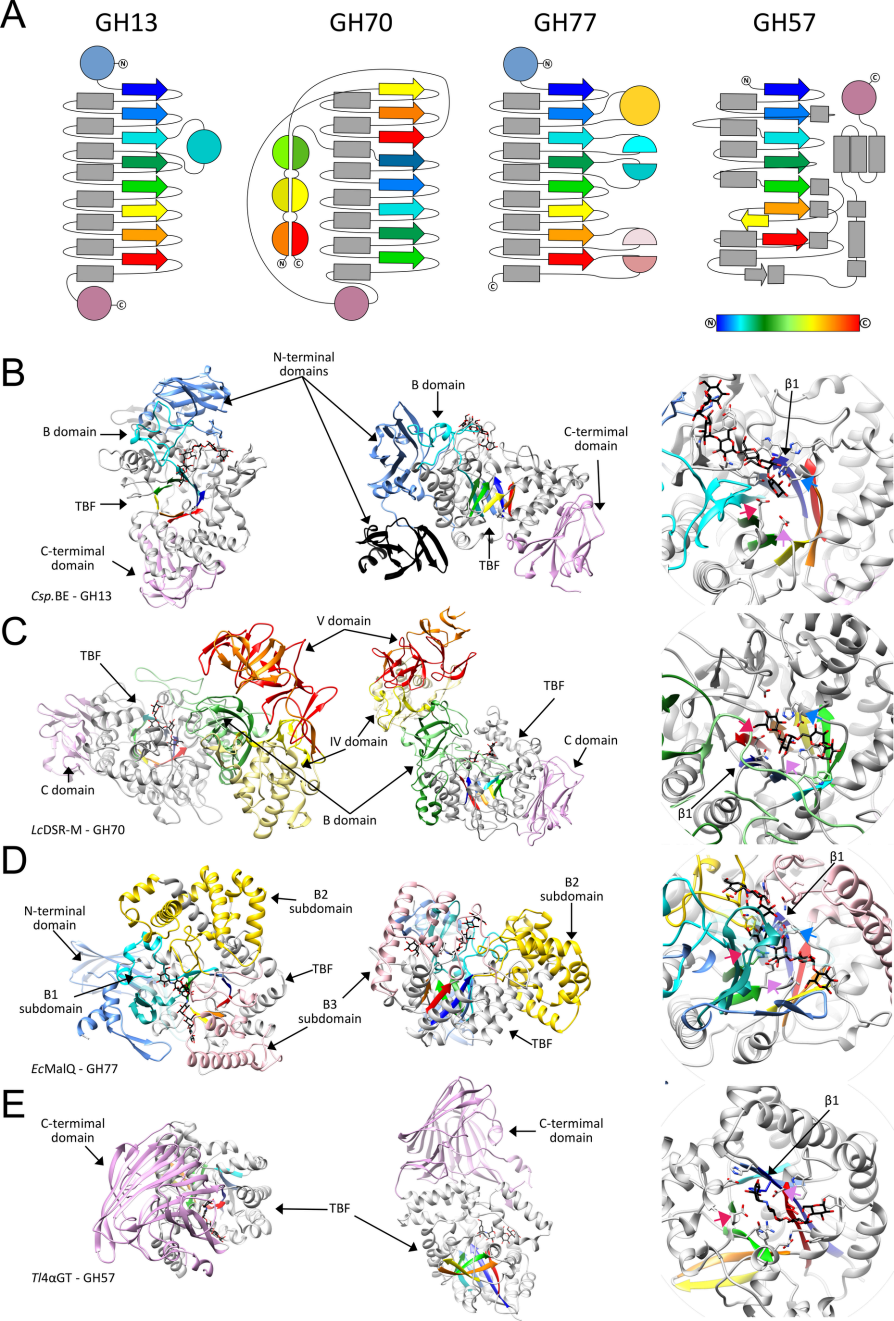
Structure and catalysis of TIM barrel fold enzymes. (A) The secondary structure of TBF enzyme families is represented as a diagram showing bars representing α-helices and arrows as strands. From left to right, the scheme offers the four GH families present in the α-glucan metabolism, GH13, GH70, GH77, and GH57. Strands in the (α/β)_8_-barrel are highlighted in colors according to N- and C-terminal color key. For the GH13, accessory domains are shown as colored circles, including the N-terminal domain (blue), inserted β3-strand α3-helix domain B (cyan), and the C-terminal β-sandwich domain C (maroon). The GH70 architecture presents three associated domains formed by N-terminal and C-terminal not consecutive segments shown as semicircles called domain V (orange and red), domain IV (dark and bright yellow) and domain B (dark and light green), and domain C inserted between α6 and β6 (maroon). Note the alternative arrangement of (α/β)_8_ barrel starting with an α-helix instead of a β-strand as GH13. GH77 scheme shows an N-terminal domain (blue circle) before the catalytic domain, whereas the (β/α)_8_ barrel presents subdomains inserted. Subdomain B2 is indicated by a yellow circle, whereas the subdomains B1 (cyan) and B3 (pink) are formed by two semicircles. Note for the GH77 the lack of the C-terminal domain C compared to the GH13 family. The GH57 scheme shows the incomplete TBF (β/α)_7_ displaying several secondary elements extra associated with the catalytic fold. Note the β-strands colored with the same color scheme, presenting seven in the barrel and an antiparallel β-strand not belonging to the barrel (yellow). (B) From left to right, two views of the structure of a GH13 branching enzyme from *Cyanothece* sp. in complex with maltohexaose (PDB 5GQV),^673^ and in the left panel, a close-up of the TBF active site. The view shows domains colored according to the scheme presented in A. Note an extra N-terminal domain colored in black. The close-up of the GH13 catalytic site indicates the position of strand β1 as a reference, with the rest of the barrel strand numbering increasing counter-clockwise. Catalytic triad residues are indicated in emerging loops extensions in strand β4, aspartate nucleophile (magenta arrow), in β5 glutamate acid/base (pink arrow), and in β7 aspartic stabilizer (blue arrow). (C) Same as in A, but the structure of GH70 *L. citreum* dextransucrase DSR-M inactive mutant N-terminal truncated mutant E715Q in complex with isomaltotetraose (PDB 6HTV).^512^ The catalytic domain permutation places the catalytic triad in different C-terminal loop strands in the barrel. Note a glutamine indicated by the pink arrow appears in the position of the acid/base due to the corresponding mutation E715Q. (D) Similar for the structure of the GH77 *E. coli* amylomaltase MalQ in complex with the pseudo-heptasaccharide (PDB 4S3R).^366^ (E) Structure of the GH57 *Thermococcus litoralis* 4-α-glucanotransferase complexed with acarbose (PDB 1K1Y).^522^ From B to E, note the close-up of the left panel; the catalytic domain is oriented to place the bound polysaccharide in the same overall orientation.

The canonical GH13 enzyme comprises a characteristic TBF (β/α)_8_ barrel domain (A) including the catalytic center of the enzyme and an accessory domain (B) inserted into the loop between the β3-strand and α3-helix of the barrel, whereas a third C-terminal domain (C) completes the enzyme core architecture.^507,508^ The GH13 presents a TBF with a linear order of secondary structures, with a topology N-β1-α1-β2-α2-β3-α3-β4-α4-β5-α5-β6-α6-β7-α7-β8-α8-C. The B-domain presents a variable structure and length, whereas the C domain is formed by a β-sandwich domain. In addition to the core fold, often one or two additional C-terminal domains, D and E, are present.^493^ The E domain is characterized as a starch-binding domain. These domains correlate with GH13 function, specificity, and taxonomy^493^ (Figure 16).

#### 5.2.2 The Alternative TBF GH70 Family

The GH70 family comprises a number of enzymes that use sucrose as a sugar donor to synthesize α-glucans, so-called glucansucrases (GSuc), oftentimes catalyzing more than one type of glucosyl transfer. Selected activities include (i) α(1→6)-dextransucrases (EC 2.4.1.5),^509−512^ α(1→3)/ α(1→6)-alternansucrase (EC 2.4.1.140),^338,513^ and the α(1→4)/α(1→6)-reuteransucrase (EC 2.4.1.-).^514^

GSuc enzymes are usually large extracellular proteins that consist of four different regions, including (i) a signal peptide, (ii) an N-terminal variable region (VR) containing other repeat units, (iii) the TBF catalytic domain, and (iv) a C-terminal glucan-binding domain (GBD) also comprising repeating units. GH70s have a characteristic circularly permuted TBF compared to the GH13 topology.^126^ The GH70 (α/β)_8_ barrel starts with an α-helix that corresponds to α3 in the GH13 (instead of starting in β1), in a relative order N-α3-β4-α4-β5-α5-β6-α6-β7-α7-β8-α8-β1-α1-β2-α2-β3-C. Nevertheless, these enzymes still present the same catalytic amino acid motifs of the GH13 family enzymes, with TBF sequence position accounting for the circular permutation. In addition, crystal structures of truncated GH70 GSuc enzymes reveal five domains in a U-shaped form, with equivalent A, B, and C domains as observed in the GH13 members making the catalytic core, and domains IV and V are unique to GH70 enzymes.^126^ Interestingly, domain B is composed of two discontinuous inserts and is next to domain A, while domain C is a single domain formed by the N- and C-termini and is involved in substrate binding. Domain IV is involved in oligomerization, whereas domain V is located at the C-terminus and is believed to be involved in glucan binding^126^ (Figure 16).

#### 5.2.3 The TBF GH13-like GH77 Family

Reported bacterial crystal structures of the GH77 family members are limited to the 4-α-glucanotransferase (EC 2.4.1.25).^341,366,515−517^ The GH77 TBF has a similar topology to the GH13 (N-β1-α1-β2-α2-β3-α3-β4-α4-β5-α5-β6-α6-β7-α7-β8-α8-C), reflecting a high evolutionary connection.^518^ Nevertheless, the separated classification family is supported by the divergence in several structural factors.^485,519^ The core of the GH77 enzymes comprises the TBF with three insertions, resulting in the formation of three subdomains so-called B1, B2, and B3. The B1 and B3 subdomains correspond to domains B and C in the GH13 family. The B2 domain is unique to the GH77 family, possibly playing a role in substrate recognition. In addition, the B3 subdomain does not present the classic β-sandwich as in domain C of GH13 enzymes. This structural arrangement of accessory domains generates a narrower and deeper catalytic cleft compared to GH13 enzymes, where domains clamp the α-glucan substrates. Moreover, although the catalytic triad is conserved, some GH77 enzymes present differences in the residue environment at the catalytic site.^520^ Finally, some enzymes in this family present an additional N-terminal immunoglobulin-like fold domain presumably implicated in α-glucan binding^519^ (Figure 16).

#### 5.2.4 The Incomplete TBF GH57 Family

The GH57 family comprises an expanding number of activities since the discovery of α-amylases with unrelated sequences to the GH13 family.^274,521^ There are a limited number of bacterial and archeal GH57 enzymes with known three dimensional structures associated to the α-glucan metabolism: the 4-α-glucanotransferase (EC 2.4.1.25)^522^ and the glycogen branching enzyme (EC 2.4.1.18).^281,364^ The reported enzymes comprise an N-terminal incomplete TBF, containing a short helix (often called subdomain B) emerging from β2, an intermediate five-helix domain, and a C-terminal β-super sandwich domain.^523^ The incomplete TBF has a (β/α)_7_ topology including a β-barrel with a distorted toroidal shape. The β6 does not participate in the barrel, being located in an antiparallel orientation with respect to β7. Therefore, the lack of a strand in the β-barrel generates a more open conformation because there are two gaps between β3 and β4 and between β7 and β8.^523^ As in the other GHs, the TBF C-terminal β-strands define the active site, displaying a more significant loop variability, including the insertion of additional secondary elements (Figure 16).

## 6 Polysaccharides at the Dawn of Life

How polymers metabolism has emerged is a central question in biology. In the case α-glucans, their ubiquitous presence in present biology suggests that they have deep evolutionary roots, possibly dating back to the earliest life forms or before. Miller and Urey, whose famous experiment supported the prebiotic chemical origin of life, proposed that polymerization may be catalyzed by adsorption on clay and mineral surfaces.^524^ Orgel and colleagues proposed abiotic polymerization of amino acids and nucleotides on mineral surfaces, suggesting that these rocky interphases could provide a “library” for molecular evolution.^525,526^ Polysaccharides may arise first among other polymers in prebiotic times, suggesting that they were present in a glycoworld from which the RNA world emerged.^527,528^ Furthermore, it has been speculated that high-energy poly-phosphates may be served as a scaffold for the assembly of major polymers,^527,529^ thus facilitating the condensation of the first polysaccharides.

The putative occurrence of neutral polysaccharides, such as glucans, might help in original phase separation in the primordial soup, allowing the concentration of other charged proto-biopolymers and coalescence of dispersed particles.^528^ Indeed, carbohydrate polymers may appear on prebiotic earth as a prerequisite for life’s subsequent development.^527^ Long and colleagues showed that dextrans can contribute in creating compartments with properties of synthetic cells.^530^ Although lessons and the latest advances from synthetic chemistry pin-point first principles for synthesizing α-glucans,^531,532^ it appears that further exploration of α-glucan prebiotic origin is needed, which potentially could offer fascinating views. The presumed existence of prebiotic glucose, as well as the demonstrated abiotic phosphorylation of glucose forming α-d-glucose 1-phosphate,^533,534^ opens the door to think about the fate of these sugars phosphates in abiotic times.

### 6.1 Extracellular Polysaccharides and Biofilms at the Origin of Living Cells

The evolution of organisms selected confirmed metabolic processes taking advantage of available compounds and synthesizing others that benefit survival under environmental pressure in a defined niche. α-Glucan metabolism provides a framework for understanding how physiological functions associated with cellular structure and energy storage exhibited in different bacterial lifestyles. Biofilms seem to be the most frequent lifestyle, with ca. 80% of bacterial and archaeal cells living in this form, which may serve as diversity incubators; planktonic lifestyle might be a secondary form.^535,536^ Biofilms provide functions such as harvest and access to nutrients, shelter protection against environmental stresses, water retention to maintain extracellular enzyme activities, social cooperation, and horizontal gene transfer.^537,538^ The occurrence of biofilm structures appears early in the fossil record (ca. 3.2 giga annum; Ga), a characteristic shared by prokaryote ‘living fossils’ present in hot springs and sea hydrothermal vents;^539^ bacteria and archaea lineages emerged at a similar time frame (ca. 3.4 Ga).^540^ Diverse glucans have been shown to form biofilms. β-Glucans, such as cellulose^541,542^ and β-(1→3)-glucans,^543^ are common biofilm-forming exopolysaccharides. α-Glucans^119,544−548^ are components of the extracellular matrix, capsules and biofilms, often linked to glycogen metabolism. Interestingly, hot spring-living *Geobacillus tepidamans* forms a glucan-containing biofilm,^549^ whereas *Geobacillus* sp. expresses an extracellular thermostable (4→6)-α-glucanotransferase.^550^ Moreover, biofilm-forming uncultivated SM1 Euryarchaeon isolated from subsurface biotopes of sulfidic springs can synthesize glycogen.^551^ Based on these pieces of evidence, the presence of α-glucans in ancestral biofilms can be conjectured.

### 6.2 Considerations on the Origin of Glycogen Metabolism in Bacteria

The emergence of metabolic systems and their evolution represents a central question in biochemistry and biology. Efforts are dedicated to reconstruct the metabolic make-up of the Last Universal Common Ancestor (LUCA), the hypothetical prokaryotic common organism antecedent to all existing life. Inferring gene repertoires arising billions of years ago is extraordinarily challenging since orthologous genes/proteins are subject to continuous vertical inheritance, gene loss, and horizontal gene transfer.^552^ Nevertheless, it could be argued that primitive α-glucan pathways appeared early in the gene makeup of organisms due to the importance of survival to bacteria.^94^ Supporting this notion, glycogen metabolism provides an advantage in enduring famine, resisting different abiotic stresses and helping in colonizing niches.^82,95,173,544,553^ The structure of bacterial glycogen with a small average chain degrades slowly and serves as a resilience mechanism, prolonging survival.^101^ Finally, glycogen plays a role in awakening bacteria from dormancy, a near-dead state.^554^ Therefore, glycogen metabolism could have played a role in the early stages of life evolution.

From the point of view of synthetic biology, it has been proposed that the construction of small bacterial genomes containing a minimal set of proteins deduced from existing genomes can reveal the essential set of survival genes compatible with cellular life,^555^ thus strongly suggesting a requisite gene repertoire in the LUCA. Nevertheless, the metabolic routes are strongly context-dependent, and it seems difficult to find protein-coding genes that are entirely conserved in all prokaryotic genomes.^556^ The minimal genome synthetic approach appears unsuitable for evaluating early glycogen metabolism since it is not essential in some bacteria.^557,558^ Interestingly, the presence of GBEs and GDEs in all kingdoms of life indicates glycogen metabolism is an early process;^559^ while GP has also been predicted to be part of the LUCA.^560^ Furthermore, meta-consensus analysis of metabolic pathways predicted early enzyme functions and properties that may exist in the core metabolism of early life forms, with several enzymes related to starch metabolism.^561^

The LUCA has been predicted/proposed to be an anaerobe.^562−564^ Several strict anaerobes are known to produce glycogen,^565,566^ while glycogen metabolism enzymes were identified in anaerobic hyperthermophile bacteria from the genera *Thermotogales* and *Aquifex*.^567,568^ The reconstruction of the tree of life also suggests that the LUCA was a thermophile,^569^ which is consistent with the hypothesis of the origin of life at hydrothermal vents.^570^*Thermotogales* and *Aquifex* are at the base of the bacterial clade^571^ and therefore are thought to resemble ancient bacteria. In addition, the anaerobic thermophilic archaea *Methanocaldococcus jannaschii* shares a metabolism similar to bacteria, comprising genes of the glycogen metabolism,^572^ supporting the hypothesis that α-glucan metabolism predates separation between archaea and bacteria. Interestingly, symbiosis between nanohaloarchaeon and haloarchaeon is based on utilization of different polysaccharides including glycogen.^263^ The giant tube-worm hydrothermal vents-living *Riftia pachyptila*, utilizes glycogen as the main anaerobic substrate during extended anaerobiosis and a key molecule in chemoautotrophic endosymbionts,^573^ suggesting glycogen plays relevant roles in extreme niches associated with life origin. Altogether, these views support glycogen metabolism as an ancient process.

### 6.3 The α-Glucan Pathways in the Light of Metabolism Evolution Models

**Figure 17:**
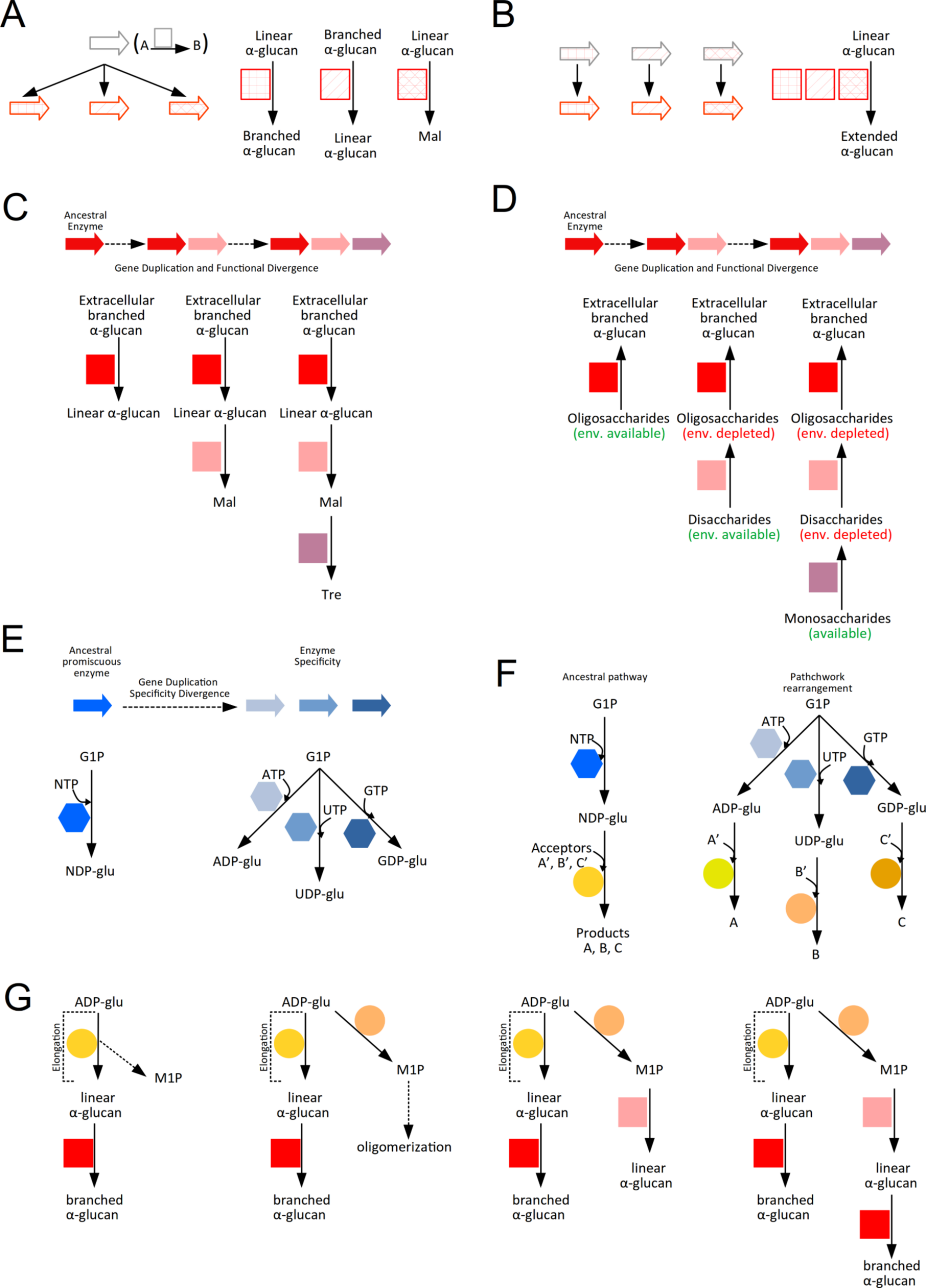
Evolutionary basis of α-glucan chemistry and metabolic pathway diversity. Schematics depict an exercise based on different aspects and models for the evolution of metabolic pathways onto α-glucan metabolism. (A) Hypothetical scenario for the divergent evolution of one gene into three enzymes with different functions. Considering organisms with a coding gene (grey arrow) for an enzyme responsible for processing an α-glucoside A into B. Due to gene cumulative mutations and selection by environmental pressure (in the same or different organisms) the enzyme gene diverges into three distinct gene/enzymes (red arrows and boxes), with a different active site suited for different cellular functions involving α-glucosyl bond processing. Over time, these mutations became fixed in the population, creating three distinct enzyme lineages. This case may reflect the diversification of the GH13 family into different functions. (B) Hypothetical case of convergent evolution, involving three different species of bacteria that live in similar environments where the processing of an α-glucan provides an adaptive advantage to survive. Each species had evolved independently and contained genes (grey arrows with different red hatching) that do not share a recent common ancestor, encoding enzymes responsible for processing unrelated α-glucosides. The selection of mutations in these three genes converges on enzymes with the same function. This case may reflect the convergence of enzymes from GH13, GH77, and GH57 families into enzymes 4-α-glucanotransferase (EC 2.4.1.25). (C) A speculative scenario of an anterograde evolution pathway^581^ in which an organism utilizes a branched α-glucans using a gene/enzyme (red arrow and box) that processes the substrate in linear chains to be able to harvest it. An event of gene duplication and posterior divergence results in a second gene/enzyme (pink arrow and box) that processes the linear α-glucan into maltose, providing an advantage due, i.e., cluster to the same operon for utilization of nutrients. The same process repeats to acquire a gene/enzyme (purple) to transform maltose into trehalose, reaching a more efficient way to produce the functional disaccharide. (D) Speculative scenario of retrograde evolution^575^ where an extracellular α-glucan is critical for survival, which can be produced by an organism gene/enzyme (red arrow and box) from oligosaccharides obtained from the environment. An undergoing depletion of the disaccharide generates the selection, under growing pressure, of a new gene/enzyme (pink arrow and box) originated by gene duplication and divergence, that can synthesize oligosaccharides from disaccharides available in the environment. The same process repeats to acquire a gene/enzyme (purple) to generate disaccharides from monosaccharides. (E) A hypothetical case of an ancestral gene encoding promiscuous enzyme (blue arrow and hexagon),^582,583^ i.e., an NSPase produces different sugar donors NDP-glucose. The enzyme through gene duplication and subsequent divergence evolves into three differentiated enzymes (light, medium, and dark blue arrow and hexagons) with higher specificity to synthesize ADP-glucose, UDP-glucose, and GDP-glucose. (F) The differentiation of promiscuous enzymes recruited in an ancestral pathway (blue hexagon, and yellow circle) under gene duplication and subsequent divergence can lead to the arrangement of specific enzymes into different pathways “patchwork”. (G) Hypothetical enzyme-substrate co-evolution involving an enzyme (yellow circle) that synthesizes a linear α-glucan in a pathway comprising a second enzyme (red box) to generate branched α-glucans. Eventual errors or promiscuity lead the first enzyme to generate the byproduct maltose 1-phosphate. In a second moment, the new metabolite scaffolds the evolution of a specific enzyme (salmon circle). A higher level of maltose 1-phosphate is removed by unspecific enzymes by oligomerization. In a third moment, the enzyme (pink box) is recruited and evolves to catalyze such reaction specifically.

From the putative LUCA metabolism, enzymatic networks have diversified into apparently disparate systems. Several models for the evolution of pathways attempt to explain the emergence of metabolic networks on prebiotic and ancestral cellular metabolism.^574^ In this regard, the α-glucan and glucose disaccharide metabolism present a framework that could be evaluated *a priori* by these models while subjected to a simple chemical scaffold. Hereof, we discuss the emergence of the α-glucan biosynthetic machinery based on these models (Figure 17).

The pioneer retrograde model proposed by Horowitz, based on the Oparine-Haldane “primordial soup” hypothesis,^570^ proposed the metabolic evolution of a rudimentary heterotrophic organism under significant selective pressure due to the depletion of the prebiotic compound supply.^575^ Under such circumstances, the primordial cells acquired mechanisms to synthesize functional molecules by assembling the enzymatic machinery in a retrograde manner, originating the metabolism that frees them from exogenous sources of such compounds. In the retrograde view, α-glucans metabolism could arise by harvesting hypothetical proto-glucan compounds as an energy source or making part of functional compartments/biofilm in primordial cell communities. Once depleted, selective pressure enforced the selection of synthetic machinery to obtain in a retrograde manner the enzymes to synthesize first the α-glucan polymers or disaccharides, then nucleotide sugars, and so on. Phylogenetic studies comparing *E. coli* enzymes and pathways universally present in all domains of life indicate that the catabolism of α-glucans is among the most conserved pathways with enzymes present in Archaea, Bacteria, and Eucarya.^576^ Moreover, the presence of an analogous enzymatic machinery to the glycogen synthesis for the usage of external α-glucan, such as the maltose/maltodextrin utilization system present in distant bacteria,^464,577−579^ could indicate that the evolution of the α-glucan metabolism first associated to external sources. The presumed LUCA containing α(1→4)-glucan-phosphorylases is part of the GT35 family (MalP/GP).^560^ The reversibility of these enzymes^360,580^ insinuates an ancient pivot function between synthesis and degradation. During evolution, PLP could be incorporated into a proto-GT-35 NDP-sugar-dependent enzyme, surrogating a nucleoside monophosphate, and resulting in MalP/GP.^314^ This appears as a sensible explanation in the context of almost the whole GT-B family using nucleotide-activated sugars.

Contrary to the retrograde model, Granick proposed that biochemical pathways extended in an anterograde fashion, acquiring successive steps in the metabolic pathway to produce a functional metabolite which provides a survival advantage.^581^ Exopolysaccharides may be originated from early communities to form a matrix of biofilms as a survival strategy, allowing colonization of surfaces. From this point of view, the metabolism could have extended from gluconeogenesis toward α-glucans synthesis, possibly supported by activities from the nucleotide and RNA metabolism based on meta-consensus enzyme functions (EC: 2.4.1.- and 2.7.7.-).^561^ The putative LUCA presents NSPases and related GT-2 enzymes, including UTP-glucose 1-phosphate thymidylyltransferase.^560,563^ Moreover, phylogenetic analysis highlights GT folds go back in evolution to the LUCA.^428^ Therefore, this view could support nucleotide-sugars and disaccharides first, instead of a retrograde view of glucans first.

In the evolution of pathways, innovations are the driving force for enzyme function specialization that permits the synthesis of new specific metabolites. Ycas proposed earlier enzymes were less specific (more promiscuous) to catalyze several related reactions with a smaller number of enzymes.^582^ Independently, and along the same reasoning, Jensen postulated a “Recruitment Hypothesis” in which pathway assembly occurs by recruitment of such primitive enzymes with a wide range of chemically related substrates, also known as the “Patchwork Hypothesis”.^583^ The evolution of an enzyme into diverse enzymes with specific traits arising from gene duplication of the ancestral enzyme can be viewed as functional specialization, or division of labor, an evolutionary mechanism observed from molecules to organisms.^584^ Promiscuity outcome includes a ‘Flexible Metabolome’, a concept that suggests genetic and metabolic pathways are cross-wired in diverse unpredicted forms, therefore inherently ambiguous and stochastic.^585^ Importantly, enzyme promiscuity appears widespread in modern α-glucan metabolism. NSPases and GT-B enzymes often present some degree of usage of alternative nucleotide triphosphate or nucleotide sugars, respectively. For example, (i) OtsA *M. thermoresistibile* can use ADP-glucose and GDP-glucose,^440^ and (ii) GS from *P. abyssi* can use UDP-glucose and ADP-glucose.^457^ Moreover, NSPase promiscuity has been reported in several bacterial enzymes, including the AGPase from *Rhodococcus jostii* that can use ATP, GTP, and CTP, as well as diverse hexose 1-phosphates.^586^ On the other hand, TIM barrel folds bear a large diversity of functions for α-glucan processing. In the case of GBEs and GDEs (GH13), both are related to gene duplication and paralogous.^559^ Furthermore, functional and phylogenetic studies show the majority of these enzymes classified into the GH13 family comprising 35 subfamilies likely monofunctional, while others appear polyspecific,^246^ which correlates with the promiscuity to specificity evolution. Moreover, the GH13 family is a member of the clan GH-H, which also contains GH70 (glucansucrases) and GH77 (amylomaltases) families, supporting the evolutionary relatedness of these enzymes.^518^

### 6.4 Structural Relationships between the Enzyme Folds Involved in α-Glucan Biosynthetic Pathways to Other Metabolic Pathways

The catalytic folds involved in α-glucan and glucose disaccharide synthesis are widespread in the other areas of the metabolism, which could be thought to represent distant evolutionary relationships or convergent evolution. Rossmann-like folds are thought to have evolved prior to LUCA from a primordial generic nucleotide-binding domain, which participates in five out of eight enzymatic reactions of the oldest metabolic Wood-Ljungdahl pathway.^380,563^ TIM barrels have been hypothesized to allow early evolution of protein-mediated catalysis since they are flexible to incorporate different cofactors, providing a scaffold to transition from ribozymes and peptides catalysts to modern protein enzymes.^587^ Rossmann-like folds and TIM barrels can be considered the oldest folds based on the hypothesis that protein folds are more prevalent and more widely shared the more ancestral they are.^377^

In the case of the NSPase fold, the N-terminal region of the active site, participating in the nucleotide-binding, shows high degree of similarity to enzymes in distant regions of the metabolism, including (i) the molybdopterin guanine dinucleotide synthesis enzyme MobA, a key component in molybdenum cofactor synthesis,^588,589^ and (ii) the bifunctional methylerythritol 2,4-cyclodiphosphate synthase of *Campylobacter jejuni*, which transfers cytidil- groups to 2-C-methyl-d-erythritol 4-phosphate to form CDP-ME2P in the isoprenoid biosynthesis.^590^ Interestingly, the C-terminal pocket portion accounting for the sugar-binding in the NSPase folds presents the largest variability which correlates with differences in the sugar substrate, including (i) the *N*-acetylmuramic acid α-1-phosphate uridylyltransferase MurU (EC: 2.7.7.99) involved in peptidoglycan metabolism,^591^ and (ii) the nucleotide monophosphate CMP-Kdo synthetase (EC: 2.7.7.38), that activates the modified sugar Kdo for the synthesis of bacterial cell wall lipopolysaccharides.^592^ Altogether, the N-terminal region presents variability associated with nucleotide usage, whereas the C-terminal region of the cavity highlights a broad variability associated to several other areas of the metabolism. Finally, the NSPase domain shares similarities to enzymes with other functions. Interestingly, the N-terminal eukaryotic translation initiation factor eIF2B presents an extraordinary degree of architectural similitude.^593^

The GT-B fold, comprising the two articulated Rossmann domains, represents an interesting case since several bi-substrate reactions can profit from the open-to-close mechanism. Therefore, this architecture could have emerged by convergent evolution or very distant divergent evolution in several areas of the metabolism.^380^ Indeed, a resembling GT-B-like structural organization is observed in non-GT-B enzymes. Many examples can be extracted from glycolytic enzymes such as (i) the phosphofructokinase that catalyzes the ATP-dependent phosphorylation of fructose 6-phosphate to fructose 1,6-bisphosphate (EC: 2.7.1.11),^594^ (ii) the phosphoglycerate mutase that catalyzes the transfer of phosphate from 3-phosphoglycerate acid to form 2-phosphoglycerate (5.4.2.12),^595^ and (iii) the phosphoglycerate kinase (EC: 2.7.2.3) that catalyzes the reversible transfer of a phosphate group from 1,3-bisphosphoglycerate to ADP producing 3-phosphoglycerate and ATP.^596^ Other examples include (i) UDP-glucose 6-dehydrogenase that converts UDP-glucose into UDP-glucuronic acid, the precursor in the biosynthesis of bacterial exopolysaccharides (EC: 1.1.1.22),^597^ and (ii) 3-d-phosphoglycerate dehydrogenase that converts d-3-phosphoglycerate to phosphohydroxypyruvate in the serine synthesis pathway (EC: 1.1.1.95).^598^

The TIM barrel fold is the scaffold of at least 15 different enzymatic functions in all life kingdoms.^477,599^ Network interaction-conservation phylogenetic analysis allows classification of different groups of TIM barrel fold enzymes, including remote homologues, highlighting structural and functional evolutionary relationships.^479^ Due to the relationship of the α-glucan to the glycolysis pathways, it is worth mentioning three glycolytic enzymes comprising this fold, (i) the triosephosphate isomerase that interconverts dihydroxyacetone phosphate and d-glyceraldehyde 3-phosphate (EC 5.3.1.1),^600^ (ii) the pyruvate kinase that catalyzes the final step of glycolysis producing ATP and pyruvate from ADP and phosphoenolpyruvate (PK, EC: 2.7.1.40),^601^ and (iii) the 2-Keto-3-deoxy-6-phosphogluconate (KDPG) aldolase that transforms KDPG into pyruvate and d-glyceraldehyde-3-phosphate (EC 4.1.2.14) in the alternative glycolysis Entner–Doudoroff pathway.^602^ Other remarkable enzymes harboring this fold in other parts of the metabolism are (i) the α-subunit of the tryptophan synthase (EC: 4.2.1.20),^603^ and (ii) the iron-sulfur-flavoprotein trimethylamine dehydrogenase (EC: 1.5.8.2) part of an electron transfer complex.^604^

### 6.5 The Big Picture of Bacterial α-Glucan and Glucose Disaccharide Metabolisms

**Figure 18:**
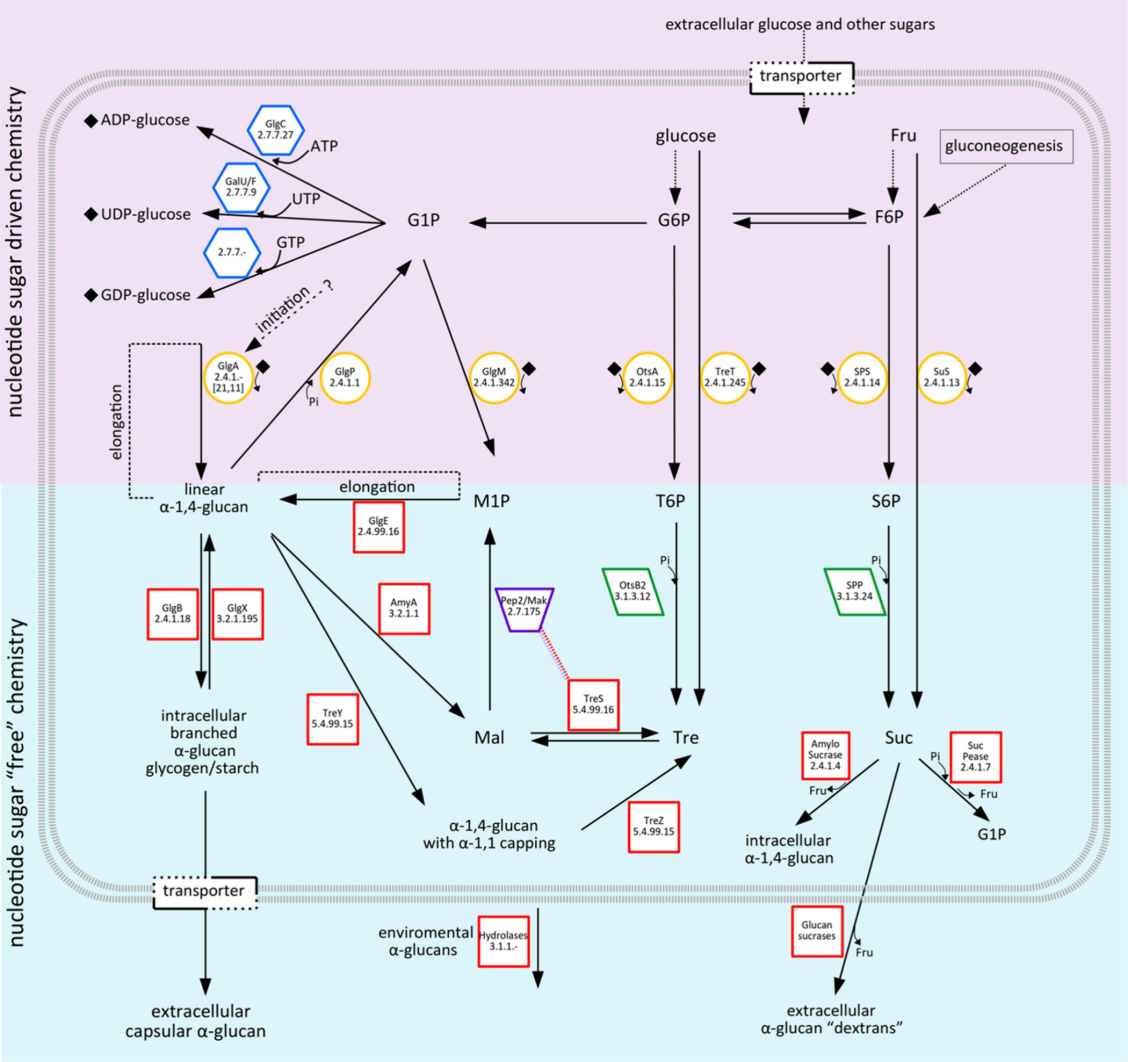
Integrated summary of α-glucan metabolic pathways in bacteria. The scheme reflects the overlay of different metabolic routes and enzymes discussed, evoking the idea of an underlying common theme supported by the evolution of an ancestral metabolism. The prokaryotic cell is depicted as enclosed by its membrane (double dotted boundary line) where glucose and other sugars enter and are transformed along the gluconeogenesis pathway eventually leading to the synthesis of glucose 1-phosphate, which is subsequently activated by forming sugar donors NDP-glucose (ADP-glucose or UDP-glucose). Different pathways for intra- and extracellular α-glucan synthesis depart from top to bottom, traversing the synthesis of glucose disaccharides. The area of the metabolism comprises reactions requiring nucleotide-sugar donors (violet background shade) and independent nucleotide-sugar (light blue background shade). The parallel biosynthetic routes follow similar transformations catalyzed by enzymes with typical catalytic domain architecture. Rossmann folds enzymes, preeminently NDP-sugar transferases (blue hexagons), and GT-B fold (yellow circles) drive transformation involving nucleotide derivatives. In contrast, TIM barrel folds (red boxes) take control of all nucleotide-independent transformations. Other intermediate reactions are driven by HAD domain phosphorylases (green parallelograms), which catalyze disaccharide dephosphorylation, and the phosphorylation of maltose carried out by the Maltokinase (violet trapezium).

The joint analysis of the α-glucan and glucose disaccharide biosynthetic pathways reveals some interesting relationships. At first glance, the synthesis of these molecules follows a similar overall ‘vertical’ pathway comprising (i) the nucleotide-glucose synthesis mediated by NSPases, (ii) the synthesis of an α-glucosyl-bond mediated GT-B fold enzymes, that may be or not be followed by the removal of phosphate by a phosphatase, and (iii) the editing and interconversion of the α-glucose moieties by TIM barrel enzymes. In that sense, only Pep2/Mak appears foreign to this picture and may represent a novelty in this metabolism acquired after LUCA, as suggested by the lack of detection of this enzyme in *Themotagae* and *Aquifex*.^228^ Interestingly, this order suggests that the grouping of the early part of the metabolism is mediated by Rossmann fold enzymes (nucleotide-sugar dependent metabolism), while the latest part of the metabolism is driven by the TIM barrel enzymes (nucleotide-sugar independent metabolism; Figure 18).

A horizontal view of the overall metabolism discloses the direction of evolutionary specialization of the different enzymes participating in the α-glucan and disaccharide pathways, suggesting the common ancestry of these enzymes. Interestingly, as we analyzed, in some species, the level of specialization can virtually separate these pathways; meanwhile, in others, it can be intertwined by the enzyme promiscuity, permitting a level of redundancy that can offer an evolutive advantage. This horizontal view also accounts for the specialization of GT-B disaccharide synthases. It can be speculated an ancestral enzyme to GlgM, a processive GS, and a processive GP that also acquired PLP to achieve the α-glucan depolymerization. In this general context, the synthesis of internal glycogen appears as a specialization that allows virtually enclosing this α-glucan energy storage broadly distributed in bacteria, implying an important evolutive advantage, whereas the GlgE pathway appears mostly centered on actinobacteria (Figure 18).^605^

Finally, TIM barrels appear associated to the intracellular and extracellular milieu, suggesting a primitive function of the corresponding polysaccharides associated with external energy sources or structural functions. Most of the interconversions mediated by the TIM barrel enzymes in this metabolism are phosphate-independent, and may be originated as part of the recent proposed ancient phosphate-free cryptic core metabolism.^606^ GlgE and S6FP are exceptions to this rule and their functions may have emerged in modern metabolism (Figure 18).

## 7 α-Glucans and Microbial Pathogenicity

Genes encoding enzymes involved in biosynthesis and mobilization of α-glucans are widespread among bacterial pathogens employing very diverse pathophysiological strategies. The specific contribution of α-glucan metabolism to virulence, pathogenesis, and potentially immune evasion has been investigated for several important pathogenic bacteria, and our knowledge on these aspects is summarized in the following sub-sections.

### 7.1 Mycobacterium Tuberculosis

The human pathogen *M. tuberculosis* produces α(1→4)-linked and α(1→6)-branched glycogen-like α-glucan exclusively via the GlgE pathway (Figure 3B). Two alternative routes, the TreS-Pep2/Mak and the GlgC-GlgM pathways, provide the substrate maltose 1-phosphate that is converted to the branched polymer by iterative cooperativity between the maltosyltransferase GlgE and the branching enzyme GlgB. The polymer is synthesized intracellularly, and then a portion of it is secreted by an unknown transport mechanism to form the major constituent of the capsular layer.^119^

Synthesis of glycogen-like α-glucan is relevant for pathogenesis of *M. tuberculosis* in two aspects. First, several genes involved in the α-glucan metabolic network are essential and, thus, represent potential drug target candidates. The maltosyltransferase GlgE was found to be essential because loss of enzymatic activity leads to intracellular build-up of its substrate maltose 1-phosphate, which is toxic to the bacterial cells for reasons that have not been fully elucidated yet.^212^ Similarly, the branching enzyme GlgB is essential because it is required for iterative production of the glycogen-like α-glucan polymer in cooperation with GlgE. Loss of GlgB enzymatic activity curtails GlgE activity since GlgE alone can only produce linear α(1→4)-linked maltooligosaccharides that become insoluble and provide less non-reducing ends than branched glucans to be extended by GlgE. Due to these reasons, inactivation of GlgB also causes accumulation of toxic levels of maltose 1-phosphate.^212^ In addition, a similar sugar phosphate-related toxicity supports essentiality of the trehalose 6-phosphate phosphatase OtsB2 in *M. tuberculosis*, which produces the trehalose substrate for the TreS-Pep2/Mak pathway, as inactivation causes accumulation of its substrate trehalose 6-phosphate.^225^ Furthermore, while the genes encoding trehalose 6-phosphate synthase OtsA and maltose 1-phosphate-producing glucosyltransferase GlgM are individually dispensable, their combined inactivation is synergistically lethal likely because of accumulation of their joined substrate ADP-glucose reaching toxic levels.^119^

Second, glycogen-like α-glucan was shown to be important for full virulence of *M. tuberculosis* in a mouse infection model. A mutant lacking the gene encoding maltose 1-phosphate-producing glycosyltransferase GlgM (described as glycogen synthase GlgA before knowing the configuration of α-glucan metabolic pathways in *M. tuberculosis*) that has reduced levels of capsular α-glucan showed impaired virulence in mice.^82^ With the recent elucidation of the complete metabolic network and pathways required for α-glucan production in *M. tuberculosis*, it was possible to generate defined mutants completely devoid of α-glucan in a rational way. This was mediated by blocking maltose 1-phosphate synthesis in a Δ*glgC* Δ*treS* double mutant. This double mutant exhibited significant reduction in virulence in mice in contrast to the corresponding single mutants. Since these mutations affected both intracellular and capsular α-glucan likewise, which likely fulfill different biological functions, it is unknown to which degree the loss of each α-glucan type contributed to the observed attenuation phenotype. As the outermost layer of the mycobacterial cell wall and first line of contact with host cells, capsular α-glucans may be important for *M. tuberculosis* pathogenesis by interacting with mammalian host cells and influencing the immune response to *M. tuberculosis*. In fact, *in vitro* experiments using purified capsular α-glucan from mycobacteria demonstrated that it can interact with complement receptor 3, thus mediating binding of *M. tuberculosis* to phagocytic cells.^607,608^ Capsular α-glucan has also been reported to block dendritic cell functions^609^ and to interact with the C-type lectin receptor DC-SIGN on dendritic cells.^610^ While it is intriguing to speculate that *M. tuberculosis* uses capsular α-glucans to modulate and evade antimicrobial immune effector functions of phagocytic cells, there is a puzzling complexity and reciprocal promiscuity regarding interaction of mycobacterial capsule components with immune receptors, i.e., one molecule can be a ligand for several receptors and one receptor can be triggered by several ligands.^611^ Unless the still pending discovery of the mechanism of secretion will allow the generation of *M. tuberculosis* mutants specifically defective only in capsular glycogen-like α-glucans that still produce normal levels of the intracellular polymer, the precise function and importance of capsular glycogen-like α-glucans for pathogenesis of *M. tuberculosis* and other pathogenic mycobacteria remain elusive.

### 7.2 Chlamydiae

Chlamydiae are Gram-negative bacteria that employ an obligate intracellular lifestyle, which is linked to a massive genome reduction and a biphasic developmental cycle that includes two distinct stages: the elementary body and the reticulate body. While the elementary body is a non-dividing and infectious form adapted to extracellular survival, the reticulate body constitutes a replicating form that is surrounded by a membrane forming an inclusion.^612−614^ The order Chlamydiales includes the *Chlamydiaceae* family and several family-level lineages collectively called *Chlamydia*-related bacteria.^615^ The *Chlamydiaceae* family includes human pathogens such as *Chlamydia trachomatis*, which is the most common sexually transmitted infection leading to infertility, extrauterine pregnancy, and miscarriages in females; *Chlamydia pneumoniae*, which causes acute respiratory diseases of the upper and lower respiratory tract, such as pneumonia and bronchitis; and *Chlamydia psittaci*, which is a leading cause of zoonotic avian chlamydiosis and mainly causes respiratory infections followed by systemic dissemination to the heart, liver, and gastro-intestinal tract. Furthermore, different species of the *Chlamydiaceae* family can also infect different animals such as *Chlamydia muridarum*, which infects rodents.^616^

While for other intracellular bacteria the adaptation to a strict intracellular life-style led to the loss of glycogen metabolism pathway,^557^ nearly all sequenced chlamydial genomes with the exception of *Criblamydiaceae* and *Waddliaceae* families maintained the GlgC-GlgA-GlgB dependent glycogen pathway.^222^*Chlamydia trachomatis* and the closely related rodent pathogen *Chlamydia muridarum* are unique amongst Chlamydiae phylum as they accumulate α(1→4)-linked and α(1→6)-branched glycogen-like α-glucan not only intracellularly but also extracellularly in the inclusion lumen.^617,618^ Glycogen accumulates first in the inclusion lumen and only later in elementary bodies. While extracellular glycogen deposition was initially thought to result from bacterial cell lysis,^617^ it was recently shown to represent a specific process involving two mechanisms.^619^ First, the host glycogen synthase Gys1, which is known to tightly bind to glycogen,^109^ is translocated into the vacuole, resulting in concomitant bulk import of host glycogen. Translocation of cytoplasmic glycogen likely occurs through invagination of the inclusion membrane, but the underlying mechanisms remain elusive yet. Bulk uptake of host-derived glycogen is a minor pathway for glycogen accumulation in the inclusion lumen and contributes only to ca. 20% of total glycogen accumulation.^619^ In contrast, the main source of intraluminal glycogen accumulation was found to result from de novo synthesis of glycogen that is mediated by secretion of the glycogen synthase GlgA^620^ and the branching enzyme GlgB^621^ by the bacteria inside the inclusion. Furthermore, the host transporter SLC35D2 and possibly further transporters are recruited to the inclusion membrane to allow translocation of UDP-glucose from the host cell cytoplasm into the inclusion lumen.^619^ While glycogen synthase GlgA from other bacteria typically prefers ADP-glucose as the substrate, GlgA from *C. trachomatis* can also utilize host-derived UDP-glucose to mediate glycogen formation in the inclusion lumen.^619^ Consistently, in contrast to GlgA and GlgB, chlamydial GlgC was not found to be secreted and remains intracellularly in the bacterial cells, highlighting the fact that intraluminal glycogen synthesis relies on host-derived UDP-glucose as the substrate.^619^ While secretion of GlgA, GlgB, and other proteins into the inclusion lumen was reported to be mediated by the bacterial type 3 secretion system,^619^ another study suggested that *C. trachomatis* employs a plasmid-dependent and type 3 secretion system-independent mechanism to export GlgA.^622^ Early during infection, the chlamydial cell population largely consists of reticulate bodies, and intraluminal glycogen accumulation via *de novo* synthesis and bulk import sets in between 16 and 20 h post infection.^619^ Later during the infection cycle, reticulate bodies start converting into elementary bodies, which are associated with mobilization of intraluminal glycogen. For this, the chlamydial cells secrete the enzymes required for glycogen degradation, i.e., the debranching enzyme GlgX and the glycogen phosphorylase GlgP into the inclusion lumen, likely in a type 3 secretion system-dependent manner.^619^ The resulting depolymerization end product, glucose 1-phosphate, is converted to glucose 6-phosphate by the chlamydial phosphoglucomutase MrsA, which is secreted as well.^619^ Glucose 6-phosphate can be imported into *C. trachomatis* cells by the hexose phosphate transporter UhpC and can be used as source of energy and carbon. Later during conversion of reticulate into elementary bodies, the type 3 secretion system is turned off, allowing for intrabacterial activity of the glycogen metabolism enzymes, and glycogen accumulation in the bacteria.^619^ The temporal separation of glycogen synthesis and subsequent mobilization allows *C. trachomatis* to sequester a host metabolite and accumulate it extracellularly in an osmotic inert storage form and to rapidly depolymerize and convert it into a readily usable molecule for the bacterium. While the precise role and importance of glycogen synthesis and degradation for virulence of *C. trachomatis* has not been fully elucidated yet, it was demonstrated that loss of the *glgA* gene in *C. muridarum* resulted in a significant reduction of pathogenicity in the genital tract in a mouse infection model, demonstrating the role of GlgA in *C. muridarum*-mediated induction of hydrosalpinx in mice, which is similar to the pathophysiology of *C. trachomatis* infection in women. The reduced infectivity of the *C. muridarum glgA* mutant was associated with a decreased ability to trigger inflammation.^623^ Consistent with this finding, treatment of human monocytic cell line THP-1 with GlgA protein from *C. trachomatis* elicited the expression of proinflammatory cytokines interleukin-8 (IL-8), interleukin-1beta (IL-1β), and tumor necrosis factor alpha (TNF-α) in a TLR2- and TLR4-dependent manner.^624^

While nearly all sequenced chlamydial genomes comprise the genes for a fully functional GlgC-GlgA-GlgB dependent glycogen pathway,^222^ the *Criblamydiaceae* and *Waddliaceae* families form an exception since the GlgC-GlgA pathway is defective. This is due to genomic rearrangements causing deletion of both the *glgC* and *glgP* genes and a fusion of the *glgA* and *glgB* genes rendering the branching enzyme domain inactive.^222^ Instead, a complete and functional GlgE pathway was identified in five chlamydial species distributed in *Criblamydiaceae*, *Waddliaceae*, and *Parachlamydiaceae* families including the emerging human pathogen *Waddlia chondrophila*,^625,626^ comprising genes encoding the maltosyltransfrase GlgE, the branching enzyme GlgB2, and a bifunctional TreS-Mak fusion protein, mediating glycogen biosynthesis from trehalose.^222^ Furthermore, these organisms also encode the enzymatic machinery for degradation of glycogen comprising glycogen phosphorylase GlgP2, debranching enzyme GlgX, and α(1→4)-glucanotransferase MalQ. Thus, glycogen metabolism is remarkably preserved also in environmental Chlamydiae suggesting an important function for their intracellular lifestyle.^222^ However, no specific glycogen-defective mutants are available yet to address the role a glycogen metabolism for infectivity and pathogenesis.

Altogether, while not strictly essential for viability, the capability to synthesize and to mobilize glycogen-like α-glucan appears to be a conserved metabolic feature of *Chlamydiae* that contributes to the virulence potential of pathogenic members of this phylum. This might represent a storage function particularly important regarding fueling essential metabolic pathways in the extracellular forms, i.e., elementary bodies of *Chlamydiales*.

### 7.3 *Pseudomonas aeruginosa*

*P. aeruginosa* is an important human pathogen and one of the most common causes of nosocomial infections. It is an opportunistic pathogen that primarily affects immunocompromised individuals, notably patients with immunodeficiencies and traumatic burn wounds. It is also a major pneumonia agent causing both acute and chronic lung infections, the latter particularly associated with cystic fibrosis and the ability of the bacterium to form biofilms.^627^ Due to its high intrinsic antibiotic resistance, *P. aeruginosa* infections are difficult to eradicate.^628^*P. aeruginosa* is also ubiquitously found in the environment in soil and aquatic habitats, and strains of *P. aeruginosa* as well as of related species such as *P. syringae* can be pathogenic to plants.^629,630^

*P. aeruginosa* is peculiar in producing α(1→4)-linked and α(1→6)-branched glycogen-like α-glucan employing both the GlgE pathway and the classical glycogen synthase GlgA as has recently been reported for strain PAO1 (Figure 3C).^173^ Production of glycogen through GlgA is surprising since *P. aeruginosa* PAO1 lacks AGPase GlgC. However, GlgA was shown to be specific for UDP-glucose and thus contrast to typical bacterial glycogen synthases that prefer ADP-glucose. UDP-glucose is produced in this strain from UTP and glucose 1-phosphate by the UGPase GalU to be used as substrate for GlgA and other enzymes.^173^ More surprisingly, it was shown that GlgA can produce linear α(1→4)-linked α-glucan with a DP >40, but this polymer is readily degraded and metabolized to trehalose employing the TreY-TreZ pathway in a cellular context before glycogen particles are formed.^173^ In fact, this is the only route to trehalose formation in *P. aeruginosa* PAO1, which lacks the OtsA-OtsB pathway that is dominant in most other bacteria. Part of the trehalose synthesized from GlgA-produced α(1→4)-linked α-glucan is then converted to α(1→4)-linked and α(1→6)-branched glycogen-like α-glucan by the GlgE pathway comprising a bifunctional TreS-Pep2 fusion protein producing maltose 1-phosphate from trehalose and ATP, the maltosyltransferase GlgE and the branching enzyme GlgB in a process very similar to the one described for *M. tuberculosis*.^173^ There is a second route to maltose 1-phosphate in *P. aeruginosa* PAO1 that involves the α-glucanotransferase MalQ, which can produce maltose by transferring a glucose moiety from the non-reducing end of a maltooligosaccharide when glucose is used as an acceptor molecule or when maltotriose is used as the donor substrate. The maltokinase activity of the TreS-Pep2 fusion protein can then yield maltose 1-phosphate in presence of ATP. Thus, MalQ is able to bypass the TreY-TreZ-TreS reactions to provide maltose 1-phosphate for the GlgE pathway.^173^ α(1→4)-linked and α(1→6)-branched glycogen-like α-glucan that accumulates in *P. aeruginosa* PAO1 is almost exclusively produced by the GlgE pathway, whereas linear α(1→4)-glucan produced by GlgA is demonstrated to accumulate only when TreY-TreZ is inactivated. Consistent with its role in trehalose production, the *glgA* gene is localized in an operon together with the *treY* and *treZ* genes in addition to *glgX* and *malQ*, separated from the GlgE pathway operon comprising the genes *treS*-*pep2*, *glgE*, and *glgB*, while the *glgP* gene is an orphan.^173^ It remains elusive why in *P. aeruginosa* PAO1 only the α(1→4)-glucan produced by GlgA is virtually quantitatively degraded and converted to trehalose, while the GlgE pathway produces polymeric glycogen particles. It appears to be a futile cycle to build linear α-glucan only for synthesis of trehalose and subsequently to produce α-glucan from it again. But in the face of a lack of the OtsA-OtsB route for trehalose, coexpression of the *glgA*, *treY*, and *treZ* genes from the same operon and possibly direct interaction of the corresponding proteins might enable efficient trehalose formation. Trehalose is not quantitatively, but only partially, converted to glycogen-like α-glucan and accumulates in the cytoplasm of the bacterial cells itself in response to certain stress conditions.^173^ Theoretically, specificity of GlgA for UDP-glucose might allow *P. aeruginosa* PAO1 to sequester this metabolite from host cells during infection similar to what has been described above for *C. trachomatis*. However, this would require the ability of *P. aeruginosa* PAO1 to take up extracellular UDP-glucose, but no such activity has ever been reported for this bacterium.

Both trehalose synthesized by the GlgA-TreY-TreZ route and glycogen-like α-glucan synthesized by the GlgE pathway play important, yet distinct, roles in stress protection in *P. aeruginosa* PAO1. Trehalose is specifically required for tolerance to osmotic stress, whereas the GlgE-derived α-glucan mediates desiccation tolerance. In turn, GlgE-derived α-glucan has a minor effect on osmotic sensitivity, while trehalose does not contribute directly to the desiccation response.^173^ Both trehalose and glycogen-like α-glucan were shown to be equally important for survival on abiotic surfaces.^173^ However, it remains to be elucidated to which degree each molecule contributes to virulence and pathogenesis of *P. aeruginosa* PAO1 in various infection models. Previously, deletion of the *treS* and *treY*-*treZ* genes, respectively, in *P. aeruginosa* strain PA14 was shown to abolish trehalose production and cause attenuation of virulence in a plant infection model, implicating trehalose as a stress protectant and possible virulence factor.^631^ Similarly, *treS* gene disruption in *P. syringae pv.* tomato resulted in osmotic sensitivity and attenuation during plant infection.^632^ However, the conclusions back at the time were drawn without knowledge of the full network and close interaction of the metabolism of trehalose and glycogen-like α-glucan in *Pseudomonas*. Given that the mentioned *Pseudomonas* strains very likely exhibit the same metabolic configuration as *P. aeruginosa* strain PAO1, the mutations in both cases would also lead to abolition of α-glucan biosynthesis. Thus, the virulence phenotypes previously attributed to trehalose could in fact rather be mediated at least partially by α-glucan. In contrast to the reported role in phytopathogenesis, deletions of the *treS* and *treY*-*treZ* genes in *P. aeruginosa* strain PA14 did not attenuate growth in a range of animal infection models.^631^ However, the used models were unlikely to reflect the significant osmolarity and desiccation stresses present during cystic fibrosis in humans. Therefore, the importance of glycogen-like α-glucan for human pathogenesis of *P. aeruginosa* remains to be fully investigated.

### 7.4 Biofilm-Forming Bacteria (*Streptococcus*, *Neisseria*, *Aeromonas*)

The ability to form biofilms is an important virulence trait of several pathogenic bacteria. While most of them produce an extracellular matrix composed of polysaccharides other than α-glucan, α-glucan is an important component of the EPS of bacterial biofilms related to dental plaque formation and caries development. Within the complex oral microbiome, the facultatively anaerobic gram-positive coccoid bacterium *S. mutans* is a major producer of EPS, the composition of which is complex and changes dynamically depending on food uptake. Within the mixture of EPS, extracellular DNA, and lipoteichoic acid, α-glucan can comprise between 10 and 20% of the dry weight of dental plaque.^633−637^ Within the complex and dynamically changing polymicrobial oral microbiome, *S. mutans* is not always the most abundant species, and many other bacteria contribute to acidogenesis and cariogenicity.^638,639^ However, *S. mutans* is a major EPS producer and rapidly modulates the formation of cariogenic biofilms when appropriate dietary substrates (i.e., sucrose and starch) are available.^640−643^ The role of extracellular α-glucan production by *S. mutans* in caries development has extensively been reviewed recently.^137^ Briefly, *S. mutans* secretes three different glucosyltransferases, which have distinct but partially overlapping, redundant functions. GtfB (formerly known as Gtf-I) and GtfC (Gtf-SI) are mutansucrases that synthesize water-insoluble mutan, which is α-d-glucan mainly composed of α(1→3) linkages.^644,645^ The secreted mutansucrases are incorporated into pellicle (particularly GtfC) but also adsorb on the bacterial cell surface (mainly GtfB), and both utilize dietary sucrose to synthesize water-insoluble mutan *in situ*. Furthermore, the secreted mutansucrases can also adsorb onto the surface of other oral microorganisms that do not produce glucosyltransferases themselves, turning them into mutan producers. The water-insoluble mutans facilitate adherence of microorganisms to the smooth surface of the tooth enamel. Concomitantly, salivary α-amylase in the pellicle digests dietary starch, releasing maltose and maltooligosaccharides that can serve as acceptors particularly for surface-adsorbed GtfB and are incorporated into the mutan polymer. *S. mutans* produces and secretes a third glucansucrase enzyme (GtfD, formely known as Gtf-S) catalyzing the synthesis of water-soluble dextran-like α-d-glucan mainly containing α(1→6) linkages,^646^ which can serve as primers for GtfB.^137^ The parallel formation of both water-soluble dextran and water-insoluble mutan enhances the adherence of the oral microorganisms to the smooth enamel surface and promotes dental plaque formation. Accordingly, it was shown that the sucrose-dependent adherence of *S. mutans* to the tooth surface was related to all three glucansucrase enzymes at an optimum ratio.^647^ The insoluble mutan molecules provide ample binding sites for *S. mutans* and other oral bacteria mediating tight bacterial clustering and adherence to the tooth enamel. This is supported by the secretion of several nonglucosyltransferase glucan-binding proteins, inactivation of which has been shown to result in impaired biofilm formation and altered architecture.^83^ The α-glucan-containing EPS formed in situ enables the assembly of a spatially heterogeneous and cohesive multicellular structure and development of a cariogenic biofilm.^648^ The importance of α-glucan production for virulence and cariogenicity of *S. mutants* has been demonstrated by studies of mutants lacking glucansucrase activities. Inactivation of any of the glucansucrases GtfB, GtfC, or GtfD resulted in a reduction of smooth-surface carious lesions in a rat model system.^649^ Similarly, although the nature of mutations is not known, glucan synthesis-defective mutants of *S. mutans* were shown to cause reduced caries development in rats.^650,651^

In addition to extracellular mutan and dextran-like α-glucan, *S. mutans* also produces intracellular glycogen employing the classical GlgC-GlgA pathway, which may also be important for virulence. A mutant defective in intracellular glycogen accumulation exhibited reduced cariogenic potential in rats.^652^ Conversely, a transposon mutant overproducing glycogen showed increased cariogenicity.^653^ However, it remains unclear whether glycogen accumulation contributes to biofilm formation and how it can influence virulence of *S. mutans* during growth in an environment rich in carbohydrates.

Different *Neisseria* species such as *N. polysaccharea*, *N. mucosa*, and *N. perflava* can also be found in human dental plaque. *Neisseria* secrete a GH13 amylosucrase to produce an extracellular glycogen-like polymer mainly composed of α(1→4) linkages with few α(1→6) branches.^161−164^ While this suggests that extracellular glucan produced by these *Neisseria* contribute to biofilm and dental plaque formation, no glucan deficient mutants have been described to address their relevance for inhabiting the oral environment.

Members of the genus *Aeromonas* are gram-negative, water-borne bacteria that are ubiquitously found in aquatic environments. As many strains are able to grow and to produce exotoxins at low temperatures, they are mostly infective to poikilothermic animals, but some species are also emerging as human pathogens causing systemic as well as gastrointestinal infections.^654^*Aeromonas* species produce a surface α-glucan consisting of α(1→4)-linked glucosyl units with α(1→6)-branches that is intracellularly synthesized by the UGPase GlgC and the glycogen synthase GlgA before being exported in a WecP-dependent fashion.^166−168^ The surface-exposed glycogen-like α-glucan is important for biofilm formation as was revealed by a GlgA deficient mutant of *A. hydrophila*.^168^ While this suggests that glycogen-like α-glucan may contribute to virulence of this species, no studies in relevant infection models have been performed to address this hypothesis.

### 7.5 Enteric Bacteria (*Escherichia coli*, *Salmonella enterica*, *Vibrio cholerae*)

Despite providing a mucus layer rich in carbohydrates, lipids, and proteins, the mammalian gastrointestinal tract represents an environment characterized by fluctuating availability of nutrients. Thus, both commensal and pathogenic bacteria in the gut are regularly facing prolonged periods of restricted nutrient availability and even starvation.^655,656^ To cope with a nutrient-limiting environment and to compete with other microorganisms, the ability to accumulate intracellular carbon and energy storages during nutrient-rich periods and to remobilize them during hunger phases is believed to be essential for persistence of intestinal bacteria. Glycogen is thought to be the primary carbon and energy storage molecule for enteric bacteria that employ the classical GlgC-GlgA pathway for glycogen synthesis (Figure 3A).^464^ Furthermore, after shedding with feces, glycogen may be important for extracellular survival and possibly promotes dissemination of pathogenic enteric bacteria.^93^

To assess the importance of glycogen biosynthesis and degradation in *E. coli*, Δ*glgA* and Δ*glgP* mutants were assessed in a streptomycin-treated mouse infection model and revealed that both are necessary for efficient colonization of the intestine by *E. coli*.^657^ In contrast, conflicting data exist regarding the role of glycogen metabolism for virulence of the closely related gram-negative bacterium *S. enterica*. Supporting the hypothesis of a central role of glycogen for virulence and infectivity of enteric bacteria, one study showed a correlation between the amount of glycogen stored during the preincubation step and the 50% lethal dose in for *S. enterica serovar Enteritidis* in a chicken infection model.^544^ In contrast, in another study, no significant differences in intestinal colonization or virulence in chicken were observed for deletion mutants in the *glgC* gene encoding AGPase generated in three different *S. enterica* serovars (*Gallinarum*, *Pullorum*, *Typhimurium*).^553^ However, while the *glgC* mutant strains were thought to be deficient in glycogen accumulation because they cannot make the glycogen precursor ADP-glucose, more recent findings suggest that *glgC* mutants of *E. coli* and *S. enterica* serovar *Typhimurium* possess an alternative pathway for ADP-glucose formation and still can accumulate glycogen under certain conditions.^658^ While this alternative route has not been identified and characterized enzymatically yet, it is possible that the importance of glycogen accumulation for virulence and infectivity in *E. coli* and *S. enterica* is underestimated when the assessment is based on the phenotype of GlgC-deficient mutants. In favor of this, studies with mutants of the pathogenic enteric bacterium *Vibrio cholerae* defective in glycogen synthesis or degradation support the importance of glycogen metabolism for pathogenesis.^95^ Like other enteric bacteria, *V. cholerae* employs the classical GlgC-GlgA pathway for glycogen synthesis but is peculiar in possessing two genes coding for AGPase, *glgC1* and *glgC2*. *V. cholerae* mutants defective in glycogen biosynthesis (Δ*glgC1* Δ*glgC2* double mutant) or degradation (Δ*glgX*) demonstrated that glycogen prolongs survival in nutrient-poor environments that are known ecological niches of *V. cholerae*, including pond water and rice-water stool. Additionally, defects in glycogen metabolism strongly attenuated pathogenesis of *V. cholerae* in an infant mouse transmission model of cholera. Lending further credence to the hypothesis of glycogen being an important contributor to virulence, glycogen granules can be found inside bacterial cells present in rice-water stool from cholera patients.^95^ Together, these findings implicate glycogen metabolism as a relevant survival strategy employed by enteric bacteria to survive the dramatic changes encountered in the host and the environment. This not only applies to pathogenic enteric bacteria, since glycogen has also been shown to support gut retention of probiotic bacteria in a germ-free mouse model, yielding a significant fitness disadvantage of a Δ*glgA* mutant strain of the gram-positive lactic acid bacterium *Lactobacillus acidophilus*.^659^

## 8 Concluding Remarks

More than a century of lessons about the biosynthesis of α-glucans has disclosed some functional roles and some patterns in the metabolism of these polysaccharides in bacteria. As we highlight in this Review, we show how bacterial metabolism evolved to handle the α-d-glucose scaffold to build assorted α-glucans with different functions, including energy storage or extracellular structural components as key elements in matrix biofilms formation, which provide protection to colonizing bacteria, and also as virulence factors. Taken together, the chemical landscape from α-d-glucose to disaccharides and α-glucans, the analysis of their metabolism and the structural/functional diversity of the intervenient enzymes, we provide an overall picture reflecting the underlying evolution of such enzymatic machinery (Figure 18). Our current view unveils a complexity in α-glucans diversity, although as we delve deeper into the fundamentals of heterogeneity, we encounter a nuanced landscape formed by few enzymatic folds linked by the evolution of the metabolism. Further examining the synergies of non-catalytic domains, including CBMs, for such α-glucans diversity, highlights the need for further research.

There is still an immense unexplored metabolic diversity landscape in the biosphere, likely influenced differently by such polymers in their niche and their metabolic blueprint. For example, the human gut microbiome, arguably the most studied ecosystem, is composed of ca. 3500 species,^660^ whose population composition is affected by the structure of dietary α-glucans.^661^ This is due to the different arsenal of enzymes of the diverse bacterial species for their processing;^662^ therefore, environmental α-glucans architecture acts as an environmental selective pressure, a fact that is already used to shift bacterial populations as medical treatment.^663,664^ In that sense, of special relevance is the so-called resistant starch, a type of starch that resists digestion in the small intestine, progressing to the large intestine where it acts as a prebiotic, feeding the beneficial bacteria in the gut microbiota.^674,675^ The use of resistant starch by gut bacteria involves several degradation steps, favoring the production of short-chain fatty acids such as butyrate that serves as a primary energy source for colon cells.^676^ Importantly, this process helps to maintain a healthy balance of gut bacteria, contributes to gut health by promoting bowel regularity, and can enhance the immune system. Therefore, foods high in resistant starch are indicated in a balanced diet for maintaining overall health. Extrapolating this to the biosphere, where diverse calculations predict the existence of ca. 9 million eukaryotic species and ca. 1 trillion microbial species,^665,666^ the dimension of the biosphere metabolic landscape suggests that we are only observing the tip of the iceberg. Moreover, most of our knowledge on α-glucans metabolism belongs to bacteria species cultured in the laboratory; nevertheless, the existence of the so-called “microbiological dark matter” represents one-quarter of the population of microbes belonging to phyla with no cultured relatives,^667^ indicating that future research will greatly expand on this metabolism architecture, possibly finding different rewirings of their metabolism, also offering an immense repertoire of new biotechnological opportunities.
